# Past-year intimate partner violence perpetration among people with and without depression: an individual participant data (IPD) meta-mediation analysis

**DOI:** 10.1007/s00127-021-02183-w

**Published:** 2021-11-29

**Authors:** Katherine R. K. Saunders, Sabine Landau, Louise M. Howard, Helen L. Fisher, Louise Arseneault, Geraldine F. H. McLeod, Sian Oram

**Affiliations:** 1 Health Service and Population Research Department, Institute of Psychiatry, Psychology and Neuroscience, King’s College London, London, UK; 2 Biostatistics and Health Informatics Department, Institute of Psychiatry, Psychology and Neuroscience, King’s College London, London, UK; 3 Social, Genetic and Developmental Psychiatry Centre, Institute of Psychiatry, Psychology and Neuroscience, King’s College London, London, UK; 4 ESRC Centre for Society and Mental Health, King’s College London, London, UK; 5 Department of Psychological Medicine, University of Otago, Christchurch, New Zealand

**Keywords:** Depression, Intimate partner violence, Systematic review, Meta-analysis, Individual participant data meta-analysis

## Abstract

**Purpose:**

To investigate whether (1) depression is associated with increased risk of past-year intimate partner violence (IPV) perpetration, disaggregated by sex, after controlling for potential confounders; (2) observed associations are mediated by alcohol misuse or past-year IPV victimisation.

**Methods:**

Systematic review and individual participant data (IPD) meta-mediation analysis of general population surveys of participants aged 16 years or older, that were conducted in a high-income country setting, and measured mental disorder and IPV perpetration in the last 12 months.

**Results:**

Four datasets contributed to meta-mediation analyses, with a combined sample of 12,679 participants. Depression was associated with a 7.4% and 4.8% proportion increase of past-year physical IPV perpetration among women and men, respectively. We found no evidence of mediation by alcohol misuse. Among women, past-year IPV victimisation mediated 45% of the total effect of depression on past-year IPV perpetration. Past-year severe IPV victimisation mediated 60% of the total effect of depression on past-year severe IPV perpetration. We could not investigate IPV victimisation as a mediator among men due to perfect prediction.

**Conclusions:**

Mental health services, criminal justice services, and domestic violence perpetrator programmes should be aware that depression is associated with increased risk of IPV perpetration. Interventions to reduce IPV victimisation might help prevent IPV perpetration by women. Data collection on mental disorder and IPV perpetration should be strengthened in future population-based surveys, with greater consistency of data collection across surveys, as only four studies were able to contribute to the meta-mediation analysis.

**Supplementary Information:**

The online version contains supplementary material available at 10.1007/s00127-021-02183-w.

## Background

Intimate partner violence (IPV) is the most commonly experienced form of violence worldwide [1], with adverse impacts for the health and wellbeing of victims, including children exposed to this form of abuse [2]. A variety of risk factors for perpetration of IPV have been identified including having a diagnosed mental disorder [3]. However, more research is needed to investigate associations between mental disorder and IPV perpetration independent of confounders and to explore possible mechanisms, including the potentially mediating roles of substance misuse and of IPV victimisation. Research is also needed to examine possible sex differences in associations between mental disorders and IPV perpetration and the effects of potential mediators.

The vast majority of individuals experiencing mental disorders are not violent, and people with mental disorders are more likely to experience than to perpetrate IPV [4]. However, in a systematic review of predominantly cross-sectional studies, mental disorders were shown to be associated with lifetime perpetration of IPV among both men and women [5]. Associations were reported across a range of diagnoses (including depression, anxiety, panic disorder, and post-traumatic disorder). Due to a lack of data on recent IPV perpetration, however, the review drew a few conclusions about whether a contemporaneous association exists between mental disorder and IPV perpetration. It also could not account for potential confounders of the observed associations. More recently, a population-based, sibling-controlled longitudinal study found increased risks of IPV perpetration among people with substance use disorders, depression, anxiety, schizophrenia-spectrum disorder, bipolar disorder, and attention deficit hyperactivity disorder (ADHD), with the schizophrenia-spectrum disorder link apparently confounded by familial factors [3]. Evidence of a positive association between ADHD and IPV perpetration was also reported in a systematic review of cohort and case–control studies, although some studies did not control for the presence of comorbid conduct disorder or antisocial personality disorder and the results were not disaggregated by sex [6].

Greater understanding of mediational pathways may assist with the development of interventions to reduce the risk of IPV perpetration through the identification of potentially modifiable factors. Previous research highlights substance misuse and of IPV victimisation as potential mediators of associations between mental disorders and IPV perpetration. For example, longitudinal analyses of Swedish registry data have demonstrated that while mental disorders were associated with increased risk of IPV perpetration, the highest absolute and relative risks were found where there was a principal or comorbid diagnosis of substance use disorder [3]. Similarly, analysis of longitudinal psychiatric morbidity survey data from the USA has demonstrated that there was a modest relationship between general violence and mental disorders, with a stronger relationship where there was comorbid substance abuse or dependence [7]. Mental disorder is known to increase risk of incident IPV victimisation [8], and studies have reported associations between IPV victimisation and perpetration [9, 10]. Although to our knowledge, the association between IPV victimisation and perpetration has not been investigated in the context of mental disorder, findings from the general violence literature point to a strong association between the perpetration of violence and violent victimisation among people with mental disorders [11, 12]. Analyses need also to take account of other potential confounders such as childhood maltreatment and abuse, poverty, and other social determinants, which increases risk of both mental disorder and IPV perpetration [13–15].

Against this background, the present analysis utilises combined data from four studies in an individual participant data (IPD) meta-mediation analysis [16] to address a series of issues relating the association between depression and IPV perpetration.

Specifically, we: (1) estimate the prevalence and difference in proportion of past-year IPV perpetration and among individuals with depression, compared to individuals without depression, disaggregated by sex, and controlling for potential confounders; (2) conduct mediation analyses to investigate whether alcohol misuse and IPV victimisation mediate the relationship between depression and past-year IPV perpetration.

## Methods

### Study design

Systematic review and IPD meta-mediation analysis. The IPD meta-mediation analysis is registered on PROSPERO (CRD42018082258). The PRISMA-IPD statement has been followed [17].

### Inclusion criteria

Potentially eligible studies for the IPD meta-mediation analysis were identified through systematic review searches. Studies were eligible to begin data harmonisation if: study participants were aged 16 and older; they measured depression using either a validated diagnostic tool, validated screening tool, or clinical diagnosis; they measured past-year IPV perpetration; and were representative general population surveys conducted in a high-income country setting. We excluded data from low-income country settings because of likely differences in contextual and societal factors relevant to both IPV perpetration and mental health, such as gender norms, the acceptability of gendered violence [18, 19], income inequality [20], access to healthcare [21], and political instability [22], which would have likely resulted in too much heterogeneity and thus prevented the proposed analyses from being conducted. Individual participant data needed to be available for re-analysis.

### Exposures, outcomes, and mediators of interest

The exposure of interest for this review was depression within the past 12 months, defined either in accordance with ICD [23] or DSM [24] criteria (any edition). Unexposed/comparison groups comprised individuals without depression. The outcomes of interest were physical IPV perpetration in the past 12 months, psychological IPV perpetration in the past 12 months, sexual IPV perpetration in the past 12 months, and coercive controlling behaviours in the past 12 months. IPV was defined using as any incident or pattern of incidents of controlling, coercive, threatening behaviour, violence, or abuse between those aged 16 or over who are, or have been, intimate partners regardless of gender or sexuality (UK Home Office, 2012). Mediators of interest were alcohol misuse and drug use within the past 12 months, and IPV victimisation within the past 12 months.

### Confounders and stratifiers

A number of variables were considered putative confounders as they might affect variables in two or more of the following categories: exposures, mediators, or outcomes. These were age, education level, income, relationship status, number of children, childhood abuse or maltreatment (experienced before the age of 18), ethnicity, employment, housing status, and other violent offending [14, 20, 25–30]. Separate mediation models were fitted for males and females; previous analyses have demonstrated that men are more likely to perpetrate and women are more likely to be the victims of IPV that is injurious, frequent, sexual, and occurring within a context of controlling and coercive behaviour [31].

### Search strategy

The search strategy and screening process for identifying studies for inclusion in the IPD meta-mediation analysis was a two-stage process. The first stage involved searching for papers for a systematic review and aggregate data meta-analysis (not reported here), and the second stage involved additional steps to identify studies that fulfilled eligibility criteria for the current IPD meta-mediation analysis. Details are provided in the supplementary information.

### Data access

Publicly available datasets were downloaded. Access was sought for datasets not in the public domain, either via application to data repositories or principal investigators. All contributing data were cross-sectional. Where studies collected data over multiple waves, one wave was chosen, selected on the basis of participant age (e.g., in which participants were more likely to be or have been in intimate relationships) and/or sample size.

### Data harmonisation

For each eligible study, the variables of interest (exposures, outcome, and hypothesized mediators), as well as variables (covariates) that might act as confounders or stratifiers were extracted from the IPD datasets provided and combined into a master database. To enable pooling, a number of data harmonisation rules were applied. Data harmonisation was only undertaken for outcome, exposure, and mediator variables for which data were available in at least three datasets, and for confounders for which data were available in all datasets being pooled. Details of the variables used for each exposure, outcome, and mediator are provided in the supplementary material, along with how each variable was assessed in each contributing study.

#### Outcome

Binary variables were created for past-year physical IPV. In addition to the outcome variables specified in the study protocol, we created a variable of past-year severe physical IPV perpetration; evidence suggests that depression is associated with severe IPV perpetration [32]. A binary variable was created in line with guidance for using the Conflict Tactics Scale-2 [33], adapted forms of which had been used by the included studies. Harmonised variables were not created for other forms of IPV (e.g., sexual, psychological), due to a lack of contributing data.

#### Exposures

Where not already dichotomised, the depression variable(s) for each dataset were made binary with reference to validated cut-offs scores for each assessment tool, i.e., the score above which a tool would indicate probable depression [34–36].

#### Mediators

Where not already dichotomised, alcohol misuse was dichotomised based either on self-reported problematic alcohol use, clinical diagnosis, validated screening tool cut-offs, or similar criteria used in validated measures. Drug use was dichotomised based on any positive endorsement of drug use in the past-year, self-reported problematic drug use, or an illicit substance use diagnosis. Where not already dichotomised, past-year IPV victimisation was made binary, based on endorsement of any item measuring IPV victimisation. Additional to the mediator variables specified in the study protocol, we created a variable of severe IPV victimisation, in line with guidance for using the Conflict Tactics Scale-2 [33].

#### Covariates

Insufficient data were available to include childhood abuse or maltreatment, employment, housing status, ethnicity, and violent offending as potential confounders. Relationship status was removed as a potential confounder due to problems of perfect prediction. The covariates which were included were sex, age, education, income, and number of children. Age and number of children were used as continuous variables. Education was used as a categorical variable, with data from each dataset recoded into the following categories: no education, high school qualifications, and post-school qualifications. Where data were available, participants who reported never having been in a relationship were dropped from the analysis. Income was used either as a continuous variable or, if income data were collected in bands, income was approximated as the midpoint of each band.

### Statistical analyses

Statistical modelling aimed to assess the effect of mental disorders on past-year IPV perpetration and investigate the presence of indirect effects via various hypothesized mediator variables. All analyses were stratified by sex. Mediators were considered separately. Separate analyses were conducted for each combination of exposure (depression), mediator (alcohol misuse, drug use, any past-year IPV victimisation, and severe IPV victimisation), and outcome variables (any physical IPV perpetration, and severe physical IPV perpetration). Age, education, income, relationship status, and number of children were controlled for as potential confounders in the meta-mediation analyses.

Two-stage IPD meta-mediation analyses were conducted. In the first stage, mediation analyses were run for each individual study, producing both total and direct and indirect effects of exposure on outcome, adjusted for potential confounders. In the second stage, study-level summary statistics for the total, direct, and indirect effects for each exposure, mediator, and outcome combination were combined using fixed-effects meta-analyses, using Stata’s <metan> command [37]. Effect heterogeneity was assessed using the *I*^2^ statistic. Sensitivity analyses explored the impact of using fixed- versus random-effects meta-analysis; the outputs were minimally different (see supplementary information).

The first stage of the IPD meta-mediation analyses was carried out in Stata using the command <medeff> [38], which uses a simulation approach to conduct causal mediation analysis and can accommodate binary mediators and outcomes [30]. For binary data, the parametric model assumed for the mediator and the outcome is a probit model. As a result, total, direct, and indirect effects are expressed as proportion differences. More specifically, here, the total effect refers to the total effect of depression on IPV perpetration in terms of proportion difference; the indirect effect refers to the part of that effect that is mediated by a given variable, and the direct effect refers to the part of the effect which is unmediated. Each of the total, direct, and indirect effects have been adjusted for confounders. Complete case analysis was used; that is, only participants who provided complete data on all exposure, mediator, and outcome variables were included in the modelling. Due to different levels of missing data across the three mediators, the total effect estimates differ slightly across the mediation analyses. A number of planned mediation analyses could not be run for individual datasets due to very low cell counts and perfect prediction of, for example, (i) IPV victimisation by IPV perpetration among men in the Christchurch Health and Development Study, (ii) depression by IPV victimisation among men in the National Survey for Families and Households and the Adult Psychiatric Morbidity Survey (APMS) 2014, and (iii) depression by IPV victimisation among men in the APMS dataset. This resulted in an inability to analyse drug use as a mediator for both men and women, and IPV victimisation as a mediator for men.

## Results

**Fig. 1:**
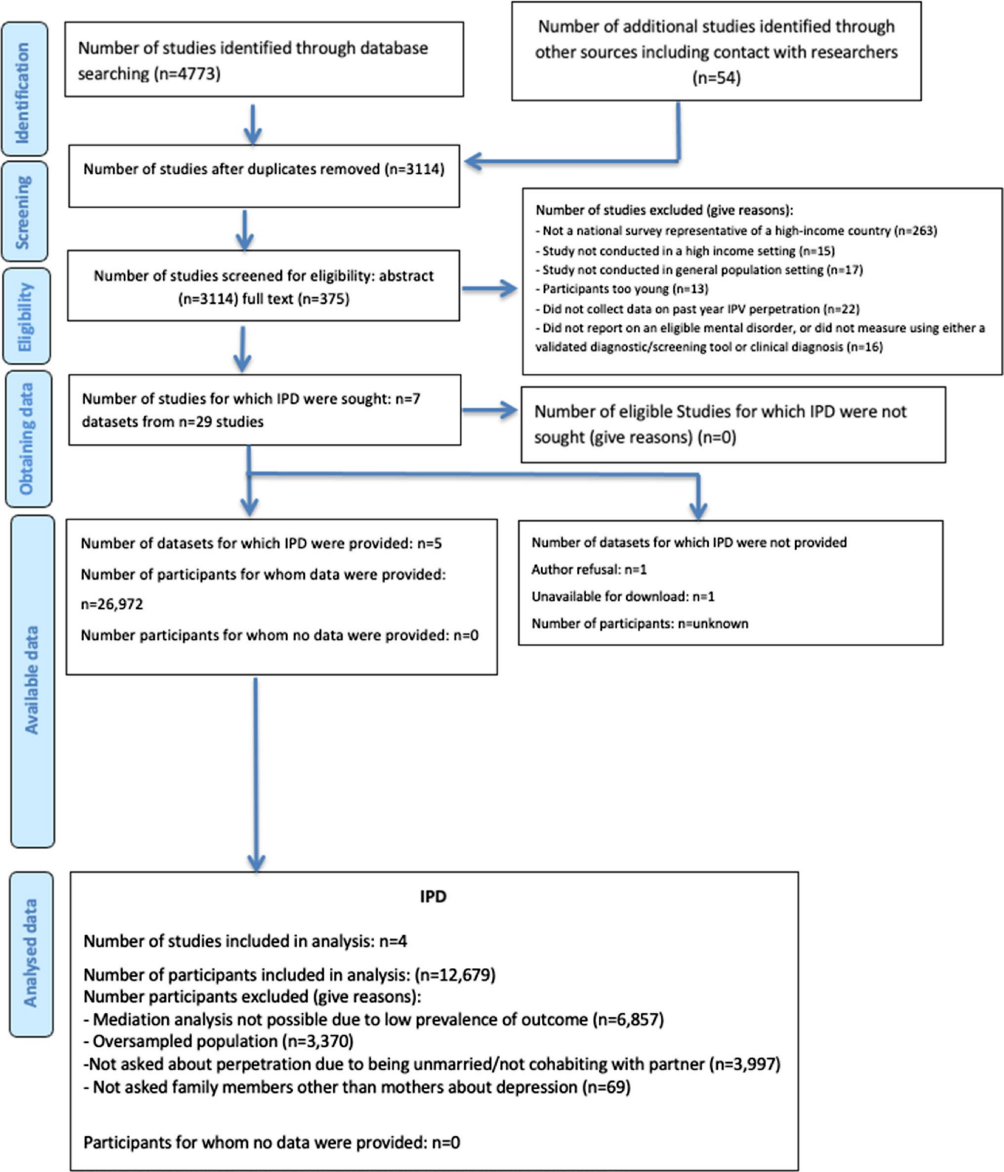
PRISMA individual participant data meta-mediation analysis flow diagram

We identified seven datasets eligible for inclusion; study characteristics are shown in Table 1. Of these, two datasets could not be included due to data not being shareable. Data on both depression and physical IPV perpetration were therefore available for a combined sample of 20,119 participants across five datasets. However, a third dataset could not contribute to meta-mediation analyses, because the low frequency of reported IPV perpetration created zero cell counts. Thus, four datasets contributed to the meta-mediation analyses, with a combined sample of 12,679 participants (Fig. 1).

**Table 1:** Characteristics of the eligible datasets

Dataset	Primary Publication (author, year)	Study design	Country	Description	Sample size (male/female)	Mean age (SD)	*N* (%) with depression
Environmental Risk (E-Risk) Longitudinal Twin Study	Moffitt and E-Risk Study Team, 2002 [61]	Prospective birth cohort study	England and Wales	Nationally representative birth cohort of families with twins born in 1994–95. The mothers of the twins were used for this analysis	1047 Male: 0 Female: 1047	38.9 (5.8)	107 (10.2)
National Survey of Families and Households	Sweet et al. 1988 [62]	Series of cross-sectional surveys, with retrospective sequences	USA	Nationally representative population-based survey of households in the USA	5640 M: 2604 F: 3036	43.6 (15.7)	1220 (21.6)
National Longitudinal Study of Adolescent to Adult Health (Add Health)	Harris and Udry, 2018 [63]	Prospective cohort study	USA	Nationally representative sample of U.S. adolescents in grades 7–12 between 1994–1995	5114 M: 2353 F: 2761	29.0 (1.8)	1591 (30.6)
Christchurch Health and Development Study	Fergusson and Horwood 2001 [64]	Prospective cohort study	New Zealand	Longitudinal study of all babies born in a four-month period in mid-1977 in Christchurch, NZ	878 M: 427 F: 451	Age 30 years in 2007	111 (12.6)
2014 Adult Psychiatric Morbidity Survey^a^	McManus et al. 2016 [65]	Series of cross-sectional surveys	England	Nationally representative population-based household survey of adults in England	6857 M: 2753 F: 4104	51.8 (18.3)	247 (3.2)
Dunedin Multidisciplinary Health and Development Sudy^2^	Poulton et al. 2015 [66] Silva, 1976 [67]	Prospective birth cohort study	New Zealand	Longitudinal study of babies born between April 1972 and March 1973	1037 M: 535 F: 502	Age 32 years in 2005	157/972 (16.2) (Ramrakha et al. 2013[68])
National Eidemiologic Survey of Alcohol and Related Conditions—wave II^b^	Grant et al. 2004 [69]	Prospective cohort study	USA	Nationally representative population-based survey of adults in USA	Unknown	Unknown	Unknown

^a^Dataset not included in mediation meta-analysis due to low frequency of reported perpetration (see supplementary information)

^b^Dataset not shared for inclusion in the review

Table 2 shows the prevalence of past-year perpetration of physical and severe physical IPV, disaggregated by sex. Estimates of the prevalence of physical and severe physical IPV perpetration varied across studies, but also by sex and by depression status. For both, reported prevalence was higher among women than among men; among women estimates of past-year physical IPV perpetration ranged from 0.9% to 28.4% while among men estimates ranged from to 3.1% to 8.0%. Reported prevalence was also higher among both women and men with depression versus women and men without depression.

**Table 2:** The prevalence of IPV perpetration in the past year, disaggregated by sex and by depression

	Physical IPV perpetration *n*/*N*			Severe physical IPV perpetration * n*/*N*		
	All	With depression	Without depression	All	With depression	Without depression
Women						
2014 Adult Psychiatric Morbidity Survey (APMS)^a^	33/3876 0.9%	3/150 2.9%	30/3726 0.8%	21/4060 0.5%	3/163 2.7%	18/3897 0.5%
Environmental Risk (E-Risk) Longitudinal Twin Study	297/1044 28.4%	48/105 45.7%	248/938 26.4%	112/1044 10.7%	25/105 23.8%	86/938 9.2%
National Survey of Families and Households	137/2565 4.5%	66/617 9.5%	66/1844 3.0%	26/2565 0.9%	12/617 1.6%	12/1844 0.6%
National Longitudinal Study of Adolescent to Adult Health (Add Health)	387/2668 14.0%	187/913 19.2%	200/1755 11.3%	257/2668 9.1%	122/913 11.8%	135/1755 7.7%
Christchurch Health and Development Study (CHDS)	41/451 9.1%	11/63 17.5%	30/388 7.7%	12/451 2.7%	3/63 4.8%	9/388 2.3%
Men						
2014 Adult Psychiatric Morbidity Survey (APMS)^a^	10/2592 0.5%	0/68 0%	10/2524 0.5%	4/2732 0.2%	0/79 0%	4/2653 0.2%
Environmental Risk (E-Rsk) Longitudinal Twin Study	–	–	–	–	–	–
National Srvey of Families and Huseholds	88/2251 3.1%	30/386 6.5%	57/1766 2.6%	17/2251 0.6%	6/386 1.3%	10/1766 0.5%
National Longitudinal Study of Adolescent to Adult Health (Add Health)	163/2265 6.8%	69/602 11.8%	94/1663 5.1%	70/2265 3.2%	31/602 5.8%	39/1663 2.3%
Christchurch Health and Development Study (CHDS)	34/427 8.0%	6/48 12.5%	28/379 7.4%	7/427 1.6%	0/48 0%	7/379 1.8%

Any discrepancies between proportions and percentages are due to weighting some of the datasets for percentage calculation

^a^Dataset not included in mediation meta-analysis due to low frequency of reported perpetration (see supplementary information)

**Fig. 2:**
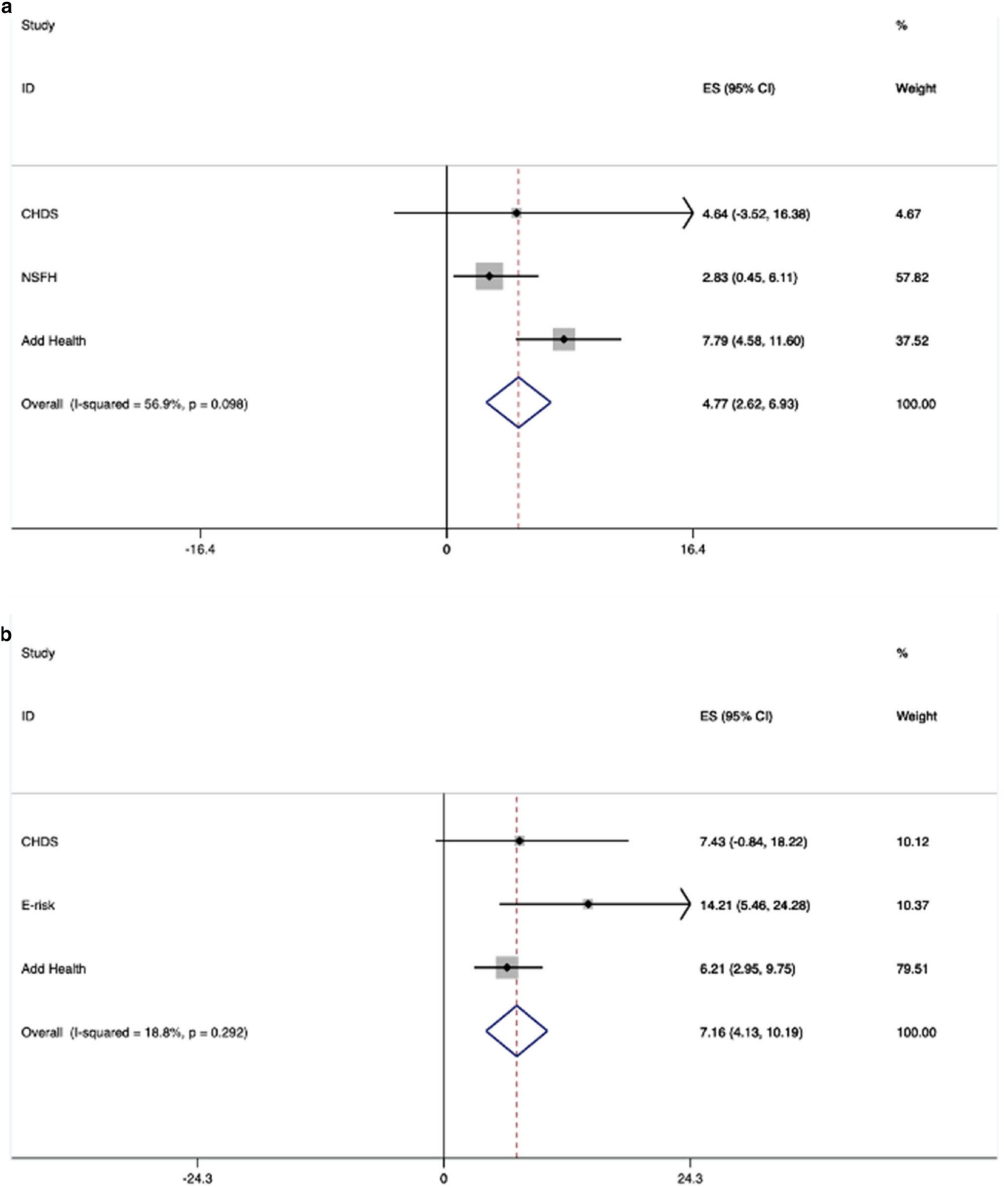
**a** (top) Forest plot showing the total effect of depression on past-year physical IPV perpetration among men. **b** (bottom) Forest plot showing the total effect of depression on past-year physical IPV perpetration among women. *CHDS* - The Christchurch Health and Development Study, *NSFH* - National Survey of Families and Households, *Add Health* - The National Longitudinal Study of Adolescent Health, *E-Risk* - Environmental Risk Longitudinal Twin Study

Figure 2 shows the forest plot for total effects of depression on physical IPV perpetration for women and for men. The forest plots show the pooled effect, indicated by the dotted line in the centre of each diamond, and confidence intervals are denoted by the two points on the left and right of the diamond. The line at zero denotes ‘no proportion difference’. An increase in the past-year perpetration of physical IPV among people with depression versus people with no depression was seen for both women (7.2.%, 95% CI 4.1–10.2%, *p* < 0.001) and men (4.8%, 95% CI 2.6–6.9%, *p* < 0.001).

As shown in Table 3, there was no evidence that alcohol misuse mediated the relationship between depression and IPV perpetration among women. Approximately 45% of the total effect (3.3%, 95% CI 2.0–4.5%, *p* < 0.001) was mediated by IPV victimisation, but there was considerable heterogeneity in the indirect effect (*I*^2^ 82.6%). Approximately 25% (1.9%, 95% CI 0.7–3.2%, *I*^2^ 0.0%, *p* = 0.002) of the total effect was mediated by severe IPV victimisation. The equivalent analyses for perpetration of severe physical IPV by women are also shown in Table 3. Across the two meta-mediation analyses, the increase in the past-year perpetration of severe physical IPV among women with depression versus women with no depression was between 2.0–4.0%. Approximately half of this total effect (2.1%, 95% CI 1.1–3.1%, *p* < 0.001) was mediated by IPV victimisation, though there was considerable heterogeneity (*I*^2^ 85.4%). Approximately 60% (1.2%, 95% CI 0.6–1.8%, *I*^2^ 0.0%, *p* < 0.001) of the total effect was mediated by severe IPV victimisation.

**Table 3:** The total, direct, and indirect effects of depression on physical intimate partner violence (IPV) perpetration for selected mediators among women^a^

Mediator	Total effect^b^ % (95% CI)	Direct effect % (95% CI)	Indirect effect % (95% CI)
Physical intimate partner violence (IPV) perpetration			
Alcohol misuse (*n* = 3927)	7.2 (4.1, 10.2) *I*^b^ 18.8%, *p *= 0.29 *z *= 4.63 *p *< 0.001	6.8 (3.8, 9.8) *I*^b^ 0.0%, *p *= 0.55 *z *= 4.42 *p *< 0.001	0.04 (− 0.1, 0.2) *I*^b^ 68.1%, *p *= 0.04 *z *= 0.61 *p *= 0.540
IPV victimisation (*n* = 3947)	7.4 (4.3, 10.6) *I*^b^ 29.2%, *p *= 0.24 *z *= 4.62 *p *< 0.001	3.1 (0.3, 6.0) *I*^b^ 0.0%, *p *= 0.77 *z *= 2.18 *p *= 0.029	3.3 (2.0, 4.5) *I*^b^ 82.6%, *p *= 0.003 *z *= 4.94 *p *< 0.001
Severe IPV victimisation (*n* = 3946)	7.4 (4.3, 10.4) *I*^b^ 17.7%, *p *= 0.297 *z *= 4.65 *p *< 0.001	5.4 (2.5, 8.2) *I*^b^ 0.0%, *p *= 0.46 *z *= 3.67 *p *< 0.001	1.9 (0.7, 3.2) *I*^b^ 0.0%, *p *= 0.67 *z *= 3.03 *p *= 0.002
Severe physical IPV perpetration			
Alcohol misuse	Not available	Not available	Not available
IPV victimisation (*n* = 3947)	4.0 (1.5, 6.6) *I*^b^ 58.8%, *p *= 0.09 *z *= 3.09 *p *= 0.002	1.3 (− 1.0, 3.5) *I*^b^ 0.0%, *p *= 0.42 *z *= 1.08 *p *= 0.282	2.1 (1.1, 3.1) *I*^b^ 85.4%, *p *= 0.001 *z *= 4.07 *p *= < 0.001
Severe IPV victimisation (*n* = 5840)	2.0 (0.6, 3.3) *I*^b^ 60.3%, *p *= 0.06 *z *= 2.92 *p *= 0.004	0.9 (− 0.4, 2.1) *I*^b^ 45.1%, *p *= 0.14 *z *= 1.38 *p *= 0.169	1.2 (0.6, 1.8) *I*^b^ 0.0%, *p *= 0.49 *z *= 3.72 *p *< 0.001

^a^All analyses adjusted for potential confounders

^b^These estimates vary slightly as they are estimated based on slightly different samples due to using a complete case analysis

We found no evidence that alcohol misuse mediated the relationship between depression and IPV perpetration in men (0.02%, 95% CI − 0.1–0.2%, *p* = 0.21). We could not investigate IPV victimisation as a potential mediator of the relationship between depression and IPV perpetration in men due to perfect prediction of either the exposure of outcome by the mediator.

## Discussion

### Key findings

We found that a higher proportion of men and women with depression report physical IPV perpetration in the past year compared to men and women without depression, after controlling for confounders. Across all studies for which data were available, reported prevalence of past-year physical IPV perpetration was higher for women (0.9–28.4%) than it was for men (0.5–8.0%). The same pattern was observed for past-year severe physical IPV perpetration for women (0.5%-10.7%) and men (0.2–3.2%). However, an insufficient number of studies reported data on other types of IPV for men and women, and for physical IPV perpetration, only four studies could contribute to this meta-mediation analysis, highlighting the lack of data available to test our hypotheses. In this meta-mediation analysis, we found no evidence, for either men or women, that alcohol misuse mediated the relationship between depression and physical IPV perpetration in the past year. Past-year IPV victimisation was found to mediate 45% of the total effect of depression and past-year perpetration of physical IPV among women. Also, among women, IPV victimisation was estimated to mediate between 50 and 60% the total effect of depression on past-year perpetration of severe physical IPV.

Depression has previously been shown to be associated with an increased risk of having ever perpetrated physical IPV [3, 5, 39]. This study extends these findings, demonstrating, first, that depression is also associated with having perpetrated physical IPV in the past year, and second, that the association persists after controlling for age, education, income, and number of children. Our finding of higher reported IPV perpetration among women than men has also been reported elsewhere [40–42]. Our analyses were unable to take account of the frequency, pattern, or context of physically violent acts, and may reflect misclassification bias; elsewhere, analyses have shown that the majority of injurious and high-frequency IPV is experienced by women [31]. Findings may reflect differential under-reporting among men versus women [43], whereby men who perpetrate IPV may seek to underplay their violent behaviour [44], although the mechanisms behind this are poorly understood.

Previous studies have shown that alcohol misuse is causally associated with both IPV perpetration [45] and depression [46, 47]. Whether alcohol misuse mediates the relationship between depression and past-year IPV perpetration has received limited previous attention, but analysis of longitudinal data from the National Epidemiologic Survey on Alcohol and Related Conditions (NESARC) has shown that substance abuse comorbidity increased the risk of violence perpetration generally in people with mental disorder in a general population sample [48]. These findings contrast with the results of this analysis, which found no evidence that alcohol misuse mediated the relationship between depression and IPV perpetration. This apparent discrepancy may be due to misclassification bias (i.e., under-reporting of alcohol misuse and/or IPV perpetration); insufficient power to detect an association due to small numbers of those reporting all three of IPV perpetration, depression, and alcohol misuse; or high heterogeneity as a result of insufficient variable harmonisation. As there was significant variation in how alcohol misuse was measured across datasets, it is most likely that this result is due to insufficient data harmonisation. It may also be the case that alcohol misuse may confound rather than mediate the association between depression and past-year physical IPV perpetration.

IPV victimisation was found to explain a significant portion of the total effect of depression on IPV perpetration among women. This is in keeping with the findings of a systematic review of longitudinal data, which established that depression is associated with incident IPV victimisation among women [8], and findings that a high proportion of women who report the use of physical violence against intimate partners also report IPV victimisation [10, 49]. Research suggests that many couples experience bidirectional violence [13, 50], and that poor mental health, including depression, is more common where violence is bidirectional versus unidirectional [13, 15, 32, 51]. Self-defence and retaliation are commonly described motivators for women’s use of violence in intimate relationships [52], although this is contentious [42]. Straus (2010), for example, highlights research which has concluded that only a small portion of women’s IPV is explained by self-defence, and notes that most research does not collect the same data for men, and therefore, it cannot be assumed that women’s use of violence differs from men. In this study, we were not able to determine the context of either IPV perpetration or IPV victimisation. We were also not able to investigate whether the association between depression and past-year IPV perpetration was mediated by IPV victimisation for men. Previous analyses of cross-sectional data from Wave 1 of the National Survey of Families and Households (included in this IPD meta-mediation analysis) have shown, however, a stronger link between bidirectional IPV and depression among women than for men [13].

### Strengths and limitations

The review used a comprehensive search strategy, and took a systematic approach to data management and harmonisation. Analyses were conducted separately by sex, considered alcohol misuse and IPV victimisation as potential mediators of these relationships, and accounted for a number of key covariates. Eligibility was limited to studies that used samples that were representative of the general population and used validated diagnostic or screening measures of mental disorder, reducing the risk of selection and measurement bias. Datasets were included from several countries, broadening generalisability in a high-income country context.

However, several limitations should be noted. First, due to the use of cross-sectional data, the direction of the observed associations cannot be inferred. Although some longitudinal data were available, we could not conduct analysis of temporal associations between depression and IPV perpetration either, because studies did not measure past-year IPV perpetration and/or mental health at multiple time points, or they did not collect data on the same population in each wave, or because there was significant attrition between time points. Although our aim by analysing associations between mental disorder and IPV perpetration in the past 12 months was to establish that these occurred, if not concurrently, over a short period, findings should be considered preliminary, and tested in future models using longitudinal data. Second, due to limited availability of data, analyses of the association between mental disorder and IPV perpetration could be conducted only for depression, and some mediation analyses could not be carried out for data on men due to small numbers and perfect prediction. Previous research indicates that there is an increased risk of lifetime IPV perpetration across a range of mental disorders [5, 53]; it is not yet clear whether this is also the case for past-year IPV perpetration. When analysing associations between depression and IPV, we were only able to consider associations with physical IPV perpetration and could not investigate the perpetration of psychological and sexual IPV, or of coercive and controlling behaviours. We were also unable to analyse, due to a lack of data, whether IPV victimisation mediated the relationship between depression and past-year physical IPV perpetration among men. Third, heterogeneity in the measurement of potential mediators (e.g., alcohol misuse) meant that data harmonisation was challenging; heterogeneity for each analysis may be attributable to inadequate harmonisation. Several potential mediators of interest were excluded from the analysis, because they could not be adequately harmonised or because they were not consistently available across datasets. Fourth, due to insufficient data across datasets, we could not control for a number of key confounders, such as exposure to IPV during childhood or childhood experiences of abuse, which we know are associated with both mental disorders and IPV perpetration [14, 26, 28]. This may have resulted in an over-estimate of the true effect of depression on past-year IPV perpetration. Finally, data were drawn from high-income countries only to minimise heterogeneity, and thus, findings cannot be generalised to low- and middle-income country settings. Future research should consider replicating the methodology of this study using datasets from low- and middle-income countries.

### Implications

Mental health services, criminal justice services, and domestic violence perpetrator programmes should be aware that, although most people with mental disorders are not violent, depression is associated with an increased risk of IPV perpetration. Longitudinal research is clearly needed to explore directions of causality, and underlying mechanisms, for men and women, which may differ by sex. To our knowledge, there has also been no research examining whether treatment of depression (e.g., by antidepressants) impacts on the risk of IPV perpetration.

Evidence is lacking on the effectiveness of perpetrator programmes for perpetrators with mental disorders [2, 54] and future research should address this gap as a matter of priority. If effective, these programmes may not only reduce IPV perpetration, but also depression among those who perpetrate IPV and their victims. In clinical settings, professionals working with perpetrators should consider whether there are potentially modifiable risk factors for IPV perpetration that could be addressed. Relevant responses may include psychological therapies for emotional regulation and/or treatment with antidepressants or mood stabilisers; the latter is known to be associated with reduced risk of violent crime [55], but to our knowledge, this has not been investigated in relation to IPV perpetration. Although alcohol misuse was not found to mediate the relationship between depression and IPV perpetration, it is a known risk factor for IPV perpetration that should also be considered an intervention target [56].

Mental health and other professionals working with women with mental disorders who report or are known to be perpetrators of IPV should be aware of the high prevalence of IPV victimisation among this group. Barriers to disclosure of IPV victimisation, and other forms of trauma, should be considered [57] and where elicited, safety of both partners prioritised. Therefore, there may be multiple risks that need to be assessed and considered when managing care. Findings also raise the possibility that interventions aimed at reducing risk of IPV victimisation in women might be helpful in preventing IPV perpetration. Evidence suggests that advocacy (empowerment, safety information, and referrals) interventions, such as those typically provided by specialist violence against women and girls services, can help men and women in terms of both safety and recovery [58, 59].

Our review identified very few studies with the data we needed to investigate associations between mental disorder and IPV perpetration. This study has also highlighted the need to strengthen collection of these data within population-based surveys, and for greater consistency of data collection across these surveys. At a minimum, the collection of data on violence should include physical, sexual, and psychological violence, and, for each type of violence, the number of repetitions in the past year, the sex of the perpetrator and of the victim, and the relationship between the perpetrator and the victim [60]. Studies should additionally collect data on a range of mental disorders, particularly anxiety disorders, personality disorders, and substance abuse disorders, which have been shown elsewhere to be associated with increased risk of IPV perpetration [3, 5], and key covariates, particularly substance misuse, childhood trauma (including physical abuse, sexual abuse, witnessing IPV, and neglect), and IPV victimisation in adulthood.

### Supplementary Information

Below is the link to the electronic supplementary material.

Supplementary file1 (PDF 233 KB)

Supplementary file2 (PDF 4237 KB)

Supplementary file3 (PDF 377 KB)

## Data Availability

The National Longitudinal Study of Adolescent to Adult Health and the National Survey of Families and Households datasets are publicly available to download. For access to the 2014 Adult Psychiatric Morbidity Survey, Christchurch Health and Development Study, and Environmental Risk (E-Risk) Longitudinal Twin Study, please contact the study principal investigator.

## Code availability

The code has been provided as supplementary information.
