# Supplementary material for: Past-year intimate partner violence perpetration among people with and without depression: an individual participant data (IPD) meta-mediation analysis
Source: Soc Psychiatry Psychiatr Epidemiol. 2021 Nov 29;58(12):1735–47. doi: 10.1007/s00127-021-02183-w (PMC10627935; doi:10.1007/s00127-021-02183-w)
Supplement: Supplementary file 1 — Supplementary file1 (PDF 233 KB) [file 127_2021_2183_MOESM1_ESM.pdf]

Article title:

Past year intimate partner violence perpetration among people with depression: an individual participant data (IPD) meta-mediation analysis.

Journal name:

Social Psychiatry and Psychiatric Epidemiology.

Author names:

Katherine Saunders[1], Sabine Landau[1,2], Louise M Howard[1], Helen L. Fisher[3,4], Louise Arseneault[3,4], Geraldine F H McLeod[5], Sian Oram[1]

Affiliation:

[1] King's College London, London, Health Service and Population Research Department, Institute of Psychiatry, Psychology & Neuroscience, London, United Kingdom

[2] King's College London, London, Biostatistics and Health Informatics Department, Institute of Psychiatry, Psychology & Neuroscience, London, United Kingdom

[3] King's College London, London, Social, Genetic & Developmental Psychiatry Centre, Institute of Psychiatry, Psychology & Neuroscience, London, United Kingdom

[4] ESRC Centre for Society and Mental Health, King's College London, London, United Kingdom

[5] Department of Psychological Medicine, University of Otago Christchurch, New Zealand

E-mail address of the corresponding author:

sian.oram@kcl.ac.uk

Depression	Original variable name and description	Original output format	Code	New variable
**Environmental Risk (E-Risk) Longitudinal Twin Study**	Mdepm10 – past year depression among mothers, measured using the DIS	Binary	rename mdepm10 DepressionPY	DepressionPY
**National Survey of Families and Households**	On how many days during the past week did the participant:	0	recode E202A 0=0 1/2=1 3/4=2 5/7=3 8/max=.	CESDbinary
	E202A feel bothered by things that usually don't bother you?	1	tab E202A	
	E202B Not feel like eating; your appetite was poor?	2	recode E202B 0=0 1/2=1 3/4=2 5/7=3 8/max=.	
	E202C Feel that you could not shake off the blues even with help from your family or friends?	3	tab E202B	
	E202D Have trouble keeping your mind on what you were doing?	4	recode E202C 0=0 1/2=1 3/4=2 5/7=3 8/max=.	
	E202E Feel depressed?	5	tab E202C	
	E202F Feel that everything you did was an effort?	6	recode E202D 0=0 1/2=1 3/4=2 5/7=3 8/max=.	
	E202G Feel fearful?	7	tab E202D	
	E202H Sleep restlessly?	8-Don't know	recode E202E 0=0 1/2=1 3/4=2 5/7=3 8/max=.	
	E202I Talk less than usual?	9-No answer	tab E202E	
	E202J Feel lonely?		recode E202F 0=0 1/2=1 3/4=2 5/7=3 8/max=.	
	E202K Feel sad?		tab E202F	
	E202L Feel you could not get going?		recode E202G 0=0 1/2=1 3/4=2 5/7=3 8/max=.	
			tab E202G	
			recode E202H 0=0 1/2=1 3/4=2 5/7=3 8/max=.	
			tab E202H	
			recode E202I 0=0 1/2=1 3/4=2 5/7=3 8/max=.	
			tab E202I	
			recode E202J 0=0 1/2=1 3/4=2 5/7=3 8/max=.	
			tab E202J	
			recode E202K 0=0 1/2=1 3/4=2 5/7=3 8/max=.	
			tab E202K	
			recode E202L 0=0 1/2=1 3/4=2 5/7=3 8/max=.	
			tab E202L	

			gen CESDadapt= E202A+ E202B+ E202C+ E202D+ E202E+ E202F+ E202G+ E202H+ E202I+ E202J+ E202K+ E202L<br>tab CESDadapt<br><br>gen CESDbinary=CESDadapt<br>recode CESDbinary min/11=0 12/max=1<br>label variable CESDbinary "Depression score of 12+ on adapted CESD"<br>label define CESDbinary 0 "No" 1 "Yes"<br>label values CESDbinary CESDbinary<br>tab CESDadapt CESDbinary	
**National Longitudinal Study of Adolescent to Adult Health**	H4MH18 - You were bothered by things that usually don't bother you.<br>H4MH19 - (During the past seven days:) You could not shake off the blues, even with help from your family and your friends.<br><br>H4MH20 - (During the past seven days:) You felt you were just as good as other people.<br><br>H4MH21 - (During the past seven days:) You had trouble keeping your mind on what you were doing.<br>H4MH22 - (During the past seven days:) You felt depressed.<br>H4MH23 - (During the past seven days:) You felt that you were too tired to do things.<br>H4MH24 - (During the past seven days:) You felt happy.<br>H4MH25 - (During the past seven days:) You enjoyed life.<br>H4MH26 - (During the past seven days:) You felt sad.	0 = Never or rarely, 1 = Sometimes, 2 = A lot of the time, 3 = Most of the time (and reverse scores for 3 variables)	gen H4MH24reverse = H4MH24<br><br>recode H4MH24reverse 0=3 1=2 2=1 3=0 4/max=.<br><br>label define H4MH24reverse 3 "Never or rarely" 2 "Sometimes" 1 "A lot of the time" 0 "Most of the time or all of the time" 4 "Dont know, refused"<br>label values H4MH24reverse H4MH24reverse<br><br>tab H4MH24reverse H4MH24<br><br>gen H4MH25reverse = H4MH25<br>recode H4MH25reverse 0=3 1=2 2=1 3=0 4/max=.<br>label define H4MH25reverse 3 "Never or rarely" 2 "Sometimes" 1 "A lot of the time" 0 "Most of the time or all of the time" 4 "Dont know, refused"	CESDbinary

	H4MH27 - (During the past seven days:) You felt that people disliked you, during the past seven days.		label values H4MH25reverse H4MH25reverse<br><br>tab H4MH25reverse H4MH25<br><br>gen H4MH20reverse = H4MH20<br>recode H4MH20reverse 0=3 1=2 2=1 3=0 4/max=.<br>label define H4MH20reverse 3 "Never or rarely" 2 "Sometimes" 1 "A lot of the time" 0 "Most of the time or all of the time" 4 "Dont know, refused"<br>label values H4MH20reverse H4MH20reverse<br>tab H4MH20reverse H4MH20<br><br>tab H4MH20reverse<br>tab H4MH25reverse<br>tab H4MH24reverse<br>tab H4MH18<br>tab H4MH19<br>tab H4MH21<br>tab H4MH22<br>tab H4MH23<br>tab H4MH26<br>tab H4MH27<br><br>list AID if H4MH20reverse > 3<br><br>list AID if H4MH25reverse > 3<br><br>list AID if H4MH24reverse > 3<br><br>list AID if H4MH18 > 3<br>list AID if H4MH19 > 3<br>list AID if H4MH21 > 3<br>list AID if H4MH22 > 3<br>list AID if H4MH23 > 3<br>list AID if H4MH26 > 3<br>list AID if H4MH27 > 3	

			There are 8 participants who have one missing variable, so can be included in the depression analyses, ppt 2096 refused to answer all CES-D questions/<br><br>recode H4MH18 4/max=.<br>recode H4MH19 4/max=.<br>recode H4MH21 4/max=.<br>recode H4MH22 4/max=.<br>recode H4MH23 4/max=.<br>recode H4MH26 4/max=.<br>recode H4MH27 4/max=.<br><br>gen CESD = H4MH20reverse + H4MH25reverse + H4MH24reverse + H4MH18 + H4MH19 + H4MH21 + H4MH22 + H4MH23 + H4MH26 + H4MH27<br><br>/* STATA has given all participants with any missing values a combined score of 'missing'.<br>For the 8 participants who have only one missing score, sum their scores and enter them into their CESD cell BEFORE NEXT STEP<br>ppt 474 - 4,<br>ppt 771 - 7,<br>ppt 1668 - 8,<br>ppt 2154 - 5,<br>ppt 2947 - 12,<br>ppt 2993 - 3,<br>ppt 3332 - 6,<br>ppt 3639 - 11 */<br><br>replace CESD = 4 in 474<br>replace CESD = 7 in 771<br>replace CESD = 8 in 1668<br>replace CESD = 5 in 2154<br>replace CESD = 12 in 2947<br>replace CESD = 3 in 2993<br>replace CESD = 6 in 3332<br>replace CESD = 11 in 3639<br><br>gen CESDbinary = CESD	

			recode CESDbinary min/7=0 8/max=1<br>label variable CESDbinary "Positive screen on CES-D 10 scale (binary)"<br>label define CESDbinary 0 "No" 1 "Yes"<br>label values CESDbinary CESDbinary<br>tab CESDbinary CESD	
**Christchurch Health and Development Study**	D30mdy – DSM IV major depression in the past year	Binary	rename d30mdy majordepPY<br>label variable majordepPY "Major depression in the past year"<br>label define majordepPY 0 "No" 1 "Yes"<br>label values majordepPY majordepPY	majordepPY
**2014 Adult Psychiatric Morbidity Survey**	milddep - CISR: (D) mild depression (0 'not present' 1 'present'.)<br>moddep - CISR: (D) moderate depression (0 'not present' 1 'present'.)<br>sevdep - CISR: (D) Severe depression - ICD-10 diagnosis f32.2	Binary	gen anydep=.<br><br>replace anydep =1 if milddep==1 | moddep==1 | sevdep==1<br>replace anydep=0 if milddep==0 & moddep==0 & sevdep==0<br>label variable anydep "any depression diagnosis"<br>label define anydep 0 "no" 1 "yes"<br>label values anydep anydep<br>tab anydep milddep<br>tab anydep moddep<br>tab anydep sevdep	anydep

Past year physical IPV perpetration	Original variable name and description	Original output format	Code	New variable
**Environmental Risk (E-Risk) Longitudinal Twin Study**	Vi2mm10 – mum's violence perpetration in past year<br>Composite variables include:<br>RP7m10 Have you pushed, grabbed, or shoved a partner?<br>RP9m10 Have you slapped a partner?<br>RP11m10 Have you shaken a partner?<br>RP13m10 Have you thrown an object at a partner that could hurt them?<br>RP15m10 Have you kicked, bitten, or hit a partner with a fist?<br>RP17m10 Have you hit or tried to hit a partner with something?<br>RP19m10 Have you physically twisted a partner's arm?<br>RP21m10 Have you thrown or tried to throw a partner bodily?<br>RP23m10 Have you beaten a partner up (multiple blows)?<br>RP25m10 Have you choked or strangled a partner?<br>RP27m10 Have you threatened a partner with a knife or gun?<br>RP29m10 Have you used a knife or gun on a partner?	Number of 'sometimes' and 'yes' responses to composite questions	gen PhysicalIPVperpPY = vi2mm10<br>recode PhysicalIPVperpPY 0=0 1/max=1<br>label variable PhysicalIPVperpPY "Mother's past year perpetration of physical IPV"<br>label define PhysicalIPVperpPY 1 "Yes" 0 "No"<br>label values PhysicalIPVperpPY PhysicalIPVperpPY<br>tab PhysicalIPVperpPY vi2mm10	PhysicalIPVperpPY
**National Survey of Families and Households**	Asked of those cohabiting:<br>E613 During the past year, how many fights with your partner resulted in YOU hitting, shoving, or throwing things at him/her?<br>E616 Has your PARTNER been cut, bruised, or seriously injured in a fight with you?<br>Asked of married participants:<br>E709 During the past year, how many fights with your husband/wife resulted in YOU hitting, shoving, or throwing things at him/her?<br>E712 Has your HUSBAND/WIFE been cut, bruised, or seriously injured in a fight with you?	E613 and E709 – number of fights<br>E616 and E712 - binary	gen physIPVperp1=E613<br>recode physIPVperp1 0=0 1/4=1 5/max=.<br>label variable physIPVperp1 "Had a physical fight in the past year which has resulted in the respondent hitting, shoving, or throwing things at their partner"<br>label define physIPVperp1 0 "No" 1 "Yes"<br>label values physIPVperp1 physIPVperp1<br>tab physIPVperp1 E613<br>gen physIPVperp2=E616<br>recode physIPVperp2 1=1 2=0 3/max=.	PhysicalIPVperpPY

			label variable physIPVperp2 "Partner been cut, brusied or seriously injured in a fight with the respondent"<br>label define physIPVperp2 0 "No" 1 "Yes"<br>label values physIPVperp2 physIPVperp2<br>tab physIPVperp2 E616<br><br>gen physIPVperp3=E709<br>recode physIPVperp3 0=0 1/4=1 6/max=.<br>label variable physIPVperp3 "Had a physical fight in the past year which has resulted in the respondent hitting, shoving, or throwing things at their partner"<br>label define physIPVperp3 0 "No" 1 "Yes"<br>label values physIPVperp3 physIPVperp3<br>tab physIPVperp3 E709<br><br>gen physIPVperp4=E712<br>recode physIPVperp4 1=1 2=0 3/max=.<br>label variable physIPVperp4 "Partner been cut, brusied or seriously injured in a fight with the respondent"<br>label define physIPVperp4 0 "No" 1 "Yes"<br>label values physIPVperp4 physIPVperp4<br>tab physIPVperp4 E712<br><br>gen PhysicalIPVperpPY =.<br>replace PhysicalIPVperpPY =0 if E708==2<br>replace PhysicalIPVperpPY =0 if E612==2<br>replace PhysicalIPVperpPY =1 if physIPVperp1==1 | physIPVperp2==1 | physIPVperp3==1 | physIPVperp4==1<br>label variable PhysicalIPVperpPY "Any physical or injurious IPV perpetration in the past year"<br>label define PhysicalIPVperpPY 0 "No" 1 "Yes"<br>label values PhysicalIPVperpPY PhysicalIPVperpPY<br>tab PhysicalIPVperpPY		

**National Longitudinal Study of Adolescent to Adult Health**	H4RD22 - How often (have/did) you threatened {initials} with violence, pushed or shoved (him/her), or thrown something at (him/her) that could hurt?<br><br>H4RD23 - How often (have/did) you (slapped/slap), hit, or (kicked/kick) {initials}?	Frequency of each behaviour in the past year	gen PhyIPVperpPY1 = H4RD22<br><br>recode PhyIPVperpPY1 0/1=0 2/7=1 8/max=.<br><br>label variable PhyIPVperpPY1 "Perpetration of pushing, shoving, throwing in PY"<br>label define PhyIPVperpPY1 0 "No" 1 "Yes" 2 "Dont know, refused"<br><br>label values PhyIPVperpPY1 PhyIPVperpPY1<br>tab PhyIPVperpPY1 H4RD22<br><br>gen PhyIPVperpPY2 = H4RD23<br>recode PhyIPVperpPY2 0/1=0 2/7=1 8/max=.<br>label variable PhyIPVperpPY2 "Perpetration of slapping, hitting, kicking in PY"<br>label define PhyIPVperpPY2 0 "No" 1 "Yes" 2 "Dont know, refused"<br><br>label values PhyIPVperpPY2 PhyIPVperpPY2<br>tab PhyIPVperpPY2 H4RD23<br><br>gen PhysicalIPVperpPY=.<br>replace PhysicalIPVperpPY = 1 if PhyIPVperpPY2 == 1<br>replace PhysicalIPVperpPY = 1 if PhyIPVperpPY1 == 1<br>replace PhysicalIPVperpPY = 0 if PhyIPVperpPY2 == 0 & PhyIPVperpPY1 == 0<br>label variable PhysicalIPVperpPY "Perpetration of physical IPV perpetration in PY"<br>label define PhysicalIPVperpPY 0 "No" 1 "Yes"<br>label values PhysicalIPVperpPY PhysicalIPVperpPY<br>tab PhysicalIPVperpPY<br>tab PhysicalIPVperpPY PhyIPVperpPY2<br>tab PhysicalIPVperpPY PhyIPVperpPY1	PhysicalIPVperpPY
**Christchurch Health and Development Study**	label z1531='IPV perpetration grabbed or shook partner';<br>label z1532='IPV perpetration threw or tried to throw your partner bodily';<br><br>label z1533='IPV perpetration threw an object at partner';	Individual frequency counts for each behaviour	gen perp1=z1531<br><br>recode perp1 0=0 1/7=1 8/max=2<br><br>gen perp2=z1532	PhysicalIPVperpPY

	label z1534='IPV perpetration choked or strangled partner';		recode perp2 0=0 1/7=1 8/max=2	
	label z1535='IPV perpetration kicked your partner';		gen perp3=z1533	
	label z1536='IPV perpetration became abusive after using drugs or alcohol';		recode perp3 0=0 1/7=1 8/max=2	
	label z1537='IPV perpetration punched or hit your partner with something';		gen perp4=z1534	
	label z1538='IPV perpetration slammed partner into a wall';		recode perp4 0=0 1/7=1 8/max=2	
	label z1539='IPV perpetration burned or scaled partner on purpose';		gen perp5=z1535	
	label z1540='IPV perpetration beat partner up';		recode perp5 0=0 1/7=1 8/max=2	
	label z1541='IPV perpetration threatened partner with knife or gun';		gen perp6=z1536	
	label z1542='IPV perpetration used knife or gun on partner';		recode perp6 0=0 1/7=1 8/max=2	
	label z1525='IPV perpetration threatened to hit or throw something at partner';		gen perp7=z1537	
	label z1526='IPV perpetration physically twisted partners arm or hair';		recode perp7 0=0 1/7=1 8/max=2	
	label z1527='IPV perpetration pushed or shoved partner';		gen perp8=z1538	
	label z1528='IPV perpetration slapped partner';		recode perp8 0=0 1/7=1 8/max=2	
			gen perp9=z1539<br>recode perp9 0=0 1/7=1 8/max=2<br>gen perp10=z1540<br>recode perp10 0=0 1/7=1 8/max=2<br>gen perp11=z1541<br>recode perp11 0=0 1/7=1 8/max=2<br>gen perp12=z1542<br>recode perp12 0=0 1/7=1 8/max=2<br>gen perp13=z1526<br>recode perp13 0=0 1/7=1 8/max=2<br>gen perp14=z1527<br>recode perp14 0=0 1/7=1 8/max=2<br>gen perp15=z1528<br>recode perp15 0=0 1/7=1 8/max=2<br>gen PhysicalIPVperpPY =.<br>replace PhysicalIPVperpPY = 1 if perp1 == 1 | perp2 == 1 | perp3 == 1 | perp4 == 1 | perp5 == 1 | perp6 == 1 | perp7 == 1 | perp8 == 1 | perp9 == 1 | perp10 == 1 | perp11 == 1 | perp12 == 1 | perp13 == 1 | perp14 == 1 | perp15 == 1	

			replace PhysicalIPVperpPY = 0 if perp1 == 0 & perp2 == 0 & perp3 == 0 & perp4 == 0 & perp5 == 0 & perp6 == 0 & perp7 == 0 & perp8 == 0 & perp9 == 0 & perp10 == 0 & perp11 == 0 & perp12 == 0 & perp13 == 0 & perp14 == 0 & perp15 == 0<br><br>label variable PhysicalIPVperpPY "Perpetration of any physical IPV in the past year"<br>label define PhysicalIPVperpPY 0 "No" 1 "Yes"<br>label values PhysicalIPVperpPY PhysicalIPVperpPY	
**2014 Adult Psychiatric Morbidity Survey**	Act2Whn Variable label = IPV: Perpetration - Pushed, held, pinned down, slapped a partner/ex in the past year. Act3Whn Variable label = IPV: Perpetration - Kicked, bit, hit, or thrown something at a partner/ex-partner that hurt them in past year	Value = 1.0 Label = Yes<br>Value = 2.0 Label = No<br>Value = -8.0 Label = Don't know<br>Value = -9.0 Label = Refused<br>Value = 8.0 Label = Not on route<br>Value = 9.0 Label = Don t Understand/Does Not Apply<br>Value = -1.0 Label = Not applicable	gen PhysicalIPVperpPY=.<br>replace PhysicalIPVperpPY = 1 if Act2Whn== 1 | Act3Whn==1<br>replace PhysicalIPVperpPY = 0 if Act2Whn== 2 & Act3Whn==2<br>replace PhysicalIPVperpPY = 0 if Act2Whn==-1 & Act3Whn==-1<br>label variable PhysicalIPVperpPY "Physical IPV perpetration in the past year"<br>label define PhysicalIPVperpPY 0 "No" 1 "Yes"<br>label values PhysicalIPVperpPY<br>tab PhysicalIPVperpPY<br>tab PhysicalIPVperpPY Act2Whn<br>tab PhysicalIPVperpPY Act3Whn	PhysicalIPVperpPY

Severe past year IPV perpetration	Original variable name and description	Original output format	Code
**Environmental Risk (E-Risk) Longitudinal Twin Study**	RP15m10 Have you kicked, bitten, or hit a partner with a fist?<br><br>RP17m10 Have you hit or tried to hit a partner with something?<br><br>RP19m10 Have you physically twisted a partner's arm?<br><br>RP21m10 Have you thrown or tried to throw a partner bodily?<br><br>RP23m10 Have you beaten a partner up (multiple blows)?<br><br>RP25m10 Have you choked or strangled a partner?<br><br>RP27m10 Have you threatened a partner with a knife or gun?<br><br>RP29m10 Have you used a knife or gun on a partner?	Frequency count of acts of perpetration in the past year (0-10)	gen sev1=rp15m10<br>recode sev1 0=0 1/2=1 3/max=2 -8=3<br>gen sev2=rp17m10<br>recode sev2 0=0 1/2=1 3/max=2 -8=3<br>gen sev3=rp19m10<br>recode sev3 0=0 1/2=1 3/max=2 -8=3<br>gen sev4=rp21m10<br>recode sev4 0=0 1/2=1 3/max=2 -8=3<br>gen sev5=rp23m10<br>recode sev5 0=0 1/2=1 3/max=2 -8=3<br>gen sev6=rp25m10<br>recode sev6 0=0 1/2=1 3/max=2 -8=3<br>gen sev7=rp27m10<br>recode sev7 0=0 1/2=1 3/max=2 -8=3<br>gen sev8=rp29m10<br>recode sev8 0=0 1/2=1 3/max=2 -8=3<br><br>gen SevPhysIPVPerp=.<br>replace SevPhysIPVPerp=1 if sev1==1 | sev2==1 | sev3==1 | sev4==1 | sev5==1 | sev6==1 | sev7==1 | sev8==1<br>replace SevPhysIPVPerp=0 if sev1==0 & sev2==0 & sev3==0 & sev4==0 & sev5==0 & sev6==0 & sev7==0 & sev8==0<br>label variable SevPhysIPVPerp "Perpetration of severe physical IPV in the past year"<br>label define SevPhysIPVPerp 0 "No" 1 "Yes"<br>label values SevPhysIPVPerp SevPhysIPVPerp
**Christchurch Health and Development Study**	label z1534='IPV perpetration choked or strangled partner';<br><br>z1532 'IPV perpetration threw or tried to throw your partner bodily';<br>label z1535='IPV perpetration kicked your partner';<br>label z1537='IPV perpetration punched or hit your partner with something';<br>label z1538='IPV perpetration slammed partner into a wall';<br>label z1539='IPV perpetration burned or scaled partner on purpose';<br>label z1540='IPV perpetration beat partner up';<br>label z1541='IPV perpetration threatened partner with knife or gun';	Individual frequency counts for each behaviour	gen SevPhysIPVPerp = .<br>replace SevPhysIPVPerp = 1 if perp2==1 | perp4 == 1 | perp5 == 1 | perp7 == 1 | perp8 == 1 | perp9 == 1 | perp10==1 | perp11 == 1 | perp12 == 1<br>replace SevPhysIPVPerp = 0 if perp2==0 & perp4 == 0 & perp5 == 0 & perp7 == 0 & perp8 == 0 & perp9 == 0 & perp10==0 & perp11 == 0 & perp12 == 0<br>label variable SevPhysIPVPerp "Perpetration of severe physical IPV in the past year"<br>label define SevPhysIPVPerp 0 "No" 1 "Yes"<br>label values SevPhysIPVPerp SevPhysIPVPerp<br>tab SevPhysIPVPerp<br>tab SevPhysIPVPerp z1532

	label z1542='IPV perpetration used knife or gun on partner';		tab SevPhysIPVPerp z1534<br>tab SevPhysIPVPerp z1535<br>tab SevPhysIPVPerp z1537<br>tab SevPhysIPVPerp z1538<br>tab SevPhysIPVPerp z1539<br>tab SevPhysIPVPerp z1540<br>tab SevPhysIPVPerp z1541<br>tab SevPhysIPVPerp z1542
**National Longitudinal Study of Adolescent to Adult Health**	H4RD23 - How often (have/did) you (slapped/slap), hit, or (kicked/kick) {initials}?	frequency count of each behaviour in the past year	gen SevPhysIPVPerp = PhysicalIPVperpPY<br>replace SevPhysIPVPerp = 3 if PhyIPVperpPY2==1<br>tab SevPhysIPVPerp<br>recode SevPhysIPVPerp 0/1=0 3=1<br>label variable SevPhysIPVPerp "Perpetration of severe physical IPV in the past year"<br>label define SevPhysIPVPerp 0 "No" 1 "Yes" 2 "Dont know, refused"<br>label values SevPhysIPVPerp SevPhysIPVPerp<br>tab SevPhysIPVPerp
**APMS**	Act3Whn Variable label = IPV: Perpetration - Kicked, bit, hit, or thrown something at a partner/ex-partner that hurt them in past year	Value = 1.0<br>Label = Yes<br>Value = 2.0<br>Label = No<br>Value = -8.0<br>Label = Don't know<br>Value = -9.0<br>Label = Refused<br>Value = 8.0<br>Label = Not on route<br>Value = 9.0<br>Label = Don t Understand/Does Not Apply<br>Value = -1.0<br>Label = Not applicable	gen SevPhysIPVPerp=.<br>replace SevPhysIPVPerp = 1 if Act3Whn==1<br>replace SevPhysIPVPerp = 0 if Act3Whn==2<br>label variable SevPhysIPVPerp "Severe physical IPV perpetration in the past year"<br>label define SevPhysIPVPerp 0 "No" 1 "Yes"<br>label values SevPhysIPVPerp SevPhysIPVPerp<br>tab SevPhysIPVPerp<br>tab SevPhysIPVPerp Act3Whn
**National survey of families and households**	Asked of those who were cohabiting:<br>E616 Has your PARTNER been cut, bruised, or seriously injured in a fight with you?<br><br>Asked of married people:<br><br>E712 Has your HUSBAND/WIFE been cut, bruised, or seriously injured in a fight with you?	0-None<br>1-One<br>2-Two<br>3-Three fights<br>4-Four or more fights<br>6-Inapplicable<br>7-Refused<br>9-No answer<br>1-Yes<br>2-No<br>6-Inapplicable<br>7-Refused<br>9-No answer	gen SevPhysIPVPerp=PhysicalIPVperpPY<br>replace SevPhysIPVPerp=3 if physIPVperp2==1 | physIPVperp4==1<br>recode SevPhysIPVPerp 0/1=0 3=1<br>label variable SevPhysIPVPerp "Any severe IPV perpetration in the past year"<br>label define SevPhysIPVPerp 0 "No" 1 "Yes"<br>label values SevPhysIPVPerp SevPhysIPVPerp<br>tab SevPhysIPVPerp

**New variable name**
SevPhysIPVPerp
SevPhysIPVPerp

SevPhysIPV Perp
SevPhysIPV Perp
SevPhysIPV Perp

Sex	Original variable name and description	Original output format	Code	New variable name
**Environmental Risk (E-Risk) Longitudinal Twin Study**	N/A	N/A	N/A	N/A
**Christchurch Health and Development Study**	gender - sex of participant	binary - male	label define gender 1 "Male" 2 "Female" label values gender gender rename gender sexgender	sexgender
**National Longitudinal Study of**	BIO_SEX4 - sex of participant	binary - male	rename BIO_SEX4 sexgender	sexgender
**APMS**	ResSex	Binary - male	None	None
**National survey of families and households**	M2DP01 - sex of participant	Binary - male or female	rename M2DP01 sexgender	sexgender

Age	Original variable name and description	Original output format	Code	New variable name
**Environmental Risk (E-Risk)**	mage10 - mothers age at time of data collection	raw age	gen age = mage10	age
**Christchurch Health and Development Study**	N/A	N/A	N/A	N/A
**National Longitudinal Study of Adolescent to Adult Health**	IYEAR4 and H5OD1Y - survey year and birth year	survey year and birth yea	gen age1=IYEAR4-H4OD1Y tab age1	age1
**APMS**	DVResAge95	raw age	N/A	N/A
**National survey of families and households**	M2BP01	raw age	gen age=M2BP01	age

Education	Original variable name and description	Original output format	Code	New variable name
**Environmental Risk (E-Risk) Longitudinal Twin Study**	hiedgm57 - highest education qual 3 years ago, hiedm5 - education at child age 5, hiedm7 - education at child age 7	None<br>1+ CSE/GCSE/NVQ1<br>5+ CSE/GCSE/NVQ2<br>A Lev & Higher	gen education=hiedm5<br>replace education=hiedm7 if hiedm7>hiedm5<br>replace education=hiedm5 if hiedm7==.<br>recode education -9=. -1=. 0=0 1/3=1 4/7=2<br>label define education 0 "None" 1 "High school qualifications" 2 "Degree/equiv or higher"<br>label values education education<br>label variable education "Highest education qualification at child age 7"<br>tab education hiedgm57<br>tab education, gen (educationdummy)	educationdummy
**Christchurch Health and Development Study**	hieduc30 - highest education qualification by age 30	Discrete categories for each education qualification 1 "None" 2 "High school qualifications" 3 "Trade/technical qualifications" 4 "Tertiary (University/Polytech Degree) [illegible]	gen education=hieduc30<br>recode education 1=0 2=1 3/4=2<br>label variable education "Highest education qualification"<br>label define education 0 "None" 1 "High school qualifications/equivalent" 2 "Degree/equiv"<br>label values education education<br>tab hieduc30 education<br>tab education, gen [illegible]	educationdummy

**National Longitudinal Study of Adolescent to Adult Health**	H4ED2 - highest education qual	(1) 8th grade or less 16 0.3 %<br>2 (2) Some high school 383 7.5 %<br>3 (3) High school graduate 835 16.3 %<br>4 (4) Some vocational/technical training (after high school) 182 3.6 %<br>5 (5) Completed vocational/technical training (after high scho 327 6.4 %<br>6 (6) Some college 1702 33.3 %<br>7 (7) Completed college (bachelor's degree) 1012 19.8 %<br>8 (8) Some graduate school 199 3.9 %<br>9 (9) Completed a master's degree 256 5.0 %<br>10 (10) Some graduate training beyond a master's	gen education=H4ED2<br>recode education 1/2=0 3/4=1 5=2 6=1 7/13=2 98=.<br>label variable education "Highest education qualification"<br>label define education 0 "No qualifications" 1 "High school / equivalent" 2 "Degree/equiv"<br>label values education education<br>tab H4ED2 education<br>tab education, gen(educationdummy)	educationdummy
**APMS**	edqual5 - highest edu qual	"-1 "Item not applicable", 1 "Degree", 2 "Teaching, HND, nursing", 3 "A Level", 4 "GCSE or equivalent", 5 "Foreign/other", 6 "No qualifications", -8 "Don't know", -9 "Refused	None	None

**National survey of families and households**	COMPLED - This variable is an extraction of primary respondents' formal educational attainment level from their detailed education attendance and degree history (M507 to M521). The variable represents either the highest elementary or secondary grade level completed, or the highest postsecondary degree level obtained from a college, university, or professional school. Passing a high school equivalency test (GED) is counted as high school graduation. The variable does not include attendance at or certificates received from vocational, technical, trade, business, secretarial, or nursing schools. Persons who did NOT complete high school but attended a college (usually a two-year community or junior college for one year or less) without receiving a degree or a GED are coded at the grade level they left elementary or high school.	00 - no formal education<br>01 - first grade<br>02 - second grade<br>03 - third grade<br>04 - fourth grade<br>05 - fifth grade<br>06 - sixth grade<br>07 - seventh grade<br>08 - eighth grade<br>09 - ninth grade<br>10 - tenth grade<br>11 - eleventh grade<br>12 - high school graduate<br>13 - attended a two- or four-year college or university or a professional school for various periods of time, but did not receive a degree.<br>14 - Associate Degree (2-year)<br>16 - Bachelor's Degree<br>18 - Master's Degree	gen education=COMPLED<br>recode education 0/9=0 10/13=1 14/20=2 99=.<br>label variable education "Highest education qualification"<br>label define education 0 "None" 1 "High school qualifications" 2 "University degree or equivalent (and higher)"<br>label values education education<br>tab COMPLED education<br>tab education, gen (educationdummy)	educationdummy

Income	Original variable name and description	Original output format	Code	New variable name
**Environmental Risk (E-Risk) Longitudinal Twin Study**	ed56m5 - income in bands	income in bands	tab ed56m5<br>gen income=ed56m5<br>recode income 99=. 0=1999.5 1=4999.5 2=6999.5 3=8999.5 4=10999.5 5=13499.5 6=16499.5 7=18999.5 8=21499.5 9=24499.5 10=27499.5 11=27499.5 12=33499.5 13=36499.5 14=39499.5 15=42499.5<br>label variable income "Approximate income"	income
**Christchurch Health and Development Study**	ppptotfaminc30 - gross total family income in past 12 months	raw income	gen income=ppptotfaminc30	income
**National Longitudinal Study of Adolescent to Adult Health**	H4EC1 - Household income in bands	income in bands	gen income=H4EC1<br>recode income 1=2500 2=7499.5 3=12499.5 4=17499.5 5=22499.5 6=27499.5 7=34999.5 8=44999.5 9=624999.5 10=87499.5 11=124999.5 12=174999.5 96=. 98=.<br>label variable income "Approximate income based on mid point of bands"	
**APMS**	N/A	N/A	N/A	N/A
**National survey of families and households**	IHTOT2 - total household income	raw income	income	income

Number of children	Original variable name and description	Original output format	Code	New variable name
**Environmental Risk (E-Risk) Longitudinal Twin Study**	KidsinFamily10	number of children	None	KidsinFamily 10
**Christchurch Health and Development Study**	z10 - number of children in household		None	z10
**National Longitudinal Study of Adolescent to Adult Health**	H4TR11 - number of children	number of children	gen children=H4TR1 1<br>replace children= . if H4TR9 == 96<br>replace children= . if H4TR9 == 98	children
**APMS**	N/A	N/A	N/A	N/A
**National survey of families and households**	LSTA1NUM<br>Number of children on List A, age 4 or younger.<br>List A includes all children age 18 and younger on household roster with relationship biological child, step-child, adopted child, fost child, or child of lover/partner<br>LSTA2NUM<br>Number of children on List A, age 5-18<br>List A includes all children age 18 and younger on household roster with relationship biological child, step-child	number of children	recode LSTA1NUM 6=.<br>gen Noofchildren= LSTA1NUM+LST A2NUM<br>tab Noofchildren	NoofChildre n

Alcohol use	Original variable name and description	Original output format	Code	New variable name
**Environmental Risk (E-Risk) Longitudinal Twin Study**	alcdxmm10 - Brief Michigan Alcoholism Screening Test (MAST) and adapted based on questions asked in the phase 5 alcohol interview, participants screened positive if displaying 4 or	Binary - 4 or more symptoms = yes, 3 or fewer = no	gen SubMisuse = alcdxmm10	SubMisuse
**Christchurch Health and Development Study**	alcud30 - DSM IV alcohol use disorder past 12 months	Binary	label define alcud30 0 "No" 1 "Yes"<br>label values alcud30 alcud30<br>rename alcud30 AUDPY	AUDPY
**National Longitudinal Study of Adolescent to Adult Health**	H4TO39 During the past 30 days, on how many days did you drink? H4TO40 Think of all the times you have had a drink during the past 30 days. How many drinks did you usually have each time? A 'drink' is a glass of wine, a can or bottle of beer, a wine cooler, a shot glass of liquor, or a mixed drink. (four or five drinks corresponds with a 'binge')	Frequencies	gen bingedrink1 = H4TO39<br>recode bingedrink1 0/3=0 97=0 4/6=1 96=2 98=2<br>label variable bingedrink1 "Number of days on which the participant has had a drink in the past month"<br>label define bingedrink1 0 "Fewer than 2 days a week" 1 "2 or more days a week" 2 "Don't know/refused'"<br>label values bingedrink1 bingedrink1<br>tab bingedrink1 H4TO39<br><br>gen bingedrink2 = H4TO40<br>recode bingedrink2 min/3=0 4/18=1 95=2 96=2 97=0 98=2<br>label variable bingedrink2 "Number of drinks usually consumed on a day of drinking in the last 30 days"<br>label define bingedrink2 0 "Fewer than 3 drinks" 1 "4 or more drinks" 2 "Don't know/refused/not asked"<br>label values bingedrink2 bingedrink2<br>tab bingedrink2 H4TO40<br><br>gen BingeDrink=.<br>replace BingeDrink = 1 if bingedrink1==1 & bingedrink2==1<br>replace BingeDrink = 0 if bingedrink1==0 & bingedrink2==0<br>replace BingeDrink = 0 if bingedrink1==1 & bingedrink2==0<br>replace BingeDrink = 0 if bingedrink1==0 &	BingeDrink
**APMS**	N/A	N/A	N/A	N/A
**National survey of families and households**	Who living here has a problem of drinking too much alcohol? E209A Me	Binary	gen alcoholprob=E209A<br>recode alcoholprob 1=1 2/7=0 8/max=.<br>label variable alcoholprob "Respondent has a problem with drinking too much alcohol"<br>label define alcoholprob 0 "No" 1 "Yes"<br>label values alcoholprob alcoholprob	alcoholprob

Past year IPV victimisation	Original variable name and description	Original output format	Code	New variable name
**Environmental Risk (E-Risk) Longitudinal Twin Study**	viogpm10 - made up of: RP7m10 Have you been pushed, grabbed, or shoved by a partner?<br>RP9m10 Have you been slapped by a partner?<br>RP11m10 Have you been shaken by a partner?<br>RP13m10 Have you had an object thrown at you?<br>RP15m10 Have you been kicked, bitten, or hit by a partner ?<br>RP17m10 Have you been hit or attemped to hit by a partner with something?<br>RP19m10 Have you had you arm physically twisted by a partner's?<br>RP21m10 Have you had your body thrown or tried to throw by a partner?<br>RP23m10 Have you been beaten up by a partner (multiple blows)?<br>RP25m10 Have you been choked or strangled by a partner?<br>RP27m10 Have you been threatened by a partner with a knife or gun?	Number of 'sometimes' and 'yes' responses to composite questions	gen IPVvictPY = viogpm10<br>recode IPVvictPY 0=0 1/max=1<br>label variable IPVvictPY "Mother's past year physical IPV victimisation"<br>label define IPVvictPY 1 "Yes" 0 "No"<br>label values IPVvictPY IPVvictPY<br>tab IPVvictPY viogpm10	IPVvictPY
**Christchurch Health and Development Study**	label z1482='IPV victimisation cursed or swore at you';<br>label z1483='IPV victimisation shouted or yelled at you';<br>label z1484='IPV victimisation partner stomped off during disagreement';<br>label z1485='IPV victimisation deliberately said something to hurt you';<br>label z1486='IPV victimisation called you fat/ugly/unattractive';<br>label z1487='IPV victimisation destroyed something belonging to you';<br>label z1488='IPV victimisation accused you of being a lousy lover';<br>label z1489='IPV victimisation threatened to hit or throw something at you';<br>label z1490='IPV victimisation physically twisted your arm or hair';<br>label z1491='IPV victimisation pushed or shoved you';<br>label z1492='IPV victimisation slapped you';<br>label z1493='IPV victimisation force sex on you';<br>label z1494='IPV victimisation used threats to make you have sex';<br>label z1495='IPV victimisation grabbed or shook you';<br>label z1496='IPV victimisation threw or tried to throw you bodily';<br>label z1497='IPV victimisation threw an	Frequencies for each behaviour	gen vict1=z1482<br>recode vict1 0=0 1/7=1 8/max=2<br>gen vict2=z1483<br>recode vict2 0=0 1/7=1 8/max=2<br>gen vict3=z1484<br>recode vict3 0=0 1/7=1 8/max=2<br>gen vict4=z1485<br>recode vict4 0=0 1/7=1 8/max=2<br>gen vict5=z1486<br>recode vict5 0=0 1/7=1 8/max=2<br>gen vict6=z1487<br>recode vict6 0=0 1/7=1 8/max=2<br>gen vict7=z1488<br>recode vict7 0=0 1/7=1 8/max=2<br>gen vict8=z1489<br>recode vict8 0=0 1/7=1 8/max=2<br>gen vict9=z1490<br>recode vict9 0=0 1/7=1 8/max=2<br>gen vict10=z1491<br>recode vict10 0=0 1/7=1 8/max=2<br>gen vict11=z1492<br>recode vict11 0=0 1/7=1 8/max=2<br>gen vict12=z1493<br>recode vict12 0=0 1/7=1 8/max=2<br>gen vict13=z1494<br>recode vict13 0=0 1/7=1 8/max=2<br>gen vict14=z1495<br>recode vict14 0=0 1/7=1 8/max=2<br>gen vict15=z1496<br>recode vict15 0=0 1/7=1 8/max=2<br>gen vict16=z1497<br>recode vict16 0=0 1/7=1 8/max=2	IPVvictPY

**National Longitudinal Study of Adolescent to Adult Health**	H4RD18 - How often (has/did) {initials} (threatened/threaten) you with violence, (pushed/push) or (shoved/shove) you, or (thrown/throw) something at you that could hurt?<br>H4RD19 - How often (has/did) {initials} (slapped/slap), hit or (kicked/kick) you?<br>H4RD20 - How often (have/did) you (had/have) an injury, such as a sprain, bruise, or cut because of a fight with {initials}?<br>H4RD21 - How often (has/did) {initials} (insisted/insist) on or (made/make) you have sexual relations with (him/her) when you didn't want to?<br>H4RD18 - How often (has/did) {initials} (threatened/threaten) you with violence, (pushed/push) or (shoved/shove) you, or (thrown/throw) something at you that could hurt?<br>H4RD19 - How often (has/did) {initials} (slapped/slap), hit or (kicked/kick) you?<br>H4RD20 - How often (have/did) you (had/have) an injury, such as a sprain, bruise, or cut because of a fight with {initials}?<br>H4RD21 - How often (has/did) {initials} (insisted/insist) on or (made/make) you have sexual relations with (him/her) when you didn't want to?	Frequencies for each behaviour	gen IPVvict4 = H4RD18<br>recode IPVvict4 0/1=0 2/7=1 8/max=.<br>label variable IPVvict4 "IPV victimsation (pushing, shoving, throwing)"<br>label define IPVvict4 0 "No" 1 "Yes" 2 "Dont know, refused"<br>label values IPVvict4 IPVvict4<br>tab IPVvict4 H4RD18<br><br>gen IPVvict5 = H4RD19<br>recode IPVvict5 0/1=0 2/7=1 8/max=.<br>label variable IPVvict5 "IPV victimsation (slapping, kicking, hitting)"<br>label define IPVvict5 0 "No" 1 "Yes" 2 "Dont know, refused"<br>label values IPVvict5 IPVvict5<br>tab IPVvict5 H4RD19<br><br>gen IPVvict6 = H4RD21<br>recode IPVvict6 0/1=0 2/7=1 8/max=.<br>label variable IPVvict6 "IPV victimsation (sexual)"<br>label define IPVvict6 0 "No" 1 "Yes" 2 "Dont know, refused"<br>label values IPVvict6 IPVvict6<br>tab IPVvict6 H4RD21<br><br>gen IPVvictPY = .<br>replace IPVvictPY = 1 if IPVvict4 == 1 | IPVvict5 == 1 | IPVvict6 == 1<br>replace IPVvictPY = 0 if IPVvict4 == 0 &	IPVvictPY
**APMS**	N/A	N/A	N/A	N/A

**National survey of families and households**	E614 During the past year, how many fights with your partner resulted in HIM/HER hitting, shoving, or throwing things at you?<br>E615 Have YOU been cut, bruised, or seriously injured in a fight with your partner?<br>E710 During the past year, how many fights with your husband/wife resulted in HIM/HER hitting, shoving, or throwing things at you?<br>E711 Have YOU been cut, bruised, or seriously injured in a fight with your husband/wife?	Number of fights & binary responses for injury	gen physIPVvict1=E614<br>recode physIPVvict1 0=0 1/4=1 6/max=.<br>label variable physIPVvict1 "Had a physical fight in the past year which has resulted in their partner hitting, shoving, or throwing things at them"<br>label define physIPVvict1 0 "No" 1 "Yes"<br>label values physIPVvict1 physIPVvict1<br>tab physIPVvict1 E614<br><br>gen physIPVvict2=E615<br>recode physIPVvict2 1=1 2=0 3/max=.<br>label variable physIPVvict2 "Been cut, brusied or seriously injured in a fight with your partner"<br>label define physIPVvict2 0 "No" 1 "Yes"<br>label values physIPVvict2 physIPVvict2<br>tab physIPVvict2 E615<br><br>gen physIPVvict3=E710<br>recode physIPVvict3 0=0 1/4=1 6/max=.<br>label variable physIPVvict3 "Had a physical fight in the past year which has resulted in their partner hitting, shoving, or throwing things at them"<br>label define physIPVvict3 0 "No" 1"Yes"<br>label values physIPVvict3 physIPVvict3	IPVvictPY1

Severe past year IPV victimisation	Original variable name and description	Original output format	Code	New variable name
**Environmental Risk (E-Risk) Longitudinal Twin Study**	RP15m10 Have you been kicked, bitten, or hit by a partner ?<br>RP17m10 Have you been hit or attemped to hit by a partner with something?<br>RP19m10 Have you had you arm physically twisted by a partner's?<br>RP21m10 Have you had your body thrown or tried to throw by a partner?<br>RP23m10 Have you been beaten up by a partner (multiple blows)?<br>RP25m10 Have you been choked or strangled by a partner?<br>RP27m10 Have you been threatened by a partner with a knife or gun?<br>RP29m10 Have you had a knife or gun used on you by a partner?	Very true, sometimes true, not true - for each behaviour	gen sev1v=rp16m10<br>recode sev1v 0=0 1/2=1 3/max=2 -8=3<br>gen sev2v=rp18m10<br>recode sev2v 0=0 1/2=1 3/max=2 -8=3<br>gen sev3v=rp20m10<br>recode sev3v 0=0 1/2=1 3/max=2 -8=3<br>gen sev4v=rp22m10<br>recode sev4v 0=0 1/2=1 3/max=2 -8=3<br>gen sev5v=rp24m10<br>recode sev5v 0=0 1/2=1 3/max=2 -8=3<br>gen sev6v=rp26m10<br>recode sev6v 0=0 1/2=1 3/max=2 -8=3<br>gen sev7v=rp28m10<br>recode sev7v 0=0 1/2=1 3/max=2 -8=3<br>gen sev8v=rp30m10<br>recode sev8v 0=0 1/2=1 3/max=2 -8=3<br><br>gen SevPhysIPVvict=.<br>replace SevPhysIPVvict=1 if sev1v==1 | sev2v==1 | sev3v==1 | sev4v==1 | sev5v==1 | sev6v==1 | sev7v==1 | sev8v==1<br>replace SevPhysIPVvict=0 if sev1v==0 & sev2v==0 & sev3v==0 & sev4v==0 & sev5v==0 & sev6v==0 & sev7v==0 & sev8v==0<br>label variable SevPhysIPVvict "Severe physical IPV victimisation in the past year"<br>label define SevPhysIPVvict 0 "No" 1 "Yes"<br>label values SevPhysIPVvict SevPhysIPVvict	SevPhysIPVvict
**Christchurch Health and Development Study**	label z1496='IPV victimisation threw or tried to throw you bodily';<br>label z1498='IPV victimisation choked or strangled you';<br>label z1499='IPV victimisation kicked you';<br>label z1501='IPV victimisation punched or hit you with something';<br>label z1502='IPV victimisation slammed you into a wall';<br>label z1503='IPV victimisation burned or scaled you on purpose';<br>label z1504='IPV victimisation beat you up';<br>label z1505='IPV victimisation threatened you with knife or gun';<br>label z1506='IPV victimisation [illegible]	Frequencies for each behaviour	gen SevPhysIPVvict= .<br>replace SevPhysIPVvict = 1 if vict15==1 | vict17==1 | vict18==1 | vict20==1 | vict21==1 | vict22==1 | vict23==1 | vict24==1 | vict25==1<br>replace SevPhysIPVvict = 0 if vict15==0 & vict17==0 & vict18==0 & vict20==0 & vict21==0 & vict22==0 & vict23==0 & vict24==0 & vict25==0<br>label variable SevPhysIPVvict "Any severe IPV victimisation in past year"<br>label define SevPhysIPVvict 0 "No" 1 "Yes"<br>label values SevPhysIPVvict SevPhysIPVvict<br>tab SevPhysIPVvict	SevPhysIPVvict

**National Longitudinal Study of Adolescent to Adult Health**	H4RD19 - How often (has/did) {initials} (slapped/slap), hit or (kicked/kick) you?<br>H4RD20 - How often (have/did) you (had/have) an injury, such as a sprain, bruise, or cut because of a fight with {initials}?	Frequencies for each behaviour	gen sevIPVvict1 = H4RD19<br>recode sevIPVvict1 0/1=0 2/7=1 96=. 98=.<br>label variable sevIPVvict1 "IPV victimisation (slapping, kicking, hitting)"<br>label define sevIPVvict1 0 "No" 1 "Yes" 2 "Dont know, refused"<br>label values sevIPVvict1 sevIPVvict1<br>tab sevIPVvict1 H4RD19<br><br>gen sevIPVvict2= H4RD20<br>recode sevIPVvict2 0/1=0 2/7=1 97=0 98=. 96=.<br>label variable sevIPVvict2 "IPv victimisation - had an injury e.g. a cut, sprain or bruise, in the past year"<br>label define sevIPVvict2 0 "No" 1 "Yes" 2 "Dont know, refused"<br>label values sevIPVvict2 sevIPVvict2<br>tab sevIPVvict2 H4RD20<br><br>gen SevPhysIPVvict = .<br>replace SevPhysIPVvict= 0 if sevIPVvict1 == 0 & sevIPVvict2 == 0<br>replace SevPhysIPVvict= 1 if sevIPVvict1 == 1 | sevIPVvict2 == 1<br>label variable SevPhysIPVvict "Severe IPV victimisation in the past year"<br>label define SevPhysIPVvict 0 "No" 1 "Yes"<br>label values SevPhysIPVvict SevPhysIPVvict<br>tab SevPhysIPVvict<br>tab SevPhysIPVvict H4RD19	SevPhysIPVvict
**APMS**	N/A	N/A	N/A	N/A
**National survey of families and households**	Have YOU been cut, bruised, or seriously injured in a fight with your partner?<br>Have YOU been cut, bruised, or seriously injured in a fight with your husband/wife?	Binary	gen SevPhysIPVvict=IPVvictPY<br>replace SevPhysIPVvict=3 if physIPVvict2==1 | physIPVvict4==1<br>recode SevPhysIPVvict 0/1=0 3=1<br>label variable SevPhysIPVvict"Any severe IPV victimisation in the past year"<br>label define SevPhysIPVvict 0 "No" 1 "Yes"<br>label values SevPhysIPVvict SevPhysIPVvict<br>tab SevPhysIPVvict	SevPhysIPVvict
