# Supplementary material for: Past-year intimate partner violence perpetration among people with and without depression: an individual participant data (IPD) meta-mediation analysis
Source: Soc Psychiatry Psychiatr Epidemiol. 2021 Nov 29;58(12):1735–47. doi: 10.1007/s00127-021-02183-w (PMC10627935; doi:10.1007/s00127-021-02183-w)
Supplement: Supplementary file 2 — Supplementary file2 (PDF 4237 KB) [file 127_2021_2183_MOESM2_ESM.pdf]

Supplementary material

Random vs. fixed effects meta-analysis results

Article title:
Past year intimate partner violence perpetration among people with depression: an individual participant data (IPD) meta-mediation analysis.
Journal name:
Social Psychiatry and Psychiatric Epidemiology.

Author names:
Katherine Saunders[1], Sabine Landau[1,2], Louise M Howard[1], Helen L. Fisher[3,4], Louise Arseneault[3,4], Geraldine F H McLeod[5], Sian Oram[1]
Affiliation:
[1] King's College London, London, Health Service and Population Research Department, Institute of Psychiatry, Psychology & Neuroscience, London, United Kingdom
[2] King's College London, London, Biostatistics and Health Informatics Department, Institute of Psychiatry, Psychology & Neuroscience, London, United Kingdom
[3] King's College London, London, Social, Genetic & Developmental Psychiatry Centre, Institute of Psychiatry, Psychology & Neuroscience, London, United Kingdom
[4] ESRC Centre for Society and Mental Health, King's College London, London, United Kingdom
[5] Department of Psychological Medicine, University of Otago Christchurch, New Zealand
E-mail address of the corresponding author:
sian.oram@kcl.ac.uk

Alcohol: men

Total effect: In fixed effects meta-analysis, the proportion of difference in physical IPV perpetration between men with and without depression was 4.8% (95% CI 2.6-6.9). In random effects meta-analysis, the proportion of difference was 5.1% (95% CI 1.3-8.9).

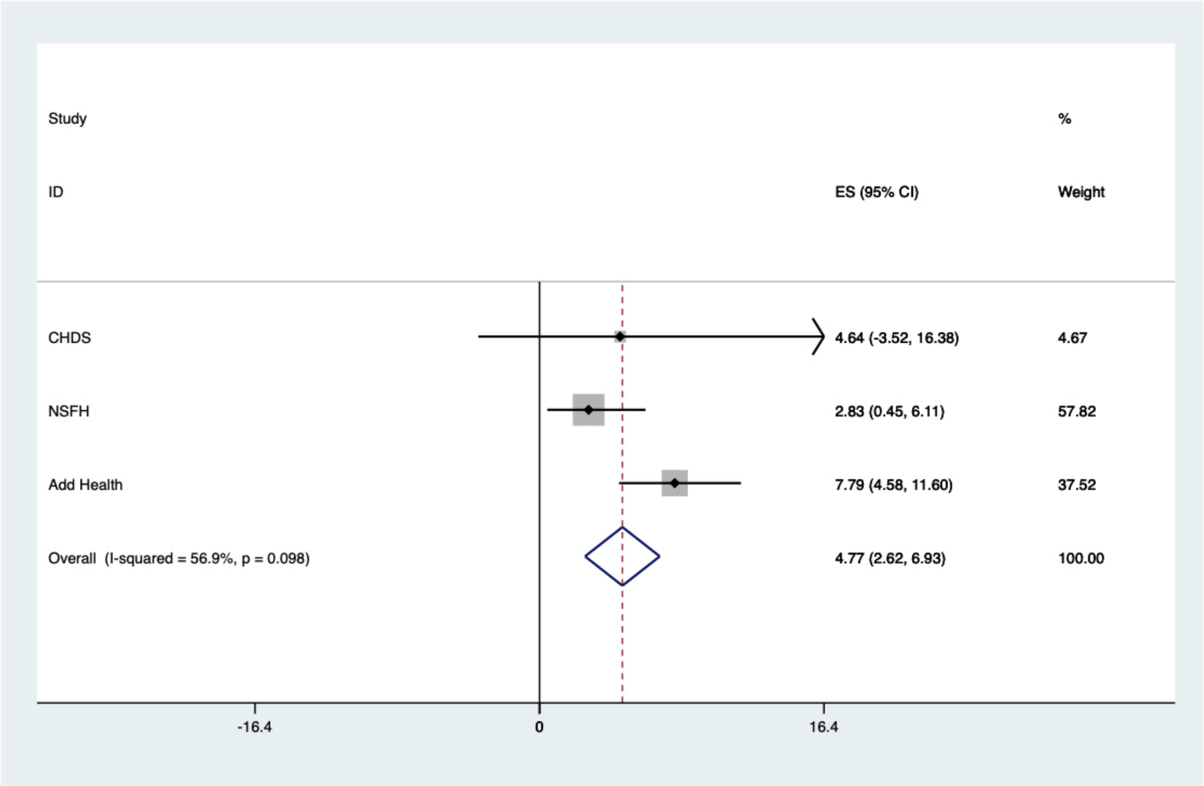

Figure 1 Results of fixed effects meta-analysis of the percentage differences between those with and without depression in past year physical IPV perpetration.

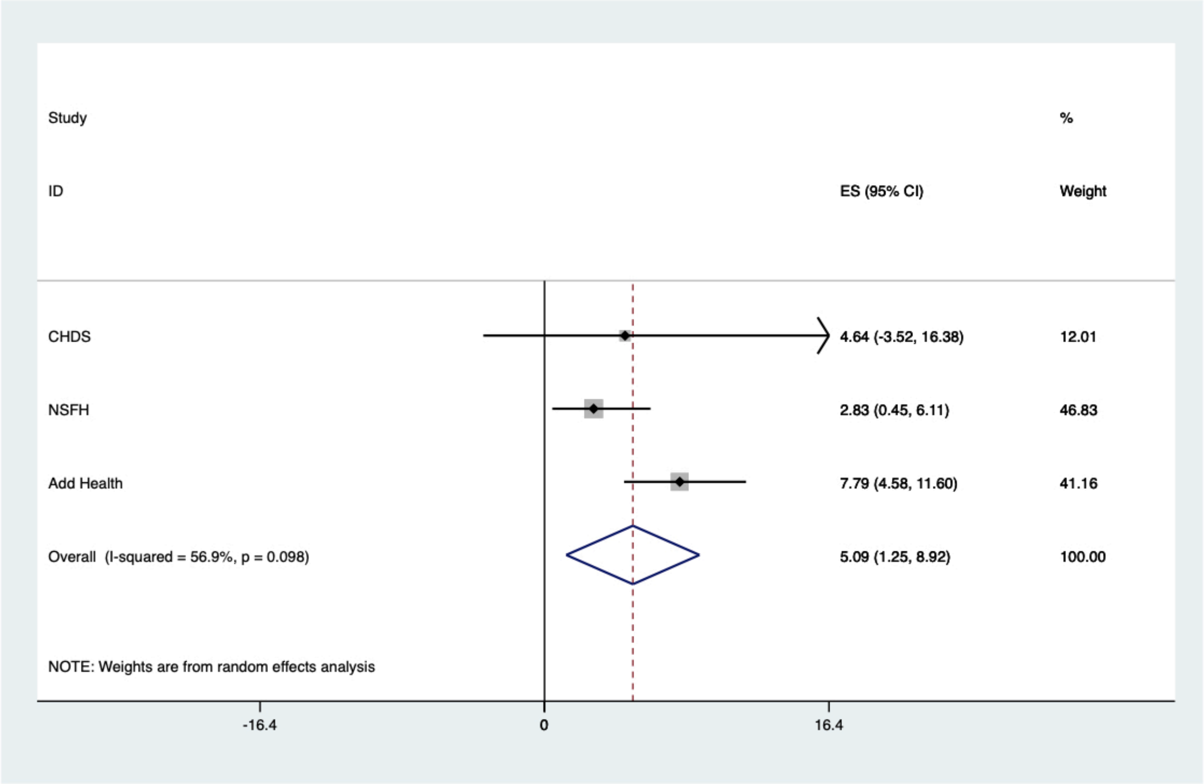

Figure 2 Results of random effects meta-analysis of the percentage differences between those with and without depression in past year physical IPV perpetration.

Indirect effect: In fixed effects meta-analyses, no part of the total effect was mediated by alcohol (0.02%, 95% CI -0.1-0.2). In random effects meta-analyses, no part of the total effect was mediated by alcohol (0.1%, 95% CI -0.2%-0.4%).

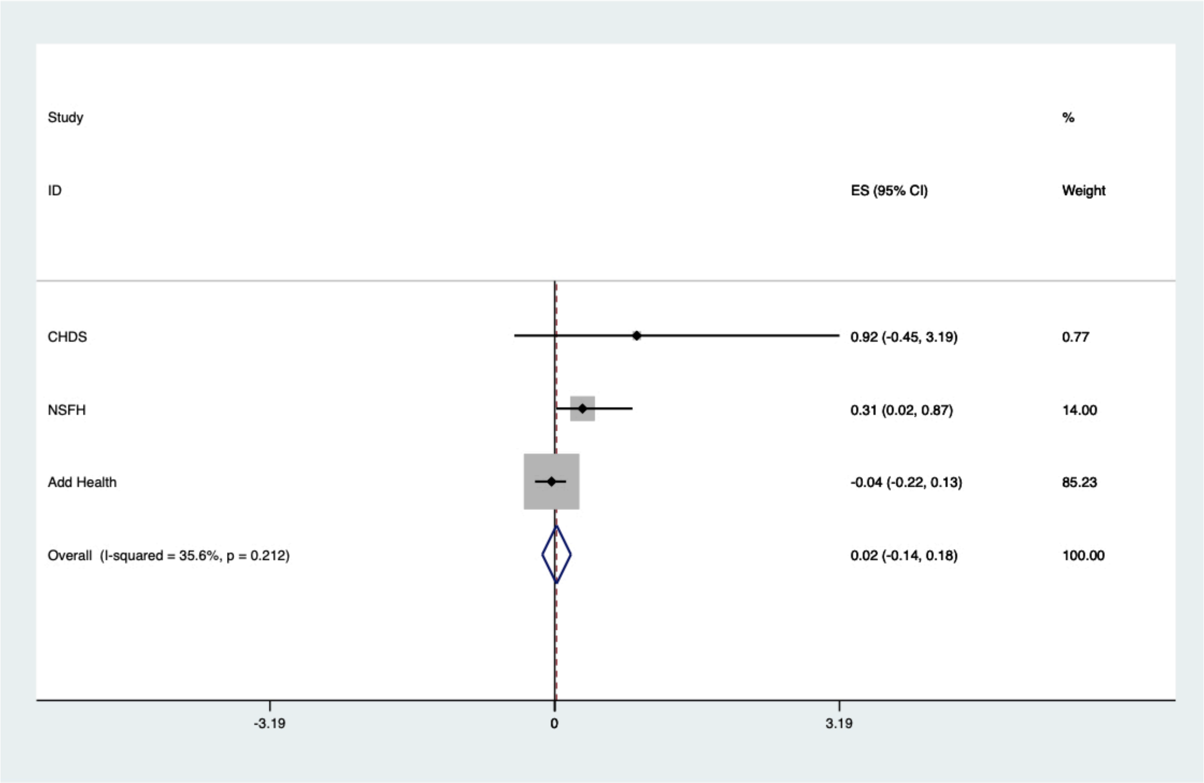

Figure 3 Results of fixed effects meta-analysis of the mediated percentage differences between men with and without depression in past year physical IPV perpetration.

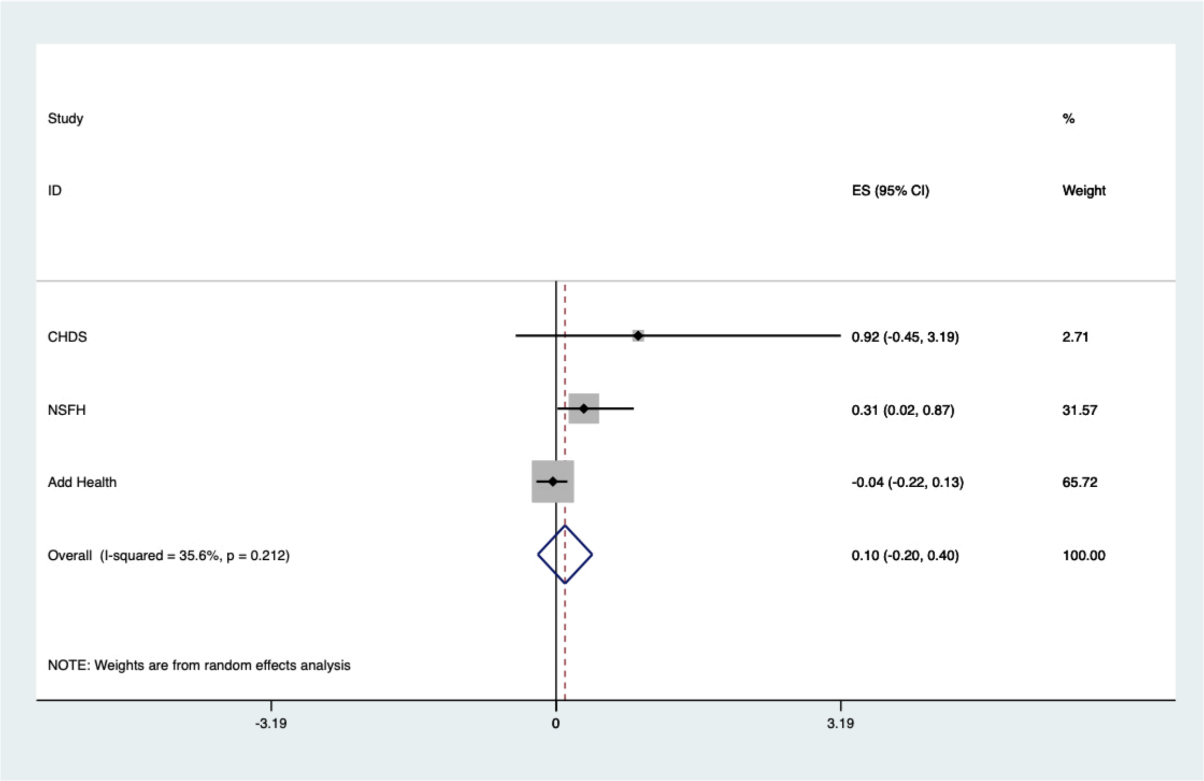

Figure 4 Results of random effects meta-analysis of the mediated percentage differences between men with and without depression in past year physical IPV perpetration.

Direct effect: In fixed effects meta-analysis, 4.6% (95% CI 2.4-6.7) of the total effect of depression on past year physical IPV perpetration among men was unmediated by alcohol. In random effects meta-analysis, 4.9% (95% CI 0.7%-9.0%) of the total effect of depression on past year physical IPV perpetration among men was unmediated by alcohol.

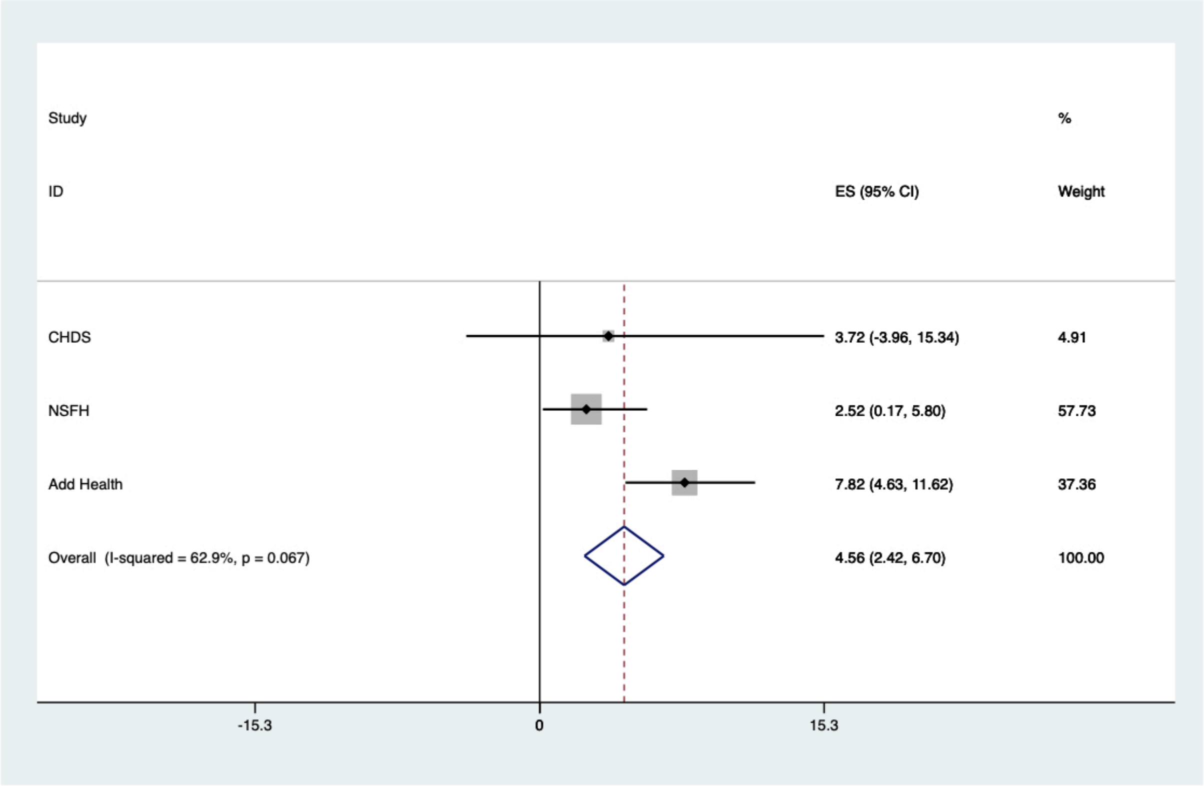

Figure 5 Results of fixed effects meta-analysis of the unmediated percentage differences between men with and without depression in past year physical IPV perpetration.

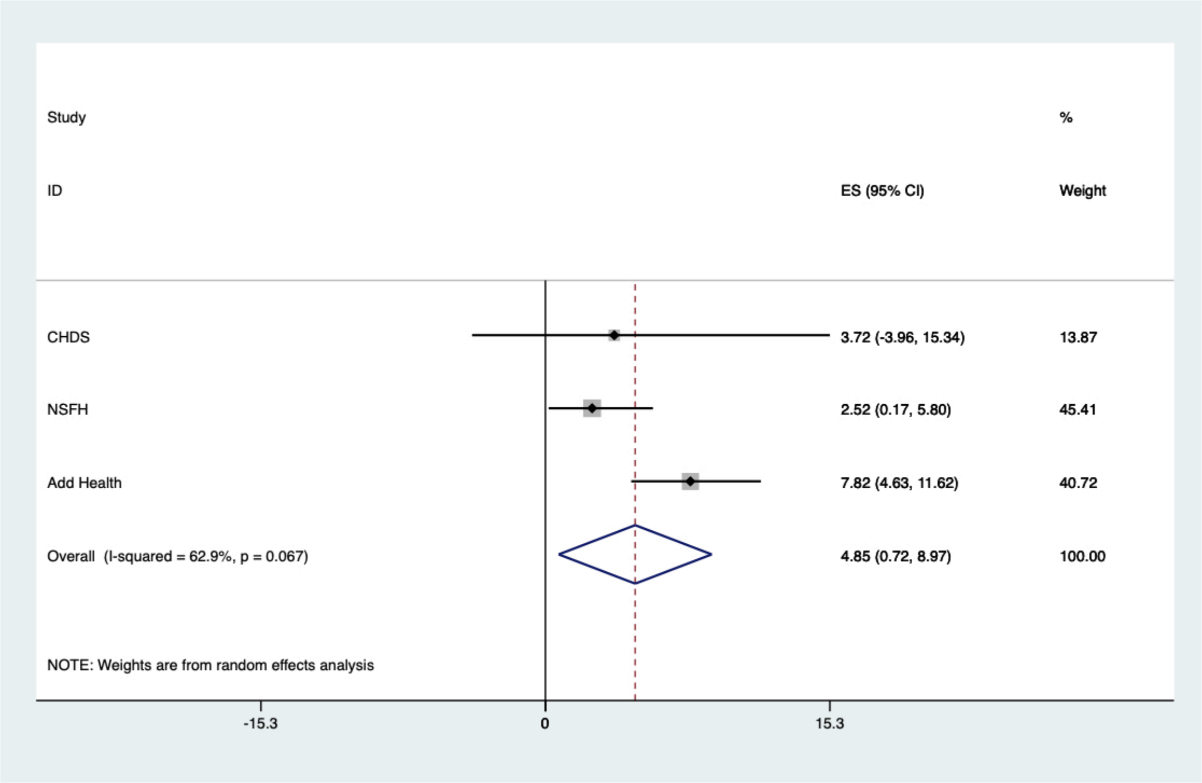

Figure 6 Results of random effects meta-analysis of the unmediated percentage differences between men with and without depression in past year physical IPV perpetration.

Alcohol: Women

Total effect: In fixed effects meta-analyses, the proportion of difference in physical IPV perpetration between women with and without depression was 7.2% (95% CI 4.1-10.2). In random effects meta-analyses, the proportion of difference in physical IPV perpetration between women with and without depression was 7.7% (95% CI 3.7%-11.7%).

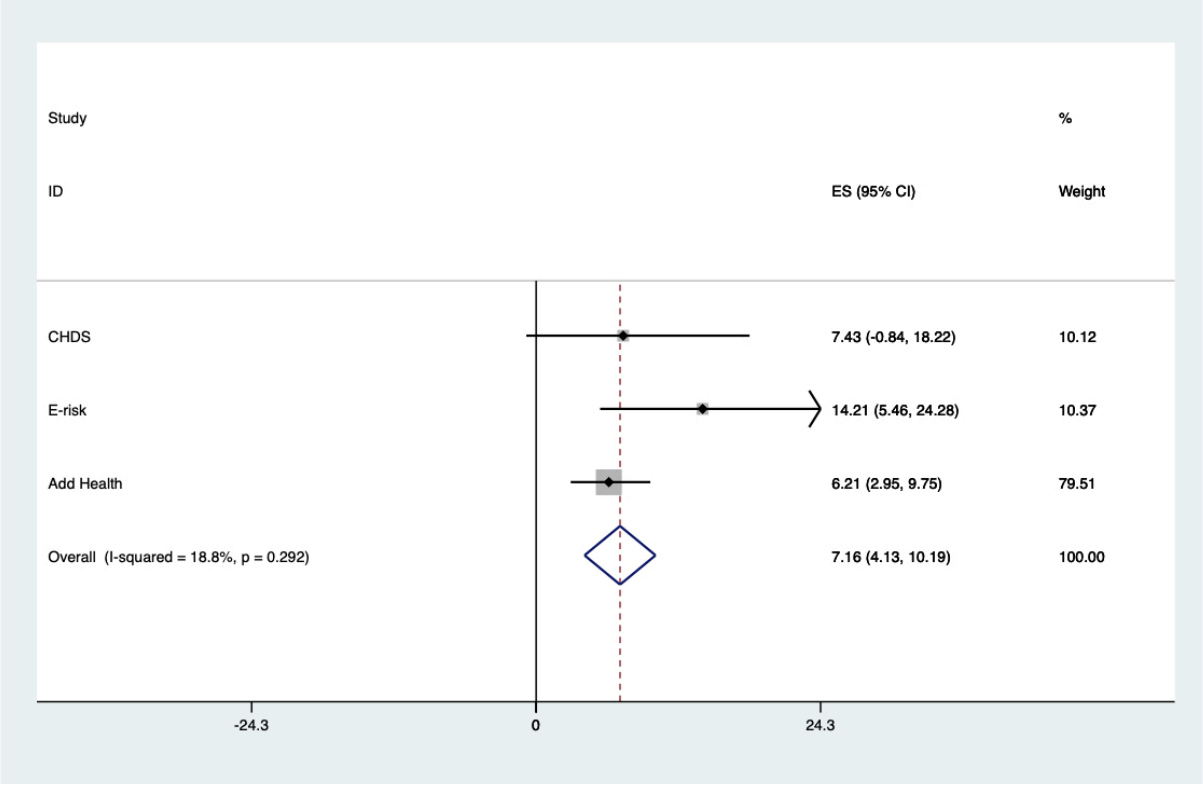

Figure 7 Results of fixed effects meta-analysis of the percentage differences between women with and without depression in past year physical IPV perpetration.

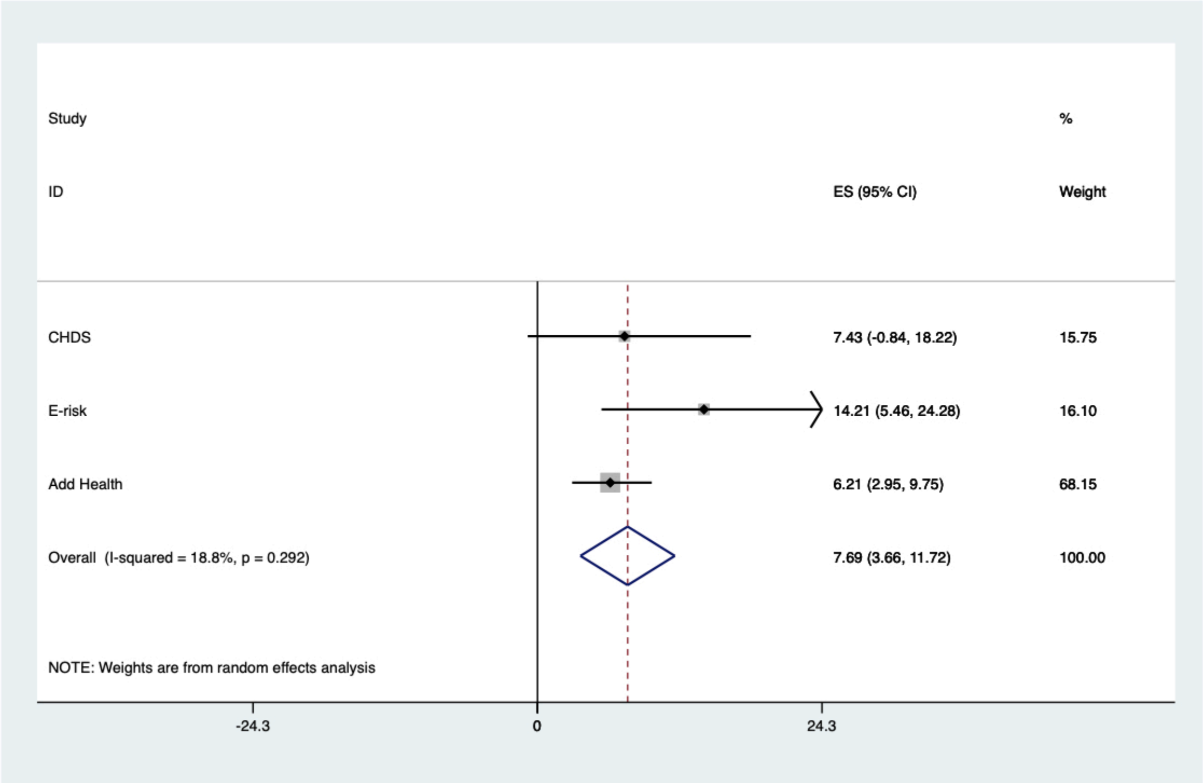

Figure 8 Results of random effects meta-analysis of the percentage differences between women with and without depression in past year physical IPV perpetration.

Indirect effect: In fixed effects meta-analysis, no part of the total effect was mediated by alcohol (0.04%, 95% CI -0.1-0.2). In random effects meta-analysis, no part of the total effect was mediated by alcohol (0.8%, 95% CI -0.5-2.1).

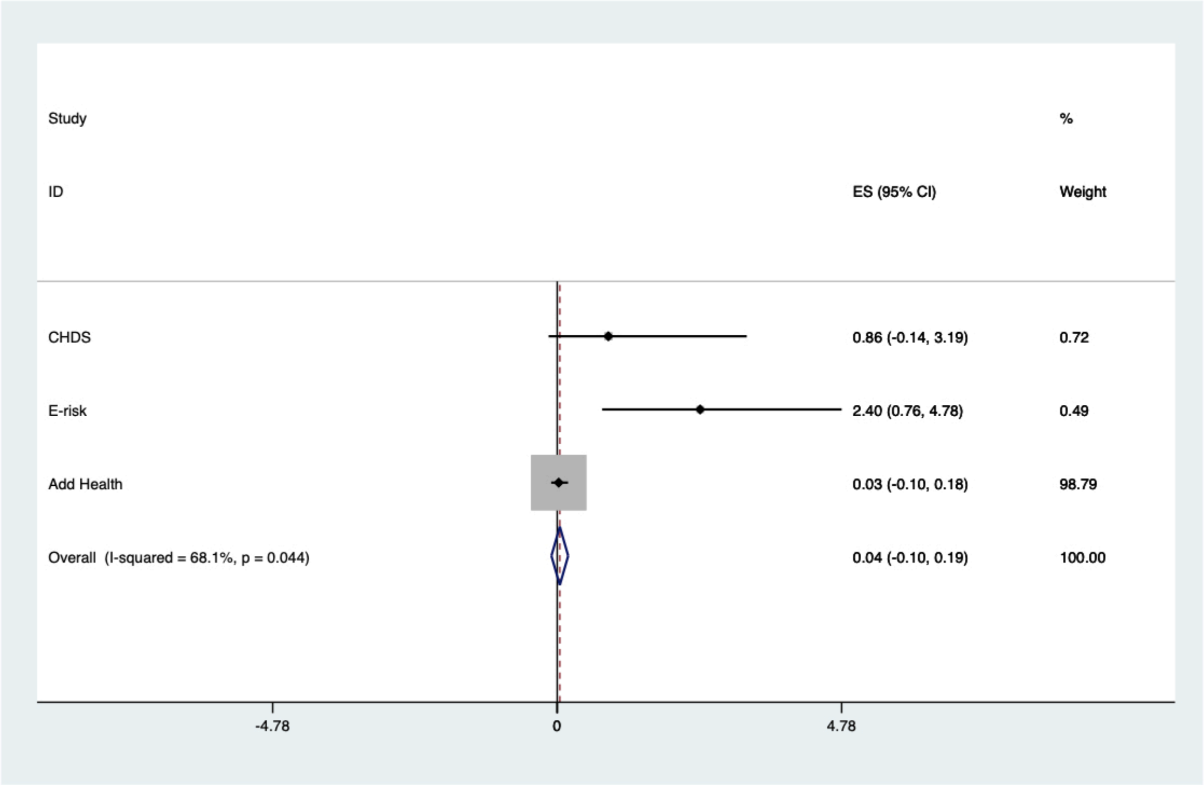

Figure 9 Results of fixed effects meta-analysis of the mediated percentage differences between women with and without depression in past year physical IPV perpetration.

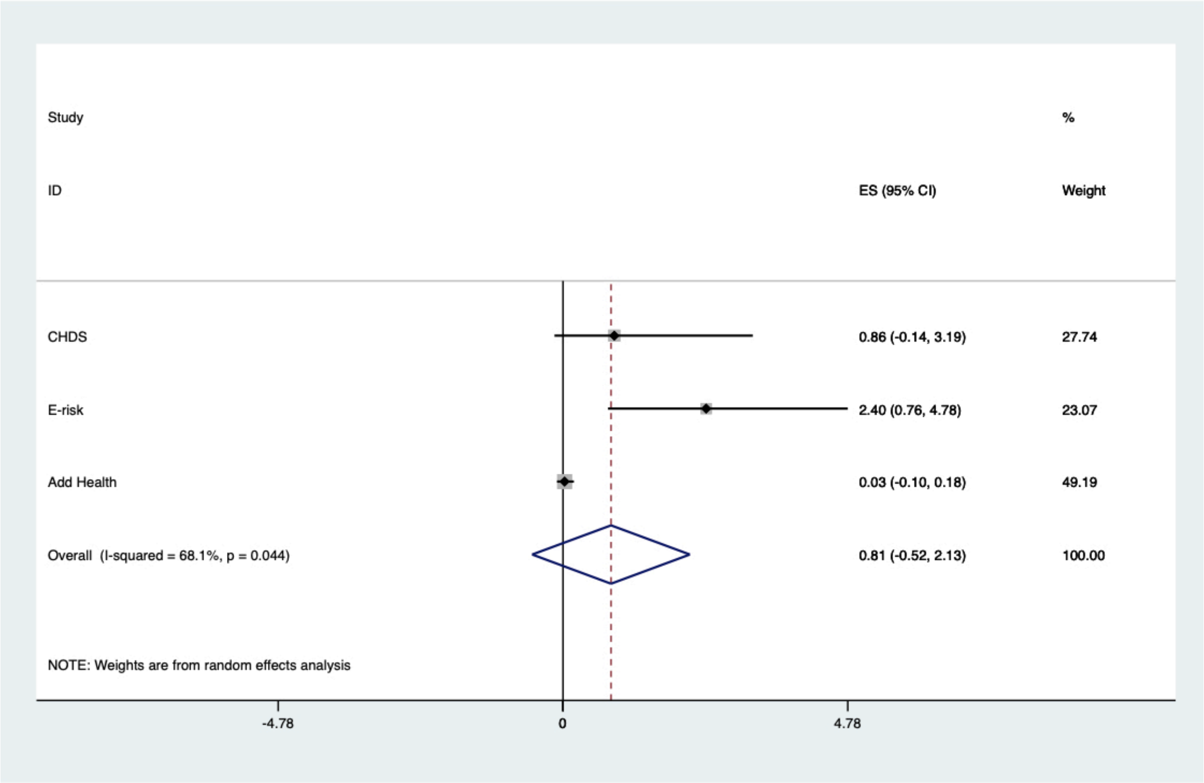

Figure 10 Results of random effects meta-analysis of the mediated percentage differences between women with and without depression in past year physical IPV perpetration.

Direct effect: In fixed effects meta-analysis, 6.6% (95% CI 3.5-9.7) of the total effect of depression on past year physical IPV perpetration among women was unmediated by alcohol. In random effects meta-analysis, results were identical.

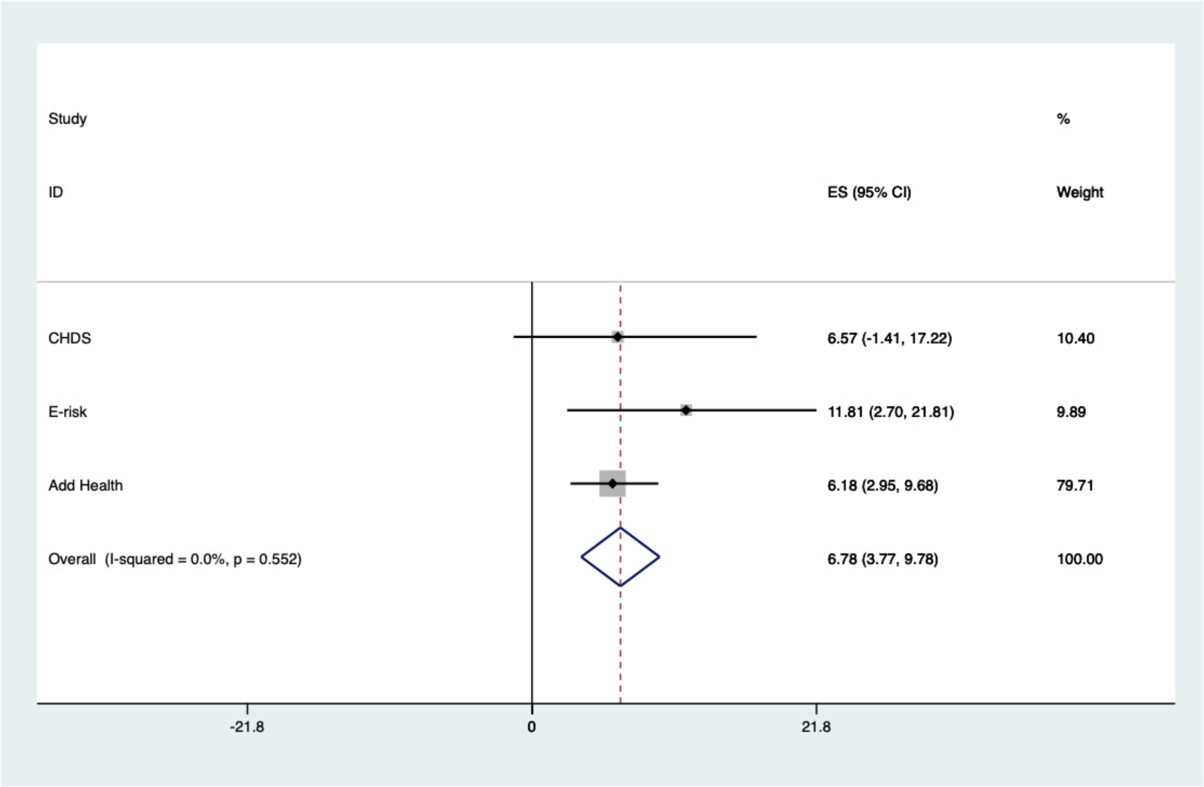

Figure 11 Results of fixed effects meta-analysis of the unmediated percentage differences between women with and without depression in past year physical IPV perpetration.

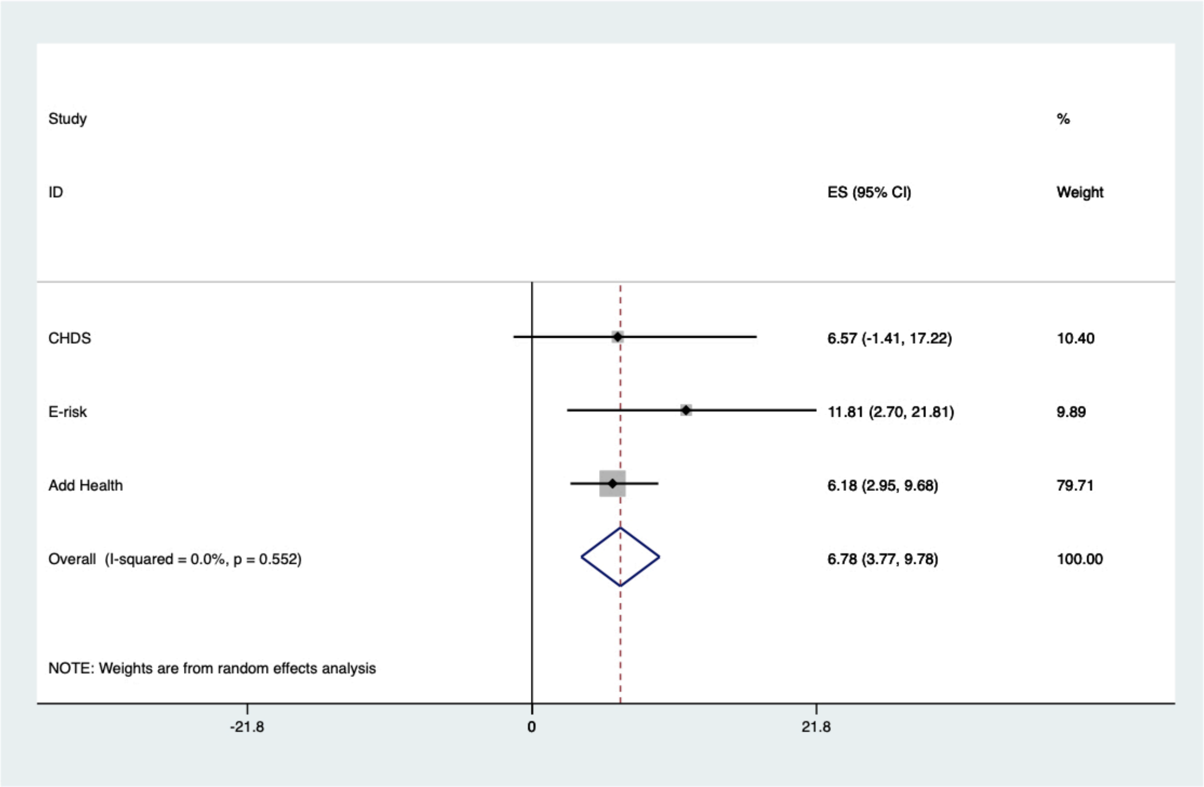

Figure 12 Results of random effects meta-analysis of the unmediated percentage differences between women with and without depression in past year physical IPV perpetration.

IPV victimisation: women

Total effect: In fixed effects meta-analysis, the proportion of difference in physical IPV perpetration between women with and without depression was 7.4% (95% CI 4.3-10.6). In random effects meta-analysis, the proportion of difference in physical IPV perpetration between women with and without depression was 8.4% (95% CI 3.6-13.1)

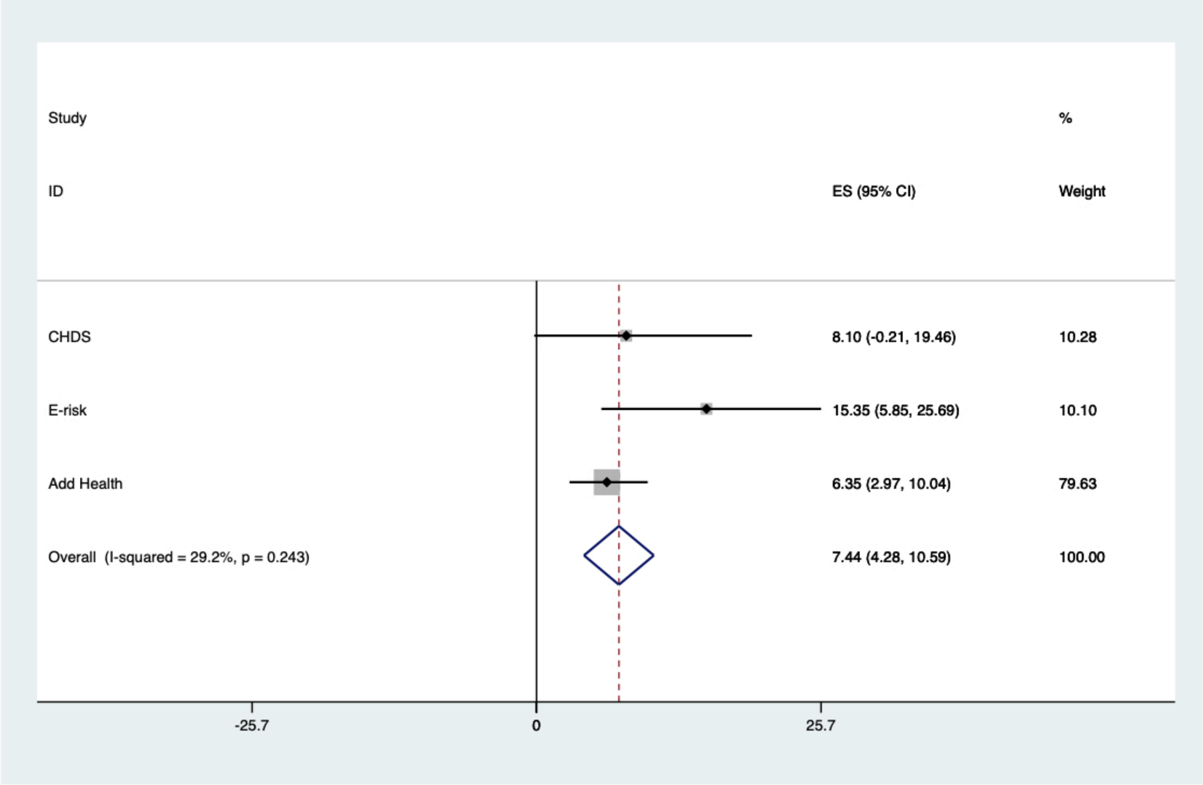

Figure 13 Results of fixed effects meta-analysis of the percentage differences between women with and without depression in past year physical IPV perpetration.

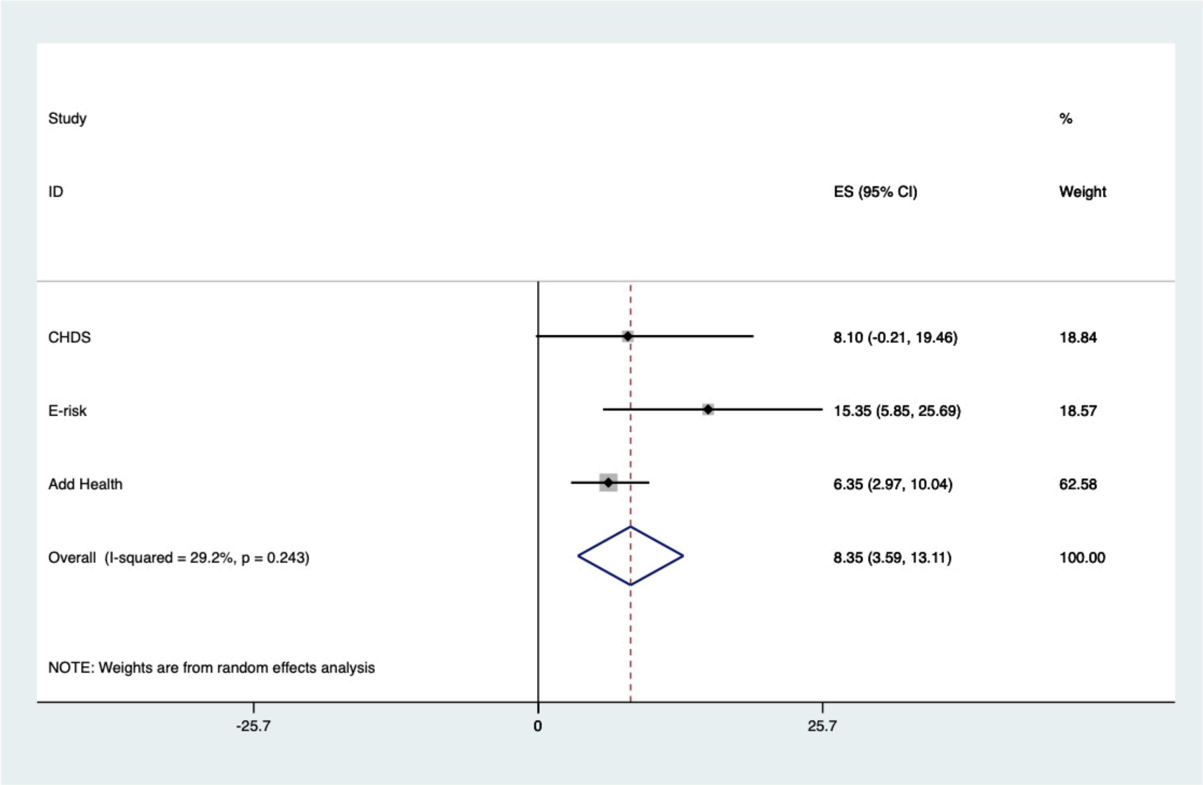

Figure 14 Results of random effects meta-analysis of the percentage differences between women with and without depression in past year physical IPV perpetration.

Indirect effect: In fixed effects meta-analysis, 3.3% (95% CI 2.0-4.5%) of the total effect of depression on past year physical IPV perpetration among women was mediated by IPV victimisation. In random effects meta-analysis, 4.7% (95% CI 0.9-8.5) of the total effect of depression on past year physical IPV perpetration among women was mediated by IPV victimisation.

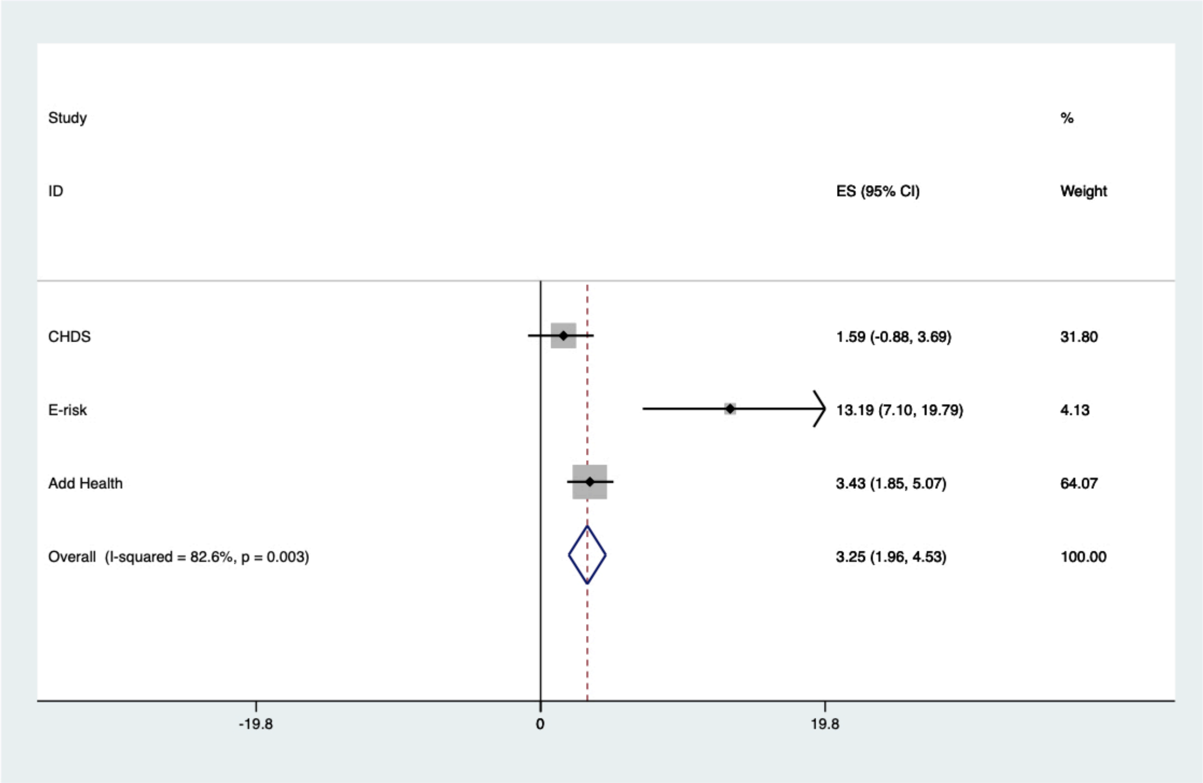

Figure 15 Results of fixed effects meta-analysis of the mediated percentage differences between women with and without depression in past year physical IPV perpetration.

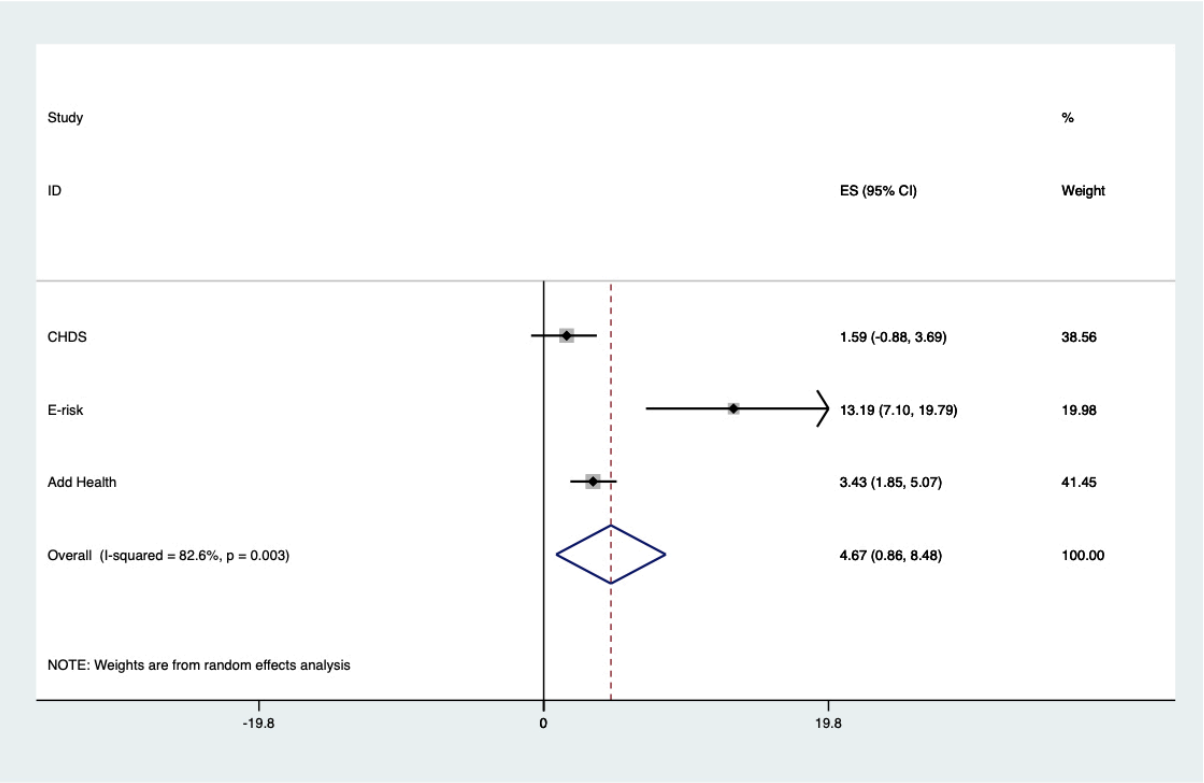

Figure 16 Results of random effects meta-analysis of the mediated percentage differences between women with and without depression in past year physical IPV perpetration.

Direct effect: In fixed effects meta-analysis, 3.1% (95% CI 0.3-6.0) of the total effect of depression on past year physical IPV perpetration among women was unmediated by IPV victimisation. In random effects meta-analysis, results were identical.

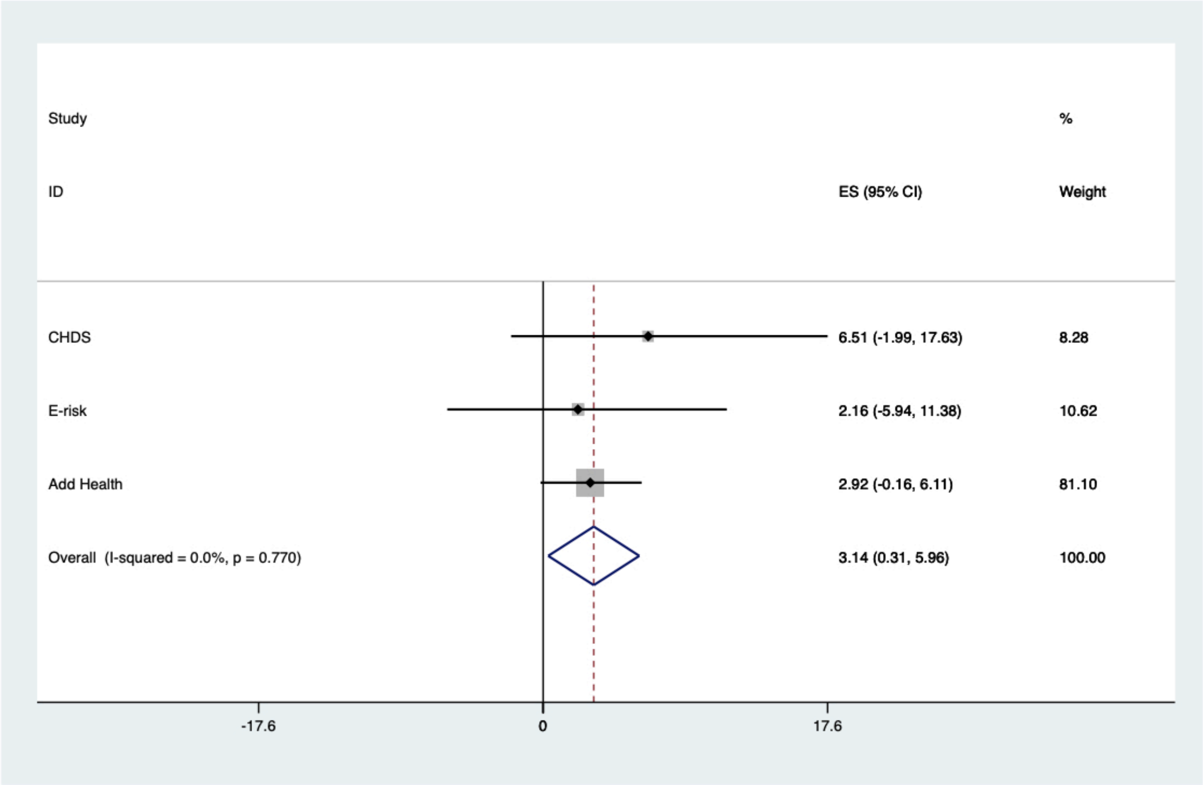

Figure 17 Results of fixed effects meta-analysis of the unmediated percentage differences between women with and without depression in past year physical IPV perpetration.

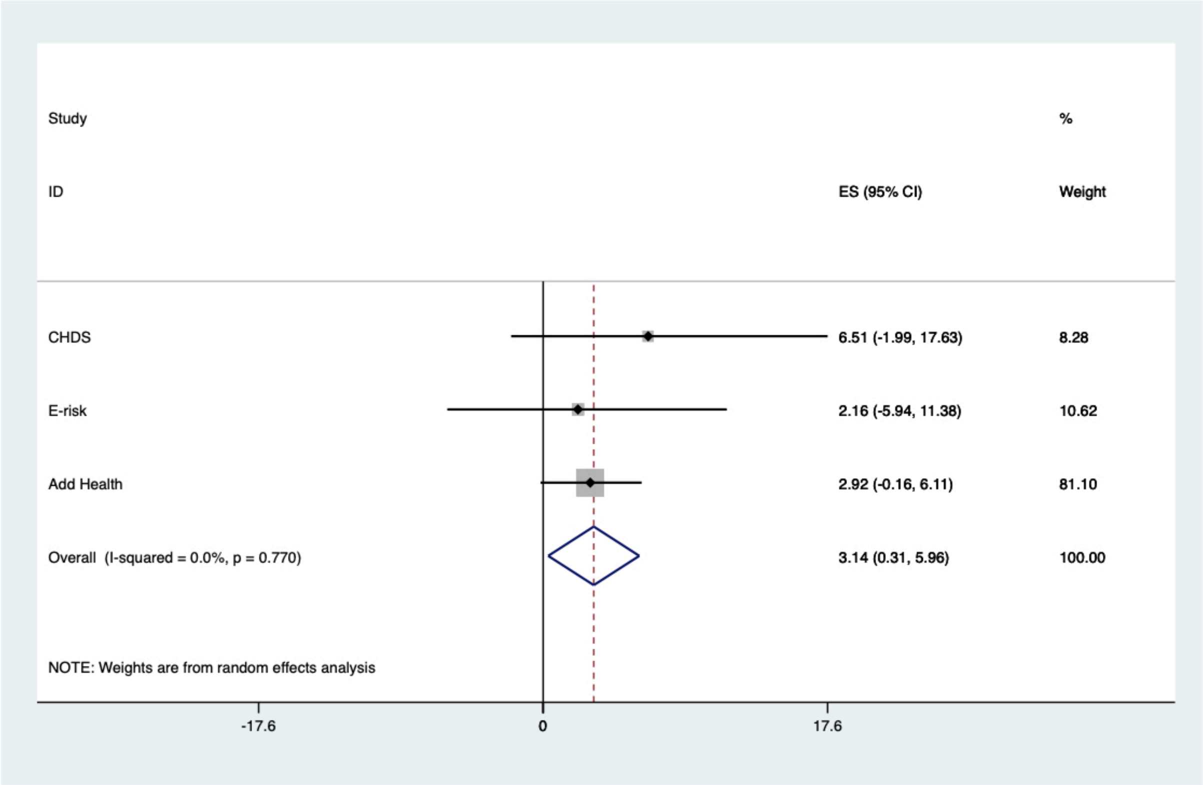

Figure 18 Results of random effects meta-analysis of the unmediated percentage differences between women with and without depression in past year physical IPV perpetration.

Severe IPV victimisation: women

Total effect: In fixed effects meta-analysis, the proportion of difference in physical IPV perpetration between women with and without depression was 7.4% (95% CI 4.3%-10.4%). In random effects meta-analysis, the proportion of difference in physical IPV perpetration between women with and without depression was 7.8% (95% CI 3.8-11.8).

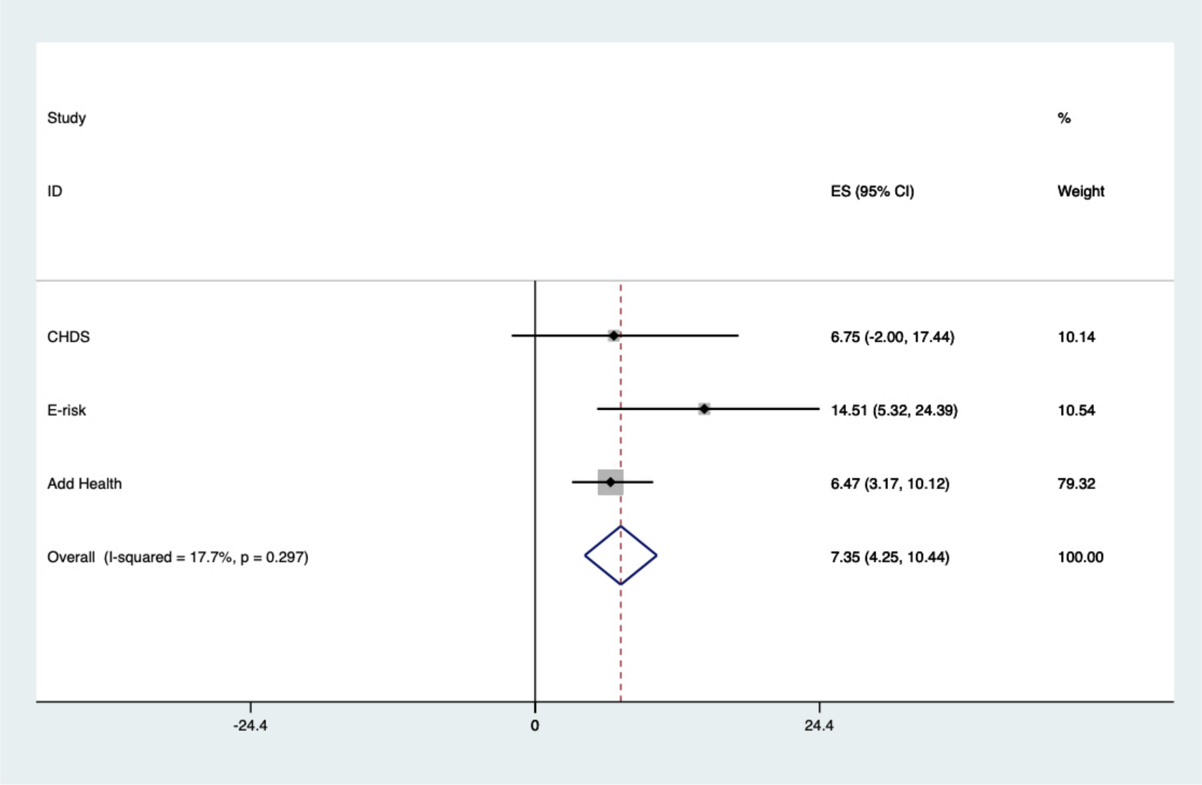

Figure 19 Results of fixed effects meta-analysis of the percentage differences between women with and without depression in past year physical IPV perpetration.

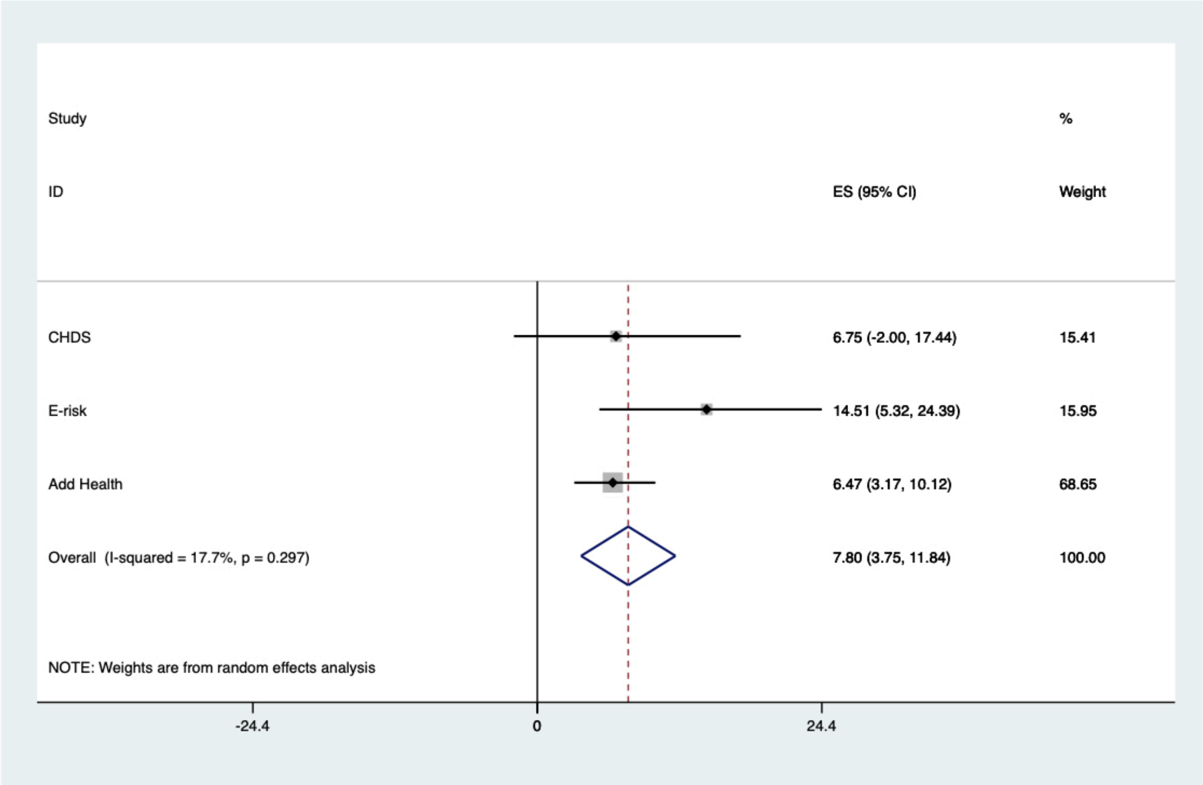

Figure 20 Results of random effects meta-analysis of the percentage differences between women with and without depression in past year physical IPV perpetration

Indirect effect: In fixed effects meta-analysis, 1.9% (95% CI 0.7-3.2) of the total effect was mediated by severe IPV victimisation. In random effects meta-analysis, the results were identical.

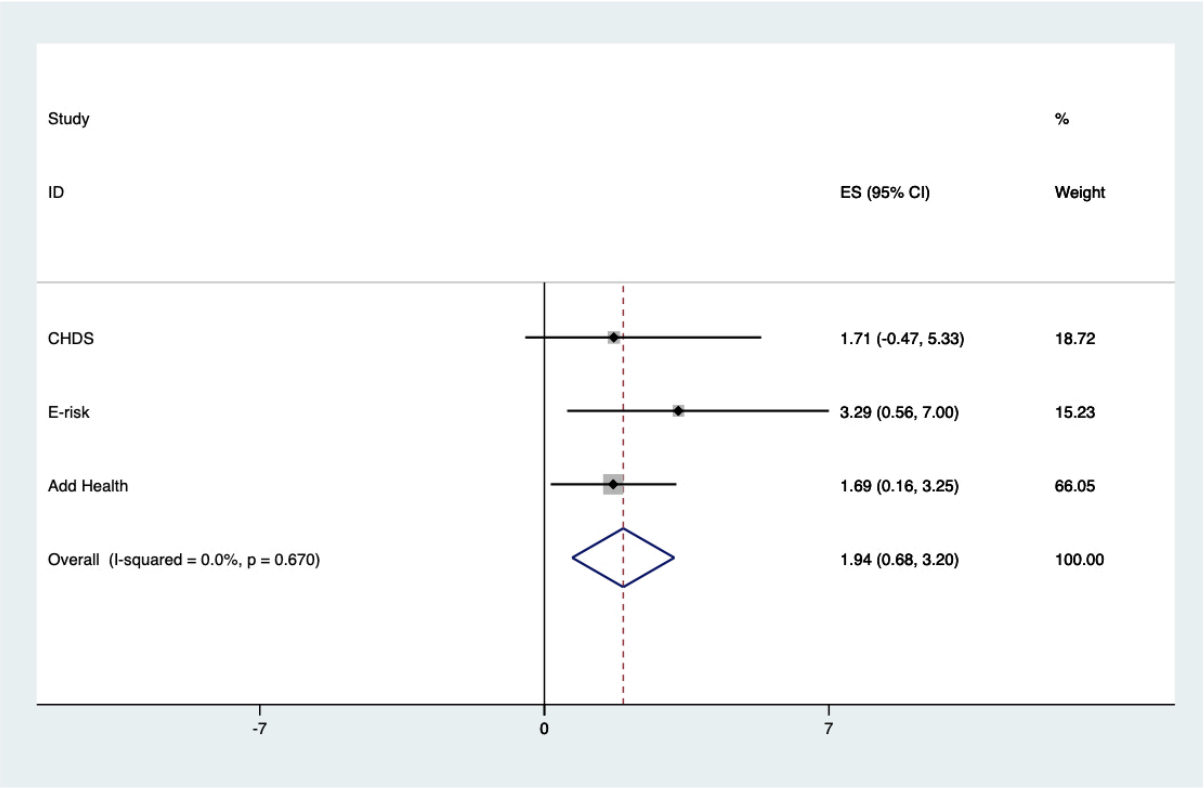

Figure 21 Results of fixed effects meta-analysis of the mediated percentage differences between women with and without depression in past year physical IPV perpetration.

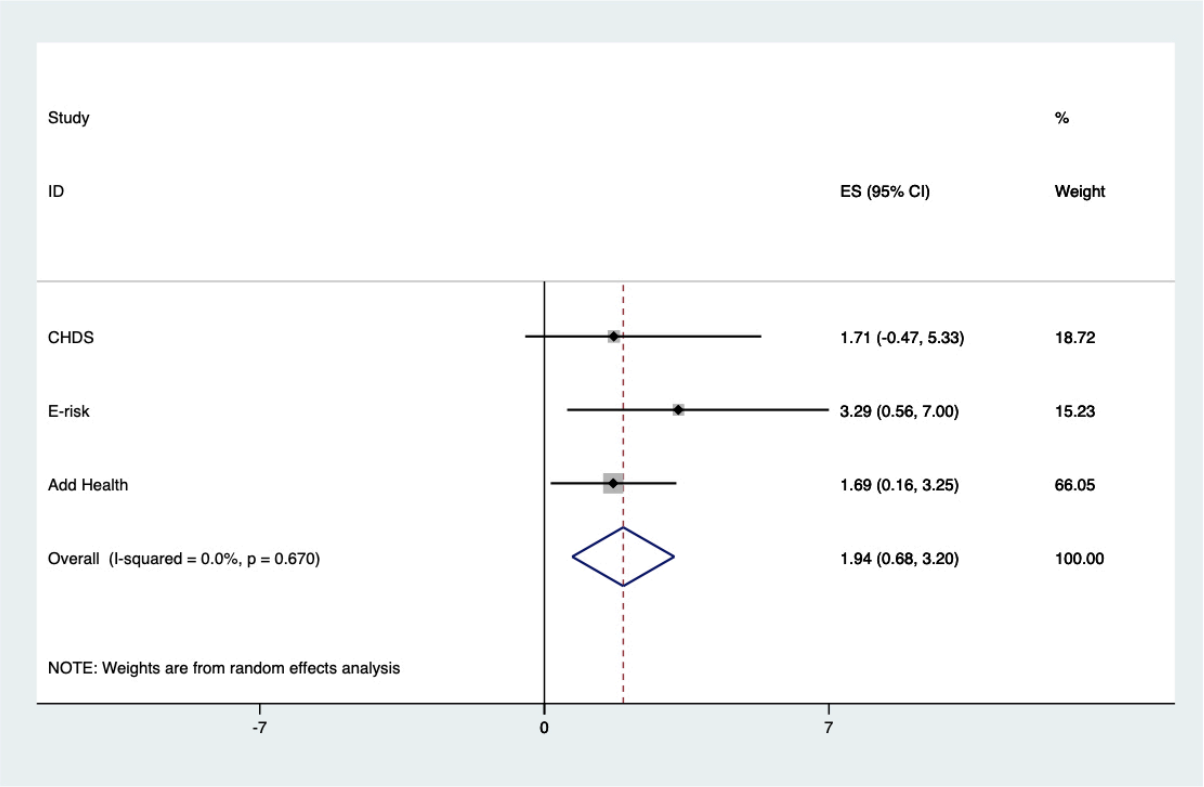

Figure 22 Results of random effects meta-analysis of the mediated percentage differences between women with and without depression in past year physical IPV perpetration.

Direct effect: In fixed effects meta-analysis, 5.4% (95% CI 2.5-8.2) of the total effect of depression on past year physical IPV perpetration among women was unmediated by severe IPV victimisation. In random effects meta-analysis, the results were identical.

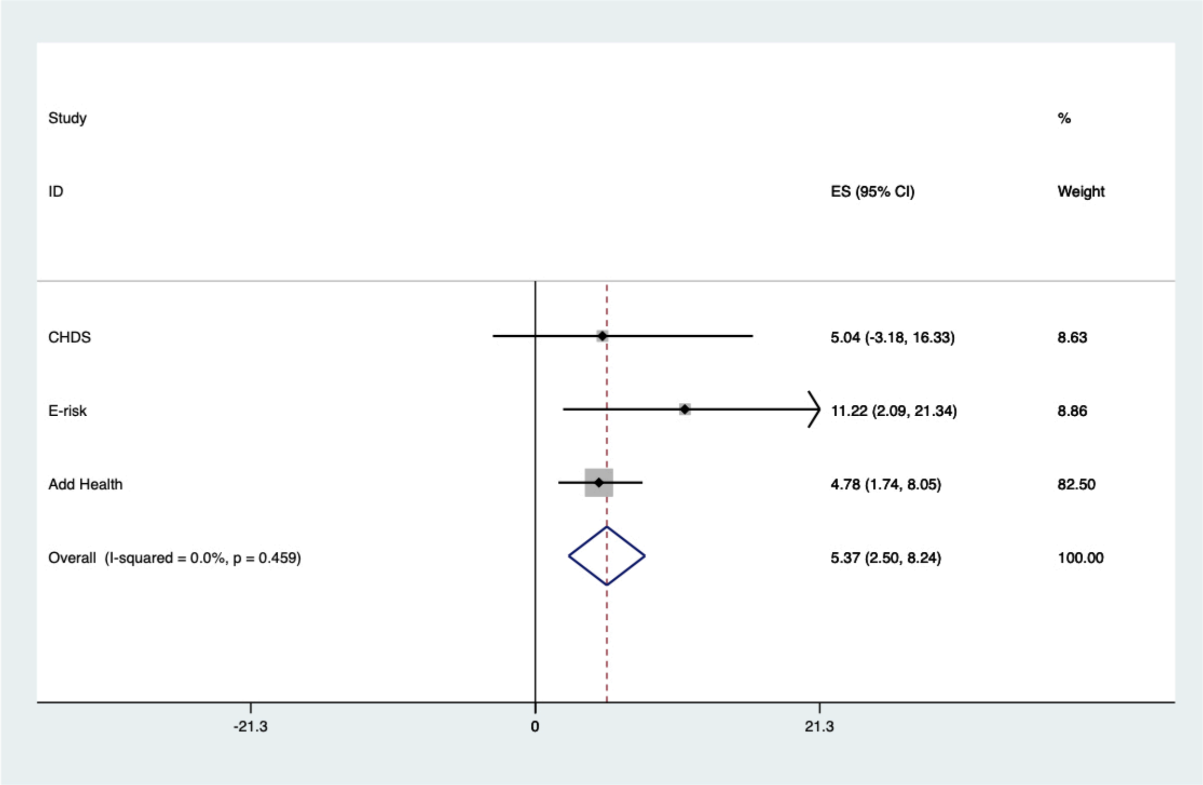

Figure 23 Results of fixed effects meta-analysis of the unmediated percentage differences between women with and without depression in past year physical IPV perpetration.

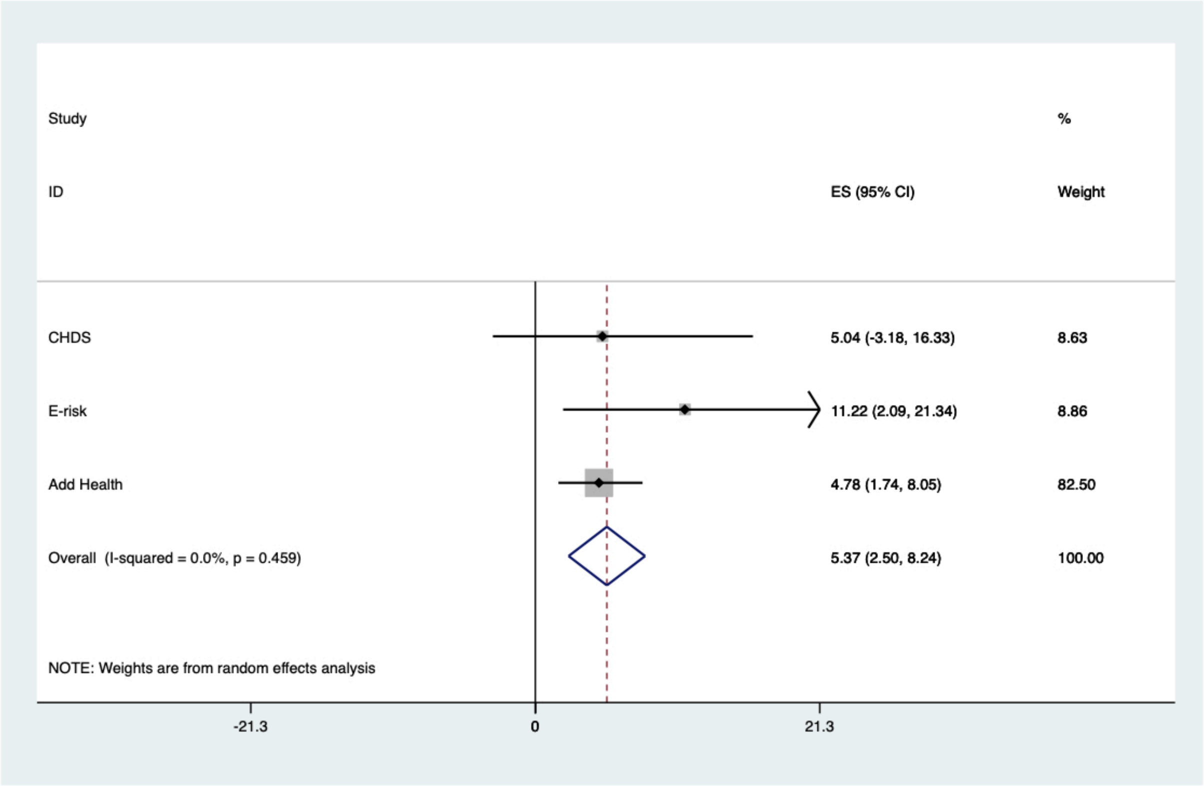

Figure 24 Results of random effects meta-analysis of the unmediated percentage differences between women with and without depression in past year physical IPV perpetration.

Mediation meta-analysis results for past year severe physical IPV perpetration:

IPV victimisation: Women

Total effect: In fixed effects meta-analysis, the proportion of difference in severe physical IPV perpetration between women with and without depression was 4.0% (95% CI 1.5-6.6). In random effects meta-analysis, the proportion of difference in severe physical IPV perpetration between women with and without depression was 5.4% (95% CI 0.1%-10.6%).

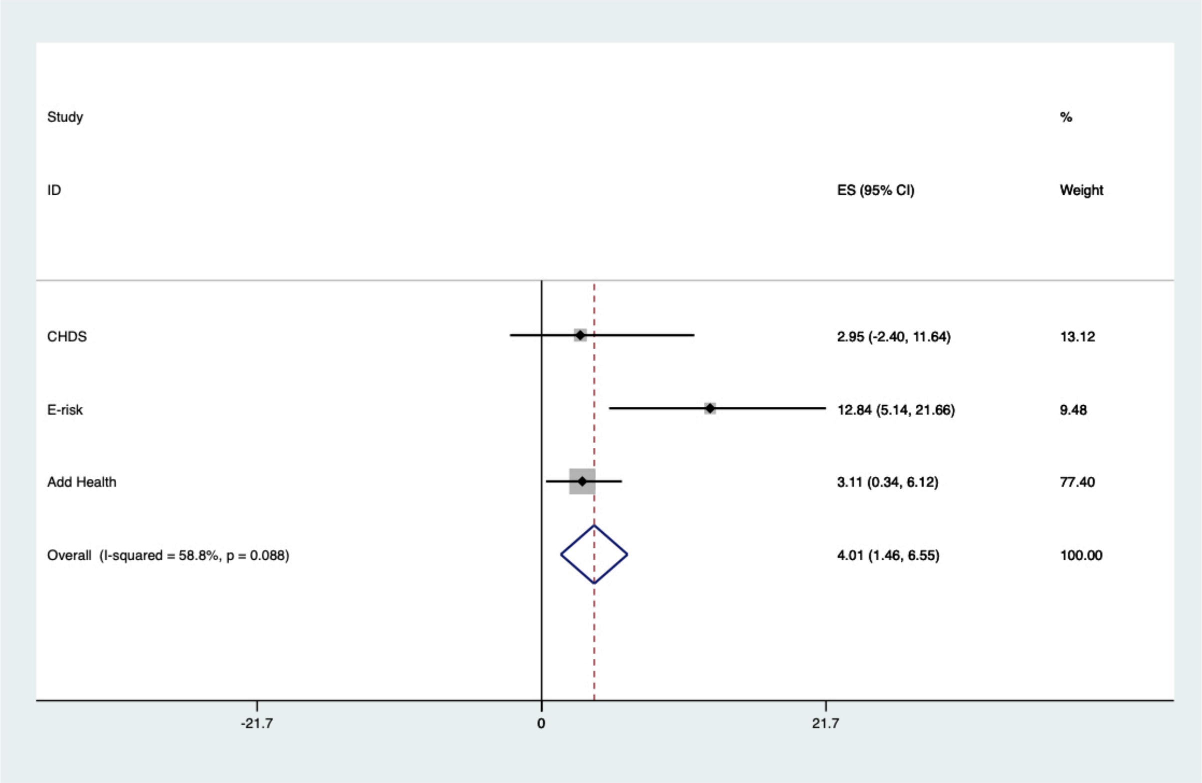

Figure 25 Results of fixed effects meta-analysis of the percentage differences between women with and without depression in past year severe physical IPV perpetration.

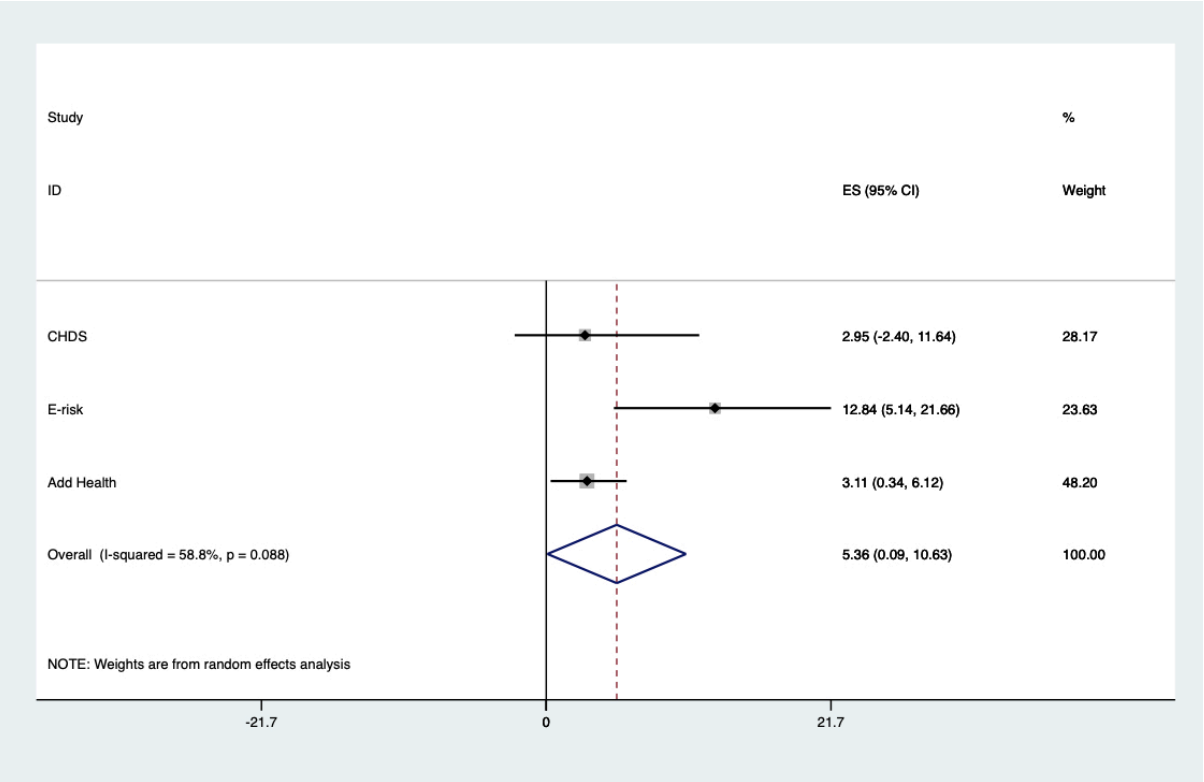

Figure 26 Results of random effects meta-analysis of the percentage differences between women with and without depression in past year severe physical IPV perpetration.

Indirect effect: In fixed effects meta-analysis, 2.1% (95% CI 1.1-3.1) of the total effect of depression on past year severe physical IPV perpetration among women was mediated by IPV victimisation. In random effects meta-analysis, the total effect of depression on past year severe physical IPV perpetration among women was not mediated by IPV victimisation (2.9%, 95% CI -0.3%-6.1%).

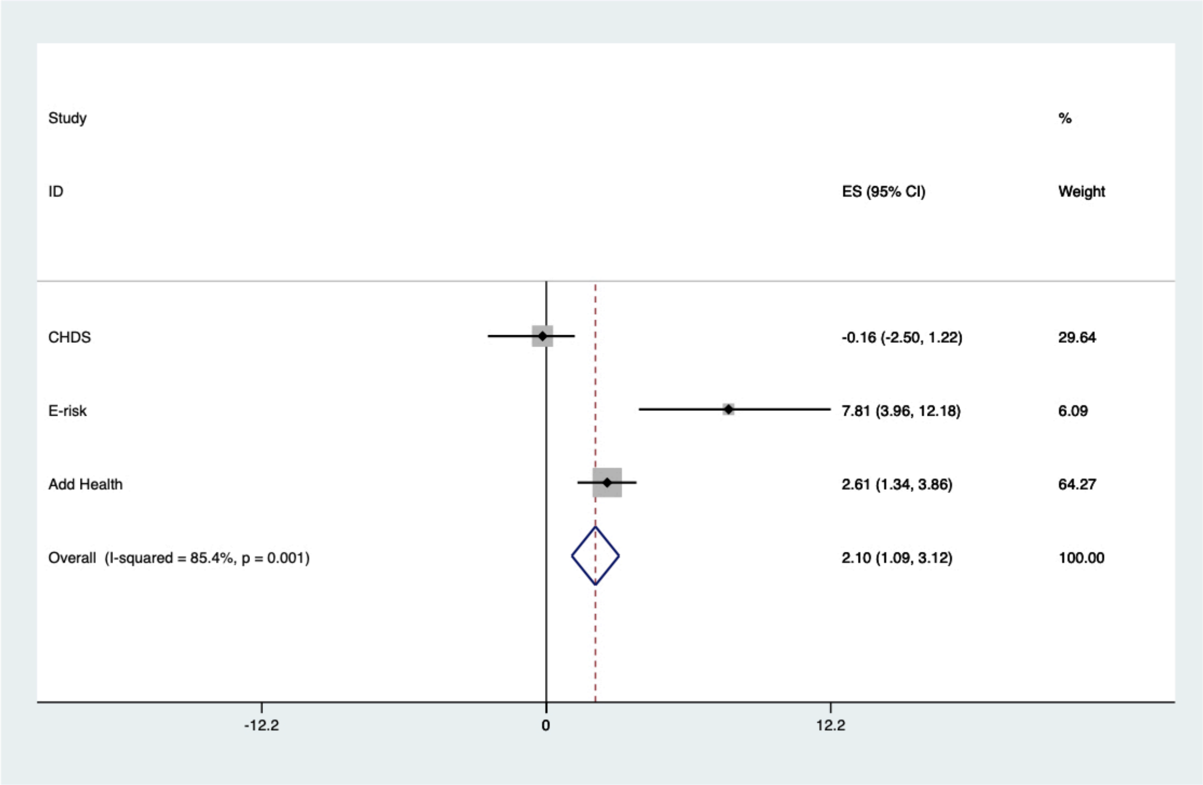

Figure 27 Results of fixed effects meta-analysis of the mediated percentage differences between women with and without depression in past year physical IPV perpetration.

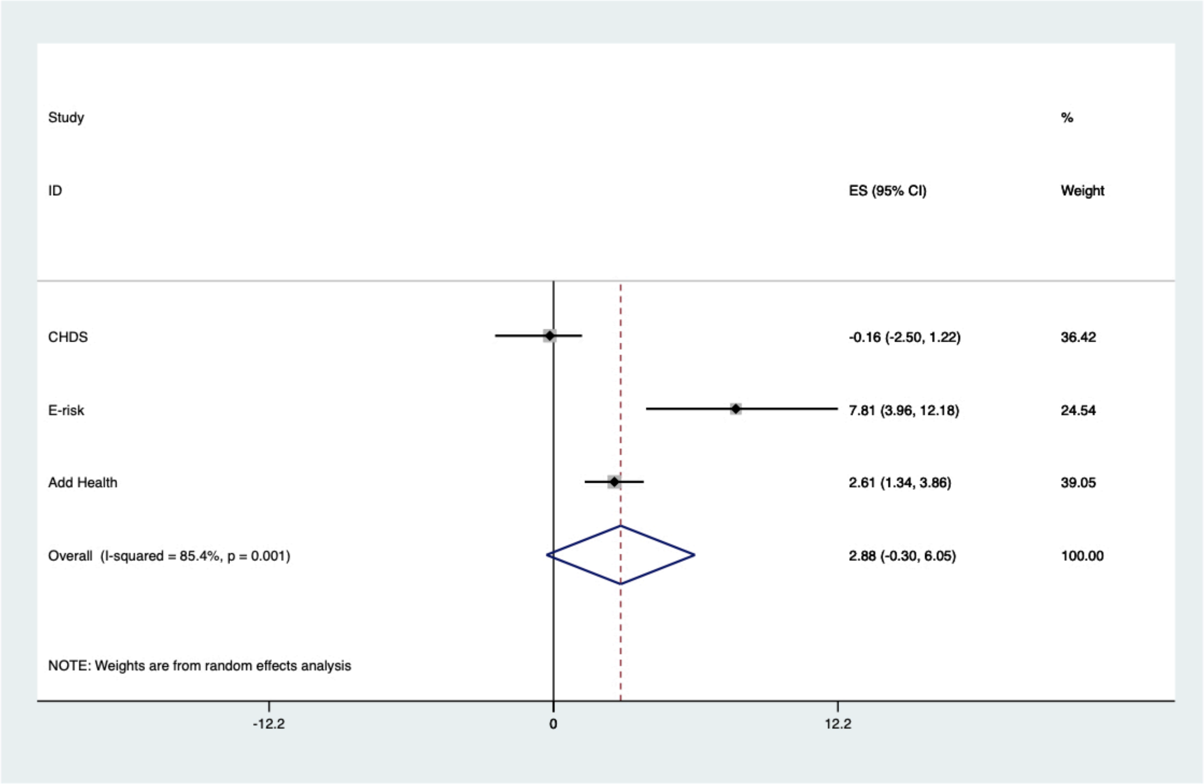

Figure 28 Results of random effects meta-analysis of the mediated percentage differences between women with and without depression in past year physical IPV perpetration.

Direct effect: In fixed effects meta-analysis, there was no direct effect of depression on severe IPV perpetration (1.3%, 95% CI -1.03-3.5). In random effects meta-analysis, the results were identical.

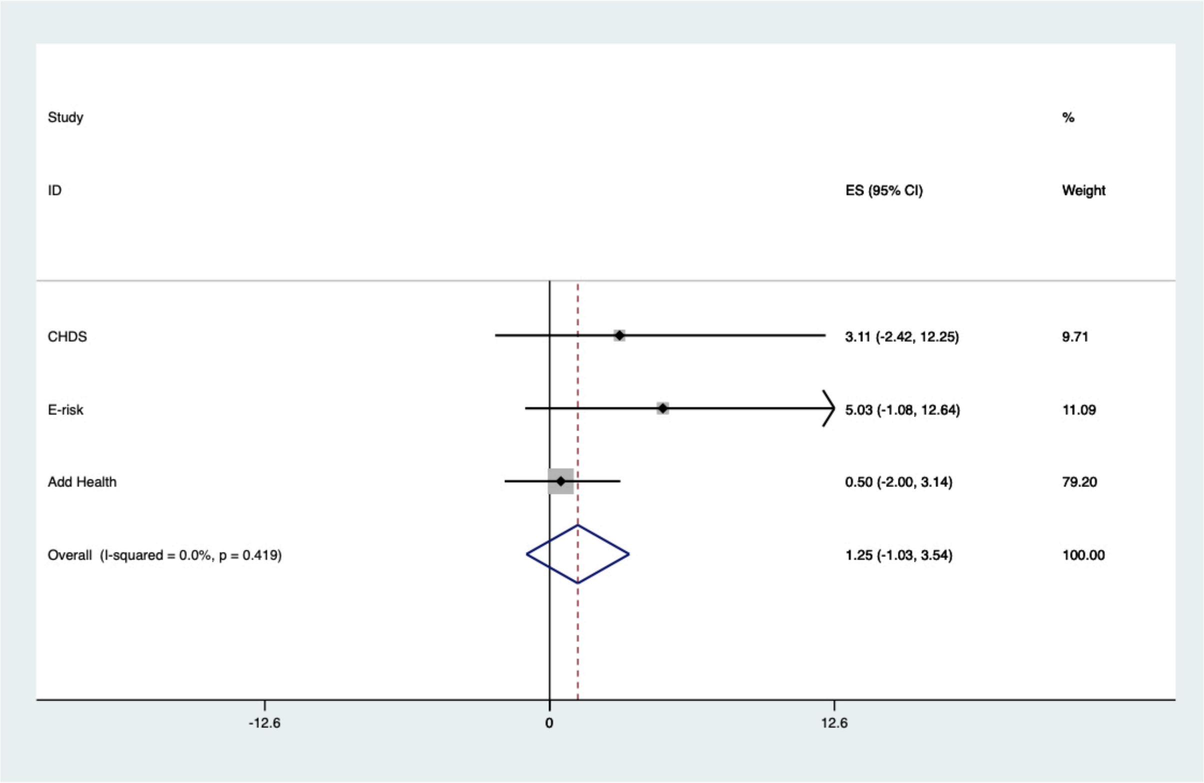

Figure 29 Results of fixed effects meta-analysis of the non-mediated percentage differences between women with and without depression in past year severe physical IPV perpetration.

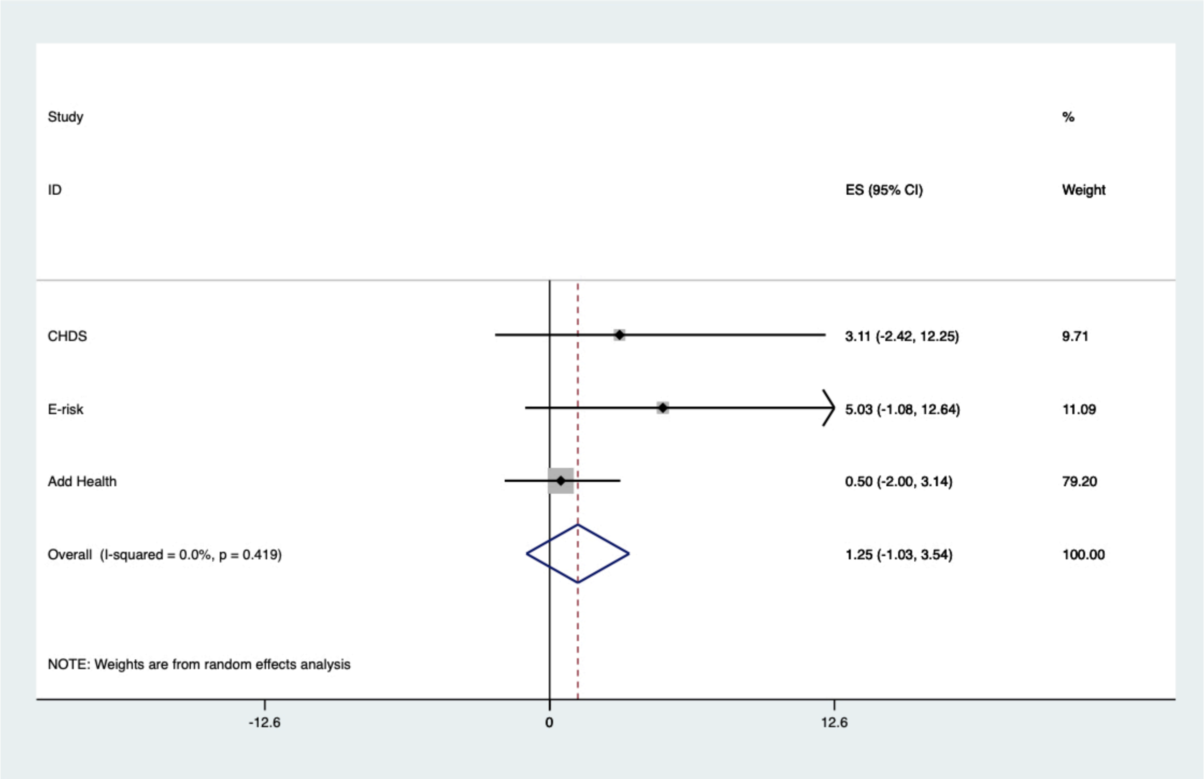

Figure 30 Results of random effects meta-analysis of the non-mediated percentage differences between women with and without depression in past year severe physical IPV perpetration.

Severe IPV victimisation: women

Total effect: In fixed-effects meta-analysis, the proportion of difference in severe physical IPV perpetration between women with and without depression was 2.0% (95% CI 0.6%-3.3%). In random-effects meta-analysis, the proportion of difference in severe physical IPV perpetration between women with and without depression was 3.3% (95% CI 0.2%-6.3%).

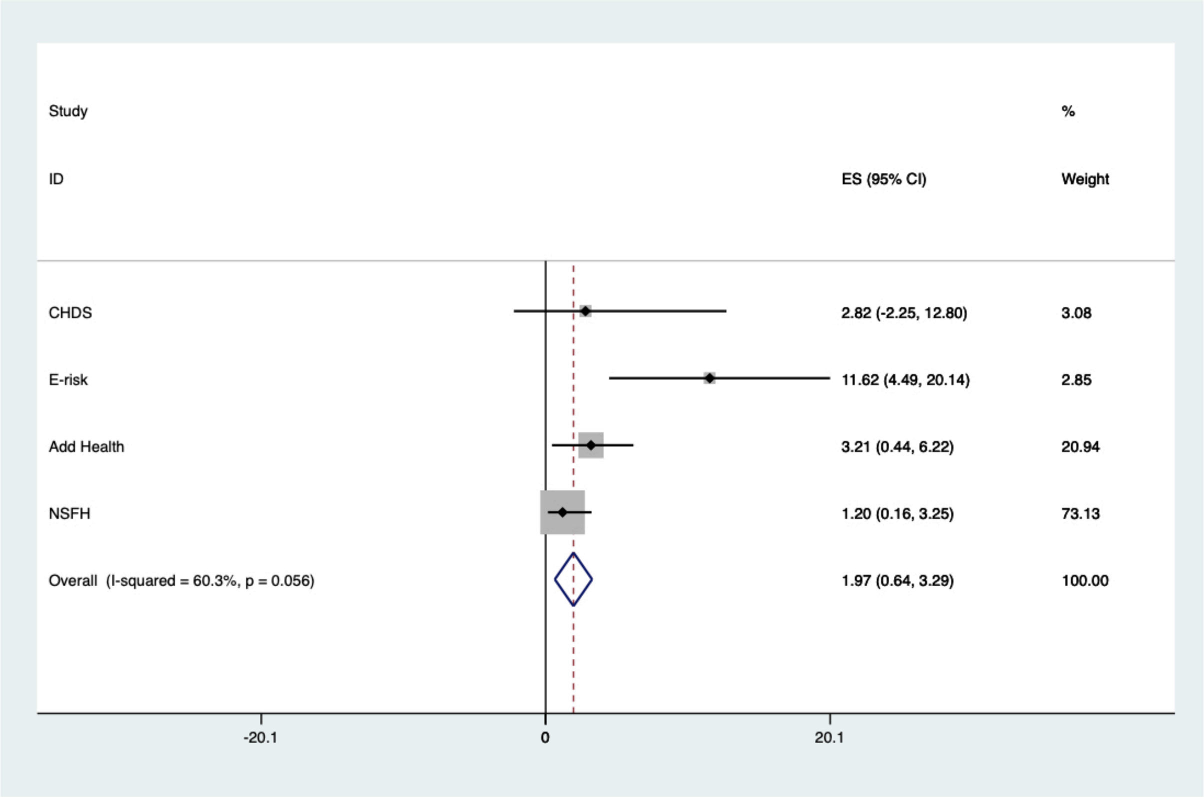

Figure 31 Results of fixed effects meta-analysis of the percentage differences between women with and without depression in past year severe physical IPV perpetration.

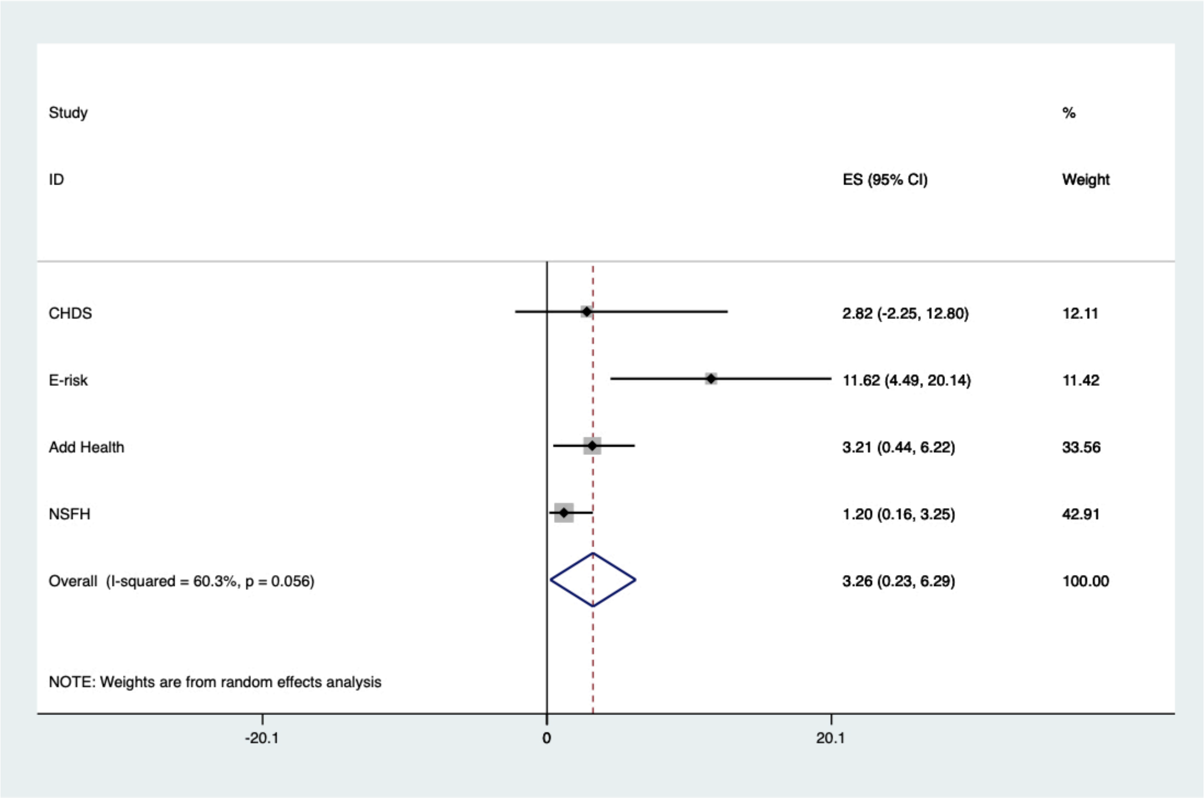

Figure 32 Results of random effects meta-analysis of the percentage differences between women with and without depression in past year severe physical IPV perpetration.

Indirect effect: In fixed effects meta-analysis, 1.2% (95% CI 0.6-1.8) of the total effect of depression on past year severe physical IPV perpetration among women was mediated by severe IPV victimisation. In random effects meta-analysis, the results were identical.

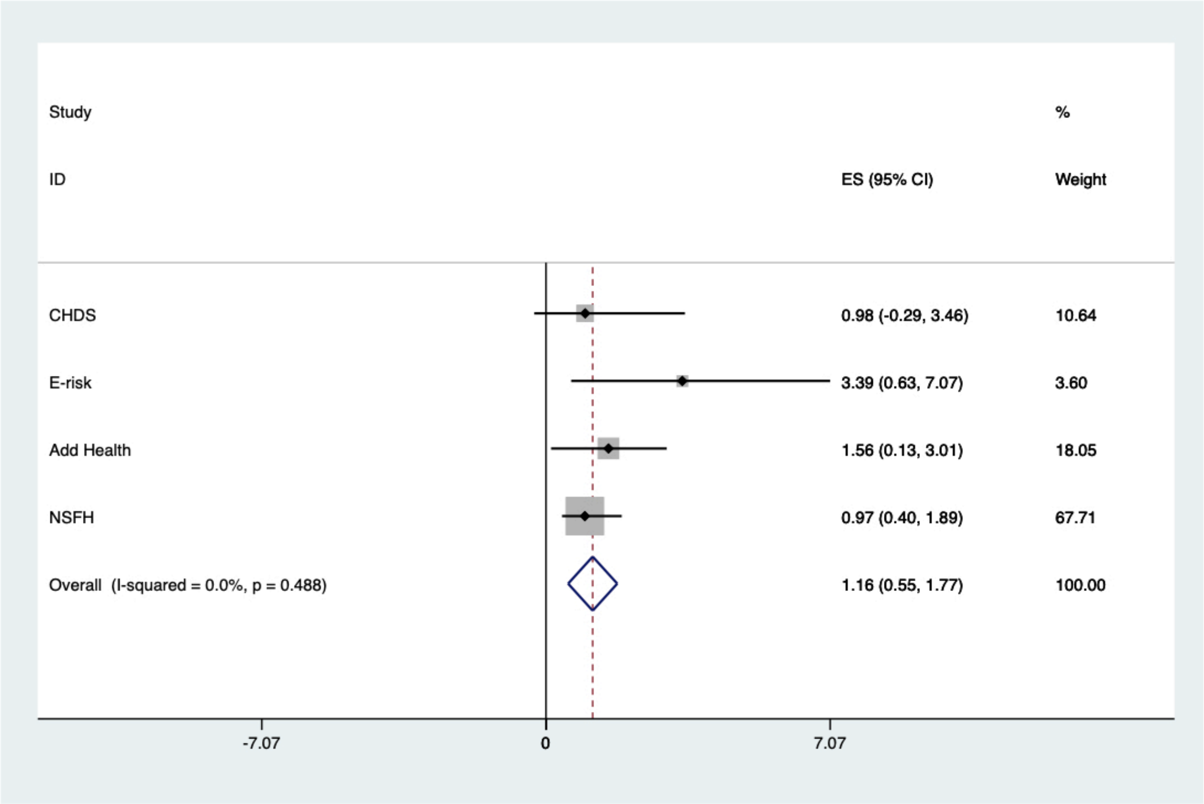

Figure 33 Results of fixed effects meta-analysis of the mediated percentage differences between women with and without depression in past year severe physical IPV perpetration.

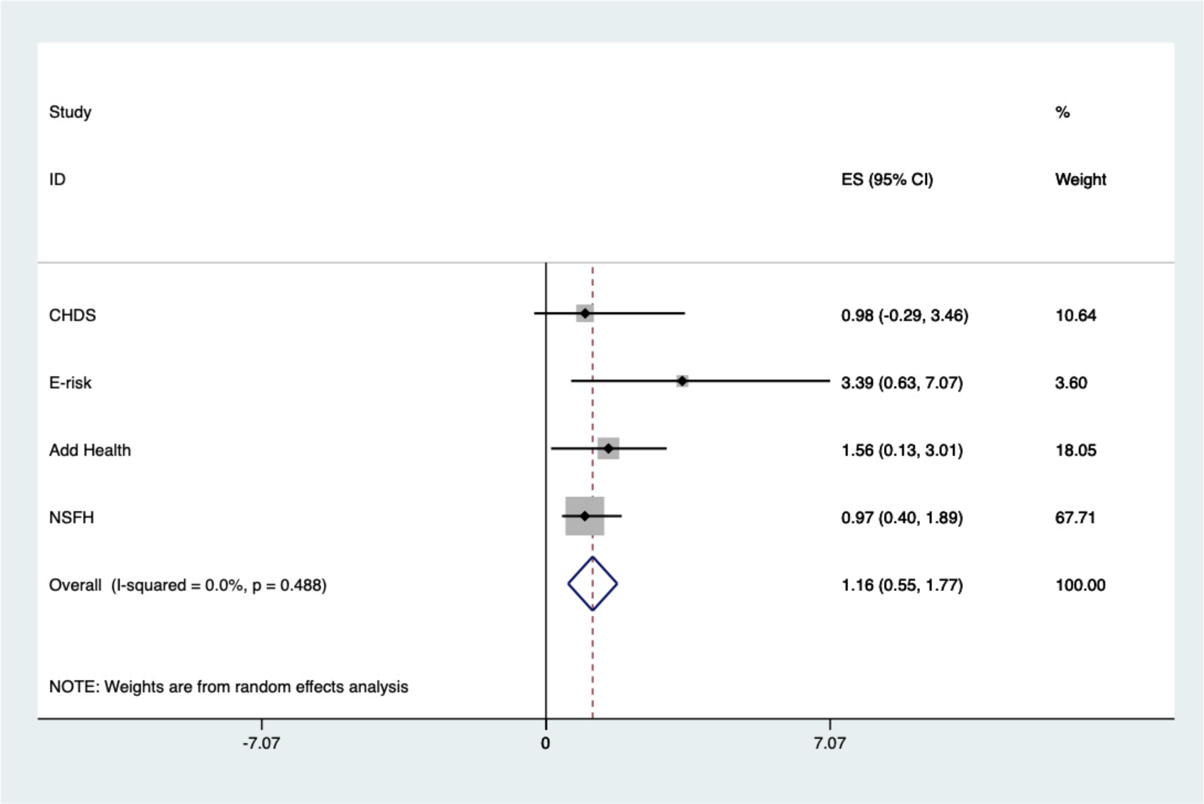

Figure 34 Results of random effects meta-analysis of the mediated percentage differences between women with and without depression in past year severe physical IPV perpetration.

Direct effect: In fixed effects meta-analysis, there was part of the total effect which was unmediated (0.9%, 95% CI -0.4-2.1). In random effects meta-analysis, there was no part of the total effect which was unmediated (1.6%, 95% CI -0.7-3.8).

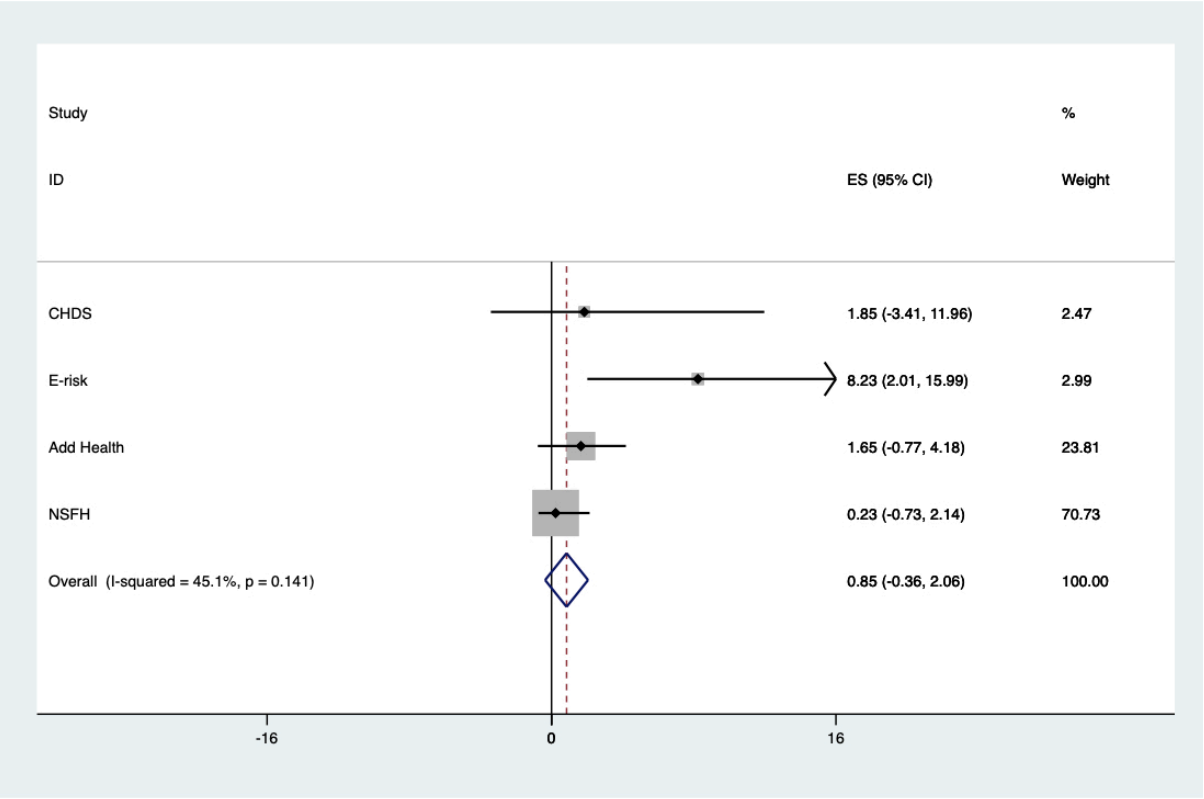

Figure 35 Results of fixed effects meta-analysis of the unmediated percentage differences between women with and without depression in past year severe physical IPV perpetration.

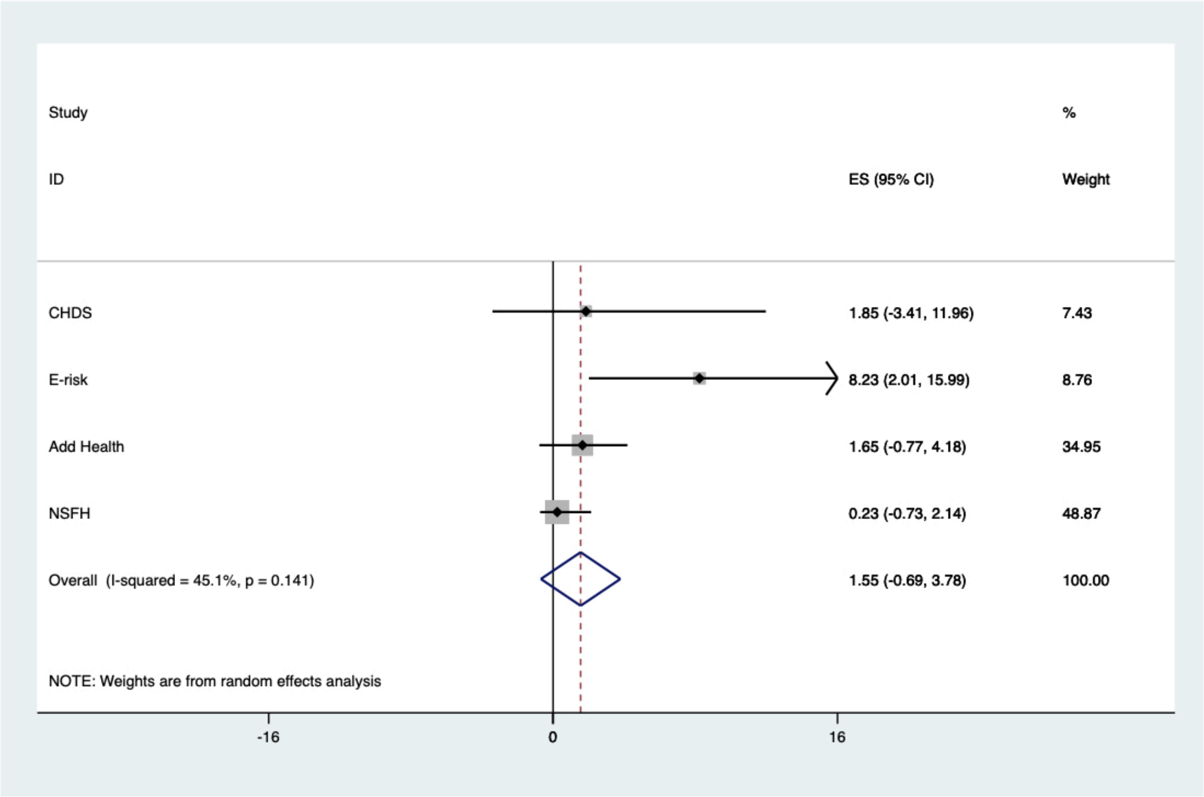

Figure 36 Results of random effects meta-analysis of the unmediated percentage differences between women with and without depression in past year severe physical IPV perpetration.
