# Supplementary material for: Past-year intimate partner violence perpetration among people with and without depression: an individual participant data (IPD) meta-mediation analysis
Source: Soc Psychiatry Psychiatr Epidemiol. 2021 Nov 29;58(12):1735–47. doi: 10.1007/s00127-021-02183-w (PMC10627935; doi:10.1007/s00127-021-02183-w)
Supplement: Supplementary file 3 — Supplementary file3 (PDF 377 KB) [file 127_2021_2183_MOESM3_ESM.pdf]

Supplementary Information

Article title:

Past year intimate partner violence perpetration among people with and without depression: an individual participant data (IPD) meta-mediation analysis.

Journal name:

Social Psychiatry and Psychiatric Epidemiology.

Author names:

Katherine Saunders, Sabine Landau, Louise M Howard, Helen L. Fisher, Louise Arseneault, Geraldine F H McLeod, Sian Oram

Corresponding author: Dr Sian Oram, King's College London, Health Service and Population Research Department, Institute of Psychiatry, Psychology & Neuroscience, London, United Kingdom; email: sian.oram@kcl.ac.uk

Search strategy: In stage 1, electronic searches were conducted of 7 bibliographic databases: [MEDLINE, EMBASE, PsycINFO, Web of Science, HMIC, BNID, and CINAHL]. Searches were conducted on 12/02/18 with no lower date limit. Search terms combined MeSH and keyword terms in three domains: intimate partner violence, perpetration, and mental disorder (see supplementary information), adapted from a previous systematic review [24]. Forward citation tracking (using Google Scholar and Web of Science) and reference list screening were used to identify other potentially relevant literature. Reference lists of relevant systematic reviews were searched to identify any other studies which potentially met the eligibility criteria. Five experts were contacted with a list of included studies for the aggregate systematic review and asked to identify any additional relevant studies or data for inclusion; one replied, identifying 13 studies, none of which were included in the final IPD meta-analyses. In stage 2, one academic referred us to an additional study. This study was screened, met inclusion criteria, and was included in the final IPD meta-analysis.

Screening: In stage 1, citations for the aggregate review were downloaded to Covidence© software on 12/02/18. Duplicates were removed. Titles and abstracts of the downloaded citations were assessed by KS for relevance to the current study based on the inclusion/exclusion criteria. A second reviewer independently assessed 10% of titles and abstracts (n=311), with initial agreement of 97%. Discrepancies were discussed and resolved. The full text of all citations deemed potentially relevant were obtained and assessed against inclusion/exclusion criteria for the aggregate review by KS. In stage 2, studies included in the aggregate review at the full-text stage were rescreened by KS against inclusion criteria for the IPD meta-analysis, which were more stringent. A second reviewer independently assessed 5% of randomly selected studies, with 100% agreement.

Search terms

1. Domestic violence/
2. Family violence/
3. Partner abuse/
4. Partner violence/
5. Spouse abuse/
6. Marital violence OR Marital abuse/
7. Stalk$/
8. Family conflict/
9. ((abus$ OR batter$ OR violen$ OR beat$) adj2 (perpetrat$ OR domestic OR partner$ OR family OR families OR spouse OR woman OR women OR men OR man OR female$ OR male$ OR wife OR wives OR husband$ OR boyfriend$ OR girlfriend$ OR brother$ OR sister$ OR father$ OR mother$ OR son$ OR daughter$).mp.)
10. (domestic adj2 homicid$).mp
11. 1 OR 2 OR 3 OR 4 OR 5 OR 6 OR 7 OR 8 OR 9 OR 10
12. Mental disorder/
13. Mental illness/
14. Mental health/
15. Mentally ill persons/
16. (Mental$ adj2 (problem$ OR difficult$ OR disorder$ OR ill$ OR health).mp.)
17. Mental health services/
18. Community Mental Health Services/
19. ((mental OR psychiatr$ OR psycholog$) adj2 (inpatient$ OR outpatient$ OR hospital$ OR clinic$ OR service$ OR ward$

OR healthcare).mp)
20. Autis$ OR ASD
21. Dementia OR Alzheimer OR cognitive impairment OR delirium
22. Attention deficit hyperactivity disorder OR ADHD
23. Schiz$/
24. Psychosis/
25. Psychotic/
26. Bipolar/
27. Depress$/
28. Mania OR manic
29. Neurosis OR psychoneurosis
30. Obsessive OR compulsive
31. Personality disorder/ OR anankastic personality disorder/ OR antisocial personality disorder/ OR avoidant personality disorder/ OR borderline personality disorder/ OR compulsive personality disorder/ OR dependent personality disorder/ OR histrionic personality disorder/ OR narcissistic personality disorder/ OR obsessive compulsive personality disorder/ OR paranoid personality disorder/ OR passive-aggressive personality disorder/ OR schizoid personality disorder/ OR schizotypal personality disorder/) OR ((anankastic OR asocial OR antisocial OR avoidant OR borderline OR dependent OR dissocial OR histrionic OR narcissistic OR obsessive OR compulsive OR paranoid OR passive-aggressive OR psychopath$ OR sadist$ OR sadomasochistic OR schizo$ OR sociopath$) adj person$).tw. OR (personality AND disorder$) OR psychopath$.tw OR sociopath$.tw
32. Eating disorder$/ OR Anorexia Nervosa/ OR Binge-Eating Disorder/or Bulimia Nervosa/ OR ((anorexi$ OR bulimi$) AND nervosa) OR eating disorder$ OR binge-eat$ OR (bing$ adj eat$) OR (compulsive adj (eat$ or vomit$ or purg$))
33. ((Delusional OR paranoi$ OR mood OR neurotic OR stress OR reactive OR combat OR somatoform OR somatization OR somatisation OR anxiety OR phobic OR obsessive-compulsive OR adjustment OR dissociat$) adj2 disorder$))
34. 13 OR 14 OR 15 OR 16 OR 17 OR 18 OR 19 OR 20 OR 21 OR 22 OR 23 OR 24 OR 25 OR 26 OR 27 OR 28 OR 29 OR 30 OR 31 OR 32 OR 33 OR 34
35. 11 AND 34
36. Perpetrat$/
37. 35 and 36

Supplementary Table 1

	**Exposure**	**Outcome**	**Mediators**	
Dataset	Depression	Past year physical IPV perpetration	Any past year IPV victimisation	Alcohol misuse
Christchurch Health and Development Study	DSM IV major depression diagnosis in the past year	‘grabbed or shook partner';<br>‘threw or tried to throw your partner bodily';<br>‘threw an object at partner';<br>‘choked or strangled partner';<br>‘kicked your partner';<br>‘became abusive after using drugs or alcohol';<br>'punched or hit your partner with something';<br>'slammed partner into a wall';<br>‘burned or scaled partner on purpose';<br>'beat partner up';<br>‘threatened partner with knife or gun';	cursed or swore at you';<br>shouted or yelled at you';<br>partner stomped off during disagreement';<br>deliberately said something to hurt you';<br>called you fat/ugly/unattractive';<br>destroyed something belonging to you';<br>accused you of being a lousy lover';<br>threatened to hit or throw something at you';<br>physically twisted your arm or hair';<br>pushed or shoved you';	Alcohol use disorder diagnosis in the past year

		'used knife or gun on partner';<br>'threatened to hit or throw something at partner';<br>'physically twisted partners arm or hair';<br>'pushed or shoved partner';<br>'slapped partner';<br>**Threshold – positive endorsement of any item**	slapped you';<br>force sex on you';<br>used threats to make you have sex';<br>grabbed or shook you';<br>threw or tried to throw you bodily';<br>threw an object at you';<br>choked or strangled you';<br>kicked you';<br>your partner became abusive after using drugs or alcohol';<br>punched or hit you with something';<br>slammed you into a wall';<br>burned or scaled you on purpose';<br>beat you up';<br>threatened you with knife or gun';<br>used knife or gun on you';<br>**Threshold – positive endorsement of any item**	
NSFH	On how many days during the past week did the participant:<br>Feel bothered by things that usually don't bother you?<br>Not feel like eating; your appetite was poor?<br>Feel that you could not shake off the blues even with help from your family or friends?<br>Have trouble keeping your mind on what you were doing?<br>Feel depressed?<br>Feel that everything you did was an effort?<br>Feel fearful?<br>Sleep restlessly?<br>Talk less than usual?<br>Feel lonely?<br>Feel sad?	Asked of those cohabiting:<br>During the past year, how many fights with your partner resulted in YOU hitting, shoving, or throwing things at him/her?<br>Has your PARTNER been cut, bruised, or seriously injured in a fight with you?<br><br>Asked of married participants:<br>During the past year, how many fights with your husband/wife resulted in YOU hitting, shoving, or throwing things at him/her?<br>Has your HUSBAND/WIFE been cut, bruised, or seriously injured in a fight with you?	Asked of those cohabiting:<br>During the past year, how many fights with your partner resulted in HIM/HER hitting, shoving, or throwing things at you?<br>Have YOU been cut, bruised, or seriously injured in a fight with your partner?<br><br>Asked of married participants:<br>During the past year, how many fights with your husband/wife resulted in HIM/HER hitting, shoving, or throwing things at you?<br>Have YOU been cut, bruised, or seriously injured in a fight with your husband/wife?<br>**Threshold – positive endorsement of any item**	Who living here has a problem of drinking too much alcohol?<br>**Threshold – if participant indicated they themselves had a problem with drinking too much alcohol.**

	Feel you could not get going?<br>**Threshold - Score of 12+**	**Threshold – positive endorsement of any item**		
Add Health	You were bothered by things that usually don't bother you.<br>(During the past seven days:) You could not shake off the blues, even with help from your family and your friends.<br>(During the past seven days:) You felt you were just as good as other people.<br>(During the past seven days:) You had trouble keeping your mind on what you were doing.<br>(During the past seven days:) You felt depressed.<br>(During the past seven days:) You felt that you were too tired to do things.<br>(During the past seven days:) You felt happy.<br>(During the past seven days:) You enjoyed life.<br>(During the past seven days:) You felt sad.<br>(During the past seven days:) You felt that people disliked you, during the past seven days.<br>**Threshold – Score of 8+**	How often (have/did) you threatened {partner} with violence, pushed or shoved (him/her), or thrown something at (him/her) that could hurt?<br>How often (have/did) you (slapped/slap), hit, or (kicked/kick) {partner}?<br>**Threshold – positive endorsement of any item**	How often (has/did) {initials} (threatened/threaten) you with violence, (pushed/push) or (shoved/shove) you, or (thrown/throw) something at you that could hurt?<br>H4RD19 - How often (has/did) {initials} (slapped/slap), hit or (kicked/kick) you?<br>H4RD21 - How often (has/did) {initials} (insisted/insist) on or (made/make) you have sexual relations with (him/her) when you didn't want to?<br>**Threshold – positive endorsement of any item**	During the past 30 days, on how many days did you drink?<br>Think of all the times you have had a drink during the past 30 days. How many drinks did you usually have each time? A 'drink' is a glass of wine, a can or bottle of beer, a wine cooler, a shot glass of liquor, or a mixed drink. (four or five drinks corresponds with a 'binge')<br><br>**Threshold – captured individuals who reported drinking 2 days per week or more (on average) in the past month AND usually drank 4 or more drinks per day.**

E-Risk	DIS used to measure past year depression among mothers	In the past year: Have you pushed, grabbed, or shoved a partner?<br>Have you slapped a partner?<br>Have you shaken a partner?<br>Have you thrown an object at a partner that could hurt them?<br>Have you kicked, bitten, or hit a partner with a fist?<br>Have you hit or tried to hit a partner with something?<br>Have you physically twisted a partner's arm?<br>Have you thrown or tried to throw a partner bodily?<br>Have you beaten a partner up (multiple blows)?<br>Have you choked or strangled a partner?<br>Have you threatened a partner with a knife or gun?<br>Have you used a knife or gun on a partner?<br>**Threshold – positive endorsement of any item**	In the past year: Has a partner pushed, grabbed, or shoved you?<br>Has a partner slapped you?<br>Has a partner shaken you?<br>Has a partner thrown an object at you that could hurt you?<br>Has a partner kicked, bitten, or hit you with a fist?<br>Has a partner hit or tried to hit you with something?<br>Has a partner physically twisted your arm?<br>Has a partner thrown or tried to throw you bodily?<br>Has a partner beaten you up (multiple blows)?<br>Has a partner choked or strangled you?<br>Has a partner threatened you with a knife or gun?<br>Has a partner used a knife or gun on you?<br>**Threshold – positive endorsement of any item**	Current substance misuse diagnosis
2014 APMS	CISR used to categorise mild, moderate and severe depression (ICD-10 diagnosis f32.2)	Pushed, held, pinned down, slapped a partner/ex in the past year.<br>Kicked, bit, hit, or thrown something at a partner/ex- partner that hurt them in past year<br>**Threshold – positive endorsement of any item**	Pushed, held, pinned down, slapped by a partner/ex in the past year.<br>Kicked, bit, hit, or had something thrown at you that hurt in past year<br>**Threshold – positive endorsement of any item**	**Threshold – score of 8+ on the audit**

PRISMA-IPD Checklist of items to include when reporting a systematic review and meta-analysis of individual participant data (IPD)

PRISMA-IPD Section/topic	Item No	Checklist item	Reported on page
**Title**			
Title	1	Identify the report as a systematic review and meta-analysis of individual participant data.	1
**Abstract**			
Structured summary	2	Provide a structured summary including as applicable:	2
		**Background**: state research question and main objectives, with information on participants, interventions, comparators and outcomes.	
		**Methods**: report eligibility criteria; data sources including dates of last bibliographic search or elicitation, noting that IPD were sought; methods of assessing risk of bias.	
		**Results**: provide number and type of studies and participants identified and number (%) obtained; summary effect estimates for main outcomes (benefits and harms) with confidence intervals and measures of statistical heterogeneity. Describe the direction and size of summary effects in terms meaningful to those who would put findings into practice.	
		**Discussion:** state main strengths and limitations of the evidence, general interpretation of the results and any important implications.	
		**Other:** report primary funding source, registration number and registry name for the systematic review and IPD meta-analysis.	
**Introduction**			
Rationale	3	Describe the rationale for the review in the context of what is already known.	3-4
Objectives	4	Provide an explicit statement of the questions being addressed with reference, as applicable, to participants, interventions, comparisons, outcomes and study design (PICOS). Include any hypotheses that relate to particular types of participant-level subgroups.	4
**Methods**			
Protocol and registration	5	Indicate if a protocol exists and where it can be accessed. If available, provide registration information including registration number and registry name. Provide publication details, if applicable.	4
Eligibility criteria	6	Specify inclusion and exclusion criteria including those relating to participants, interventions, comparisons, outcomes, study design and characteristics (e.g. years when conducted, required minimum follow-up). Note whether these were applied at the study or individual level i.e. whether eligible participants were included (and ineligible participants excluded) from a study that included a wider population than specified by the review inclusion criteria. The rationale for criteria should be stated.	4
Identifying studies -	7	Describe all methods of identifying published and unpublished studies including, as applicable: which bibliographic databases were searched with dates of coverage; details of any hand searching including of conference proceedings; use of study registers	4-5

information sources		and agency or company databases; contact with the original research team and experts in the field; open adverts and surveys. Give the date of last search or elicitation.	
Identifying studies - search	8	Present the full electronic search strategy for at least one database, including any limits used, such that it could be repeated.	Supp mat
Study selection processes	9	State the process for determining which studies were eligible for inclusion.	4-5
Data collection processes	10	Describe how IPD were requested, collected and managed, including any processes for querying and confirming data with investigators. If IPD were not sought from any eligible study, the reason for this should be stated (for each such study).<br>If applicable, describe how any studies for which IPD were not available were dealt with. This should include whether, how and what aggregate data were sought or extracted from study reports and publications (such as extracting data independently in duplicate) and any processes for obtaining and confirming these data with investigators.	4-5 and<br>Figure 1
Data items	11	Describe how the information and variables to be collected were chosen. List and define all study level and participant level data that were sought, including baseline and follow-up information. If applicable, describe methods of standardising or translating variables within the IPD datasets to ensure common scales or measurements across studies.	5-6 and supp mat
IPD integrity	A1	Describe what aspects of IPD were subject to data checking (such as sequence generation, data consistency and completeness, baseline imbalance) and how this was done.	5-6 and supp mat
Risk of bias assessment in individual studies.	12	Describe methods used to assess risk of bias in the individual studies and whether this was applied separately for each outcome. If applicable, describe how findings of IPD checking were used to inform the assessment. Report if and how risk of bias assessment was used in any data synthesis.	N/A
Specification of outcomes and effect measures	13	State all treatment comparisons of interests. State all outcomes addressed and define them in detail. State whether they were pre-specified for the review and, if applicable, whether they were primary/main or secondary/additional outcomes. Give the principal measures of effect (such as risk ratio, hazard ratio, difference in means) used for each outcome.	4-5
Synthesis methods	14	Describe the meta-analysis methods used to synthesise IPD. Specify any statistical methods and models used. Issues should include (but are not restricted to):<br>• Use of a one-stage or two-stage approach.<br>• How effect estimates were generated separately within each study and combined across studies (where applicable).<br>• Specification of one-stage models (where applicable) including how clustering of patients within studies was accounted for.<br>• Use of fixed or random effects models and any other model assumptions, such as proportional hazards.<br>• How (summary) survival curves were generated (where applicable).<br>• Methods for quantifying statistical heterogeneity (such as I^2 and τ^2).<br>• How studies providing IPD and not providing IPD were analysed together (where applicable).<br>• How missing data within the IPD were dealt with (where applicable).	5-7

Exploration of variation in effects	A2	If applicable, describe any methods used to explore variation in effects by study or participant level characteristics (such as estimation of interactions between effect and covariates). State all participant-level characteristics that were analysed as potential effect modifiers, and whether these were pre-specified.	4-7
Risk of bias across studies	15	Specify any assessment of risk of bias relating to the accumulated body of evidence, including any pertaining to not obtaining IPD for particular studies, outcomes or other variables.	Table 2
Additional analyses	16	Describe methods of any additional analyses, including sensitivity analyses. State which of these were pre-specified.	Supp mat
Results			
Study selection and IPD obtained	17	Give numbers of studies screened, assessed for eligibility, and included in the systematic review with reasons for exclusions at each stage. Indicate the number of studies and participants for which IPD were sought and for which IPD were obtained. For those studies where IPD were not available, give the numbers of studies and participants for which aggregate data were available. Report reasons for non-availability of IPD. Include a flow diagram.	Figure 1
Study characteristics	18	For each study, present information on key study and participant characteristics (such as description of interventions, numbers of participants, demographic data, unavailability of outcomes, funding source, and if applicable duration of follow-up). Provide (main) citations for each study. Where applicable, also report similar study characteristics for any studies not providing IPD.	Table 1 and Table 2
IPD integrity	A3	Report any important issues identified in checking IPD or state that there were none.	4-7
Risk of bias within studies	19	Present data on risk of bias assessments. If applicable, describe whether data checking led to the up-weighting or down-weighting of these assessments. Consider how any potential bias impacts on the robustness of meta-analysis conclusions.	None
Results of individual studies	20	For each comparison and for each main outcome (benefit or harm), for each individual study report the number of eligible participants for which data were obtained and show simple summary data for each intervention group (including, where applicable, the number of events), effect estimates and confidence intervals. These may be tabulated or included on a forest plot.	Table 3 and supp mat
Results of syntheses	21	Present summary effects for each meta-analysis undertaken, including confidence intervals and measures of statistical heterogeneity. State whether the analysis was pre-specified, and report the numbers of studies and participants and, where applicable, the number of events on which it is based.	Page 7-8, Figure 2, Table 3, Supp Mat
		When exploring variation in effects due to patient or study characteristics, present summary interaction estimates for each characteristic examined, including confidence intervals and measures of statistical heterogeneity. State whether the analysis was pre-specified. State whether any interaction is consistent across trials.	
		Provide a description of the direction and size of effect in terms meaningful to those who would put findings into practice.	
Risk of bias across studies	22	Present results of any assessment of risk of bias relating to the accumulated body of evidence, including any pertaining to the availability and representativeness of available studies, outcomes or other variables.	None

Additional analyses	23	Give results of any additional analyses (e.g. sensitivity analyses). If applicable, this should also include any analyses that incorporate aggregate data for studies that do not have IPD. If applicable, summarise the main meta-analysis results following the inclusion or exclusion of studies for which IPD were not available.	Supp mat
**Discussion**			
Summary of evidence	24	Summarise the main findings, including the strength of evidence for each main outcome.	8
Strengths and limitations	25	Discuss any important strengths and limitations of the evidence including the benefits of access to IPD and any limitations arising from IPD that were not available.	9-10
Conclusions	26	Provide a general interpretation of the findings in the context of other evidence.	8-9
Implications	A4	Consider relevance to key groups (such as policy makers, service providers and service users). Consider implications for future research.	10-11
**Funding**			
Funding	27	Describe sources of funding and other support (such as supply of IPD), and the role in the systematic review of those providing such support.	11-12

A1 – A3 denote new items that are additional to standard PRISMA items. A4 has been created as a result of re-arranging content of the standard PRISMA statement to suit the way that systematic review IPD meta-analyses are reported.

© Reproduced with permission of the PRISMA IPD Group, which encourages sharing and reuse for non-commercial purposes

Analysis Code

1. **E-Risk code:**

```
keep if a10who==1
*Variable generation*
/* Exposure variable:
mdepm10 - past year depression in mothers, measured using DIS - already binary
> DepressionPY
*/
gen DepressionPY = mdepm10
/*Outcome variable:*/
*Physical IPV perp
gen PhysicalIPVperpPY = vi2mm10
recode PhysicalIPVperpPY 0=0 1/max=1
label variable PhysicalIPVperpPY "Mother's past year perpetration of physical IPV"
label define PhysicalIPVperpPY 1 "Yes" 0 "No"
label values PhysicalIPVperpPY PhysicalIPVperpPY
tab PhysicalIPVperpPY vi2mm10

*Severe IPV perp
gen sev1=rp15m10
recode sev1 0=0 1/2=1 3/max=2 -8=3
gen sev2=rp17m10
recode sev2 0=0 1/2=1 3/max=2 -8=3
gen sev3=rp19m10
recode sev3 0=0 1/2=1 3/max=2 -8=3
gen sev4=rp21m10
recode sev4 0=0 1/2=1 3/max=2 -8=3
gen sev5=rp23m10
recode sev5 0=0 1/2=1 3/max=2 -8=3
gen sev6=rp25m10
recode sev6 0=0 1/2=1 3/max=2 -8=3
gen sev7=rp27m10
```

```
recode sev7 0=0 1/2=1 3/max=2 -8=3

gen sev8=rp29m10

recode sev8 0=0 1/2=1 3/max=2 -8=3


gen SevPhysIPVPerp=.

replace SevPhysIPVPerp=1 if sev1==1 | sev2==1 | sev3==1 | sev4==1 | sev5==1 | sev6==1 | sev7==1 | sev8==1

replace SevPhysIPVPerp=0 if sev1==0 & sev2==0 & sev3==0 & sev4==0 & sev5==0 & sev6==0 & sev7==0 &
sev8==0

label variable SevPhysIPVPerp "Perpetration of severe physical IPV in the past year"

label define SevPhysIPVPerp 0 "No" 1 "Yes"

label values SevPhysIPVPerp SevPhysIPVPerp

tab SevPhysIPVPerp

tab SevPhysIPVPerp sev1

tab SevPhysIPVPerp sev2

tab SevPhysIPVPerp sev3

tab SevPhysIPVPerp sev4

tab SevPhysIPVPerp sev5

tab SevPhysIPVPerp sev6

tab SevPhysIPVPerp sev7

tab SevPhysIPVPerp sev8


/*Mediator variables:*/


*alcohol misuse

gen SubMisuse = alcdxmm10


*IPV victimisation in the past year

gen IPVvictPY = viogpm10

recode IPVvictPY 0=0 1/max=1

label variable IPVvictPY "Mother's past year physical IPV victimisation"

label define IPVvictPY 1 "Yes" 0 "No"

label values IPVvictPY IPVvictPY

tab IPVvictPY viogpm10


*severe IPV victimisation in the past year
```

```
gen sev1v=rp16m10

recode sev1v 0=0 1/2=1 3/max=2 -8=3

gen sev2v=rp18m10

recode sev2v 0=0 1/2=1 3/max=2 -8=3

gen sev3v=rp20m10

recode sev3v 0=0 1/2=1 3/max=2 -8=3

gen sev4v=rp22m10

recode sev4v 0=0 1/2=1 3/max=2 -8=3

gen sev5v=rp24m10

recode sev5v 0=0 1/2=1 3/max=2 -8=3

gen sev6v=rp26m10

recode sev6v 0=0 1/2=1 3/max=2 -8=3

gen sev7v=rp28m10

recode sev7v 0=0 1/2=1 3/max=2 -8=3

gen sev8v=rp30m10

recode sev8v 0=0 1/2=1 3/max=2 -8=3


gen SevPhysIPVvict=.

replace SevPhysIPVvict=1 if sev1v==1 | sev2v==1 | sev3v==1 | sev4v==1 | sev5v==1 | sev6v==1 | sev7v==1 | sev8v==1

replace SevPhysIPVvict=0 if sev1v==0 & sev2v==0 & sev3v==0 & sev4v==0 & sev5v==0 & sev6v==0 & sev7v==0 & sev8v==0

label variable SevPhysIPVvict "Severe physical IPV victimisation in the past year"

label define SevPhysIPVvict 0 "No" 1 "Yes"

label values SevPhysIPVvict SevPhysIPVvict

tab SevPhysIPVvict

tab SevPhysIPVvict sev1v

tab SevPhysIPVvict sev2v

tab SevPhysIPVvict sev3v

tab SevPhysIPVvict sev4v

tab SevPhysIPVvict sev5v

tab SevPhysIPVvict sev6v

tab SevPhysIPVvict sev7v

tab SevPhysIPVvict sev8v
```

```
/*Variables for adjustment + descriptive analyses:*/
*education
gen education=hiedm5
replace education=hiedm7 if hiedm7>hiedm5
replace education=hiedm5 if hiedm7==.
recode education -9=. -1=. 0=0 1/3=1 4/7=2
label define education 0 "None" 1 "High school qualifications" 2 "Degree/equiv or higher"
label values education education
label variable education "Highest education qualification at child age 7"
tab education hiedgm57
tab education, gen (educationdummy)

*number of children
gen childrenbinary=KidsinFamily10
recode childrenbinary 0=0 1/max=1
label variable childrenbinary "Has children in family Y/N"
label define childrenbinary 0 "No" 1 "Yes"
label values childrenbinary childrenbinary
tab childrenbinary KidsinFamily10

*age
gen age = mage10

*relationship status
rename rp31m10 MumPartneredLast5y
tab MumPartneredLast5y
gen relationshipbinary=MumPartneredLast5y
label variable relationshipbinary "Has mum had a partner in past 5 years?"
label define relationshipbinary 0 "No" 1 "Yes"
label values relationshipbinary relationshipbinary
tab relationshipbinary

*income
tab ed56m5
```

```
gen income=ed56m5

recode income 99=. 0=1999.5 1=4999.5 2=6999.5 3=8999.5 4=10999.5 5=13499.5 6=16499.5 7=18999.5 8=21499.5 9=24499.5 10=27499.5 11=27499.5 12=33499.5 13=36499.5 14=39499.5 15=42499.5

label variable income "Approximate income"


*ANALYSIS

** descriptive

summarize mage10, detail

* descriptive

tab DepressionPY

proportion DepressionPY

* descriptive

tab PhysicalIPVperpPY

proportion PhysicalIPVperpPY

* descriptive

tab SevPhysIPVPerp

proportion SevPhysIPVPerp

* descriptive

tab2 SevPhysIPVPerp DepressionPY

proportion SevPhysIPVPerp if DepressionPY==1

proportion SevPhysIPVPerp if DepressionPY==0

* descriptive

tab2 PhysicalIPVperpPY DepressionPY

proportion PhysicalIPVperpPY if DepressionPY==1

proportion PhysicalIPVperpPY if DepressionPY==0



/* mediation analyses */

*** physical perpetration outcome analyses ***

*alcohol mediation

gen int_dep_substance =  DepressionPY*SubMisuse

medeff (logit SubMisuse DepressionPY age income educationdummy1 educationdummy2 KidsinFamily10) (logit PhysicalIPVperpPY DepressionPY SubMisuse int_dep_substance age income educationdummy1 educationdummy2 KidsinFamily10), mediate (SubMisuse) treat(DepressionPY) sims(1000) seed(1) level(95) interact(int_dep_substance)
```

/*IPV vict mediation */

gen int_dep_vict = DepressionPY*IPVvictPY

medeff (logit IPVvictPY DepressionPY age income educationdummy1 educationdummy2 KidsinFamily10) (logit PhysicalIPVperpPY DepressionPY IPVvictPY int_dep_vict age income educationdummy1 educationdummy2 KidsinFamily10), mediate (IPVvictPY) treat(DepressionPY) sims(1000) seed(1) level(95) interact(int_dep_vict)

/*severe IPV vict mediation*/

gen int_dep_Sevvict = DepressionPY*SevPhysIPVvict

medeff (logit SevPhysIPVvict DepressionPY age income educationdummy1 educationdummy2 KidsinFamily10) (logit PhysicalIPVperpPY DepressionPY SevPhysIPVvict int_dep_Sevvict age income educationdummy1 educationdummy2 KidsinFamily10), mediate (SevPhysIPVvict) treat(DepressionPY) sims(1000) seed(1) level(95) interact(int_dep_Sevvict)

*** severe physical perpetration outcome analyses ***

/*IPV vict mediation */

medeff (logit IPVvictPY DepressionPY age income educationdummy1 educationdummy2 KidsinFamily10) (logit SevPhysIPVPerp DepressionPY IPVvictPY int_dep_vict age income educationdummy1 educationdummy2 KidsinFamily10), mediate (IPVvictPY) treat(DepressionPY) sims(1000) seed(1) level(95) interact(int_dep_vict)

/*severe IPV vict mediation */

medeff (logit SevPhysIPVvict DepressionPY age income educationdummy1 educationdummy2 KidsinFamily10) (logit SevPhysIPVPerp DepressionPY SevPhysIPVvict int_dep_Sevvict age income educationdummy1 educationdummy2 KidsinFamily10), mediate (SevPhysIPVvict) treat(DepressionPY) sims(1000) seed(1) level(95) interact(int_dep_Sevvict)

2. CHDS code

/*Exposure variables:*/

rename d30mdy majordepPY

label variable majordepPY "Major depression in the past year"

label define majordepPY 0 "No" 1 "Yes"

label values majordepPY majordepPY

/*Outcome variables:*/

gen perp1=z1531

recode perp1 0=0 1/7=1 8/max=2

gen perp2=z1532

```
recode perp2 0=0 1/7=1 8/max=2

gen perp3=z1533

recode perp3 0=0 1/7=1 8/max=2

gen perp4=z1534

recode perp4 0=0 1/7=1 8/max=2

gen perp5=z1535

recode perp5 0=0 1/7=1 8/max=2

gen perp6=z1536

recode perp6 0=0 1/7=1 8/max=2

gen perp7=z1537

recode perp7 0=0 1/7=1 8/max=2

gen perp8=z1538

recode perp8 0=0 1/7=1 8/max=2

gen perp9=z1539

recode perp9 0=0 1/7=1 8/max=2

gen perp10=z1540

recode perp10 0=0 1/7=1 8/max=2

gen perp11=z1541

recode perp11 0=0 1/7=1 8/max=2

gen perp12=z1542

recode perp12 0=0 1/7=1 8/max=2

gen perp13=z1526

recode perp13 0=0 1/7=1 8/max=2

gen perp14=z1527

recode perp14 0=0 1/7=1 8/max=2

gen perp15=z1528

recode perp15 0=0 1/7=1 8/max=2

gen PhysicalIPVperpPY =.

replace PhysicalIPVperpPY = 1 if perp1 == 1 | perp2 == 1 | perp3 == 1 | perp4 == 1 | perp5 == 1 | perp6 == 1 | perp7 == 1 | perp8 == 1 | perp9 == 1 | perp10 == 1 | perp11 == 1 | perp12 == 1 | perp13 == 1 | perp14 == 1 | perp15 == 1

replace PhysicalIPVperpPY = 0 if perp1 == 0 & perp2 == 0 & perp3 == 0 & perp4 == 0 & perp5 == 0 & perp6 == 0 & perp7 == 0 & perp8 == 0 & perp9 == 0 & perp10 == 0 & perp11 == 0 & perp12 == 0 & perp13 == 0 & perp14 == 0 & perp15 == 0

label variable PhysicalIPVperpPY "Perpetration of any physical IPV in the past year"

label define PhysicalIPVperpPY 0 "No" 1 "Yes"
```

```
label values PhysicalIPVperpPY PhysicalIPVperpPY

tab PhysicalIPVperpPY, missing

tab PhysicalIPVperpPY z1531, missing

tab PhysicalIPVperpPY z1532, missing

tab PhysicalIPVperpPY z1533, missing

tab PhysicalIPVperpPY z1534, missing

tab PhysicalIPVperpPY z1535, missing

tab PhysicalIPVperpPY z1536, missing

tab PhysicalIPVperpPY z1537, missing

tab PhysicalIPVperpPY z1538, missing

tab PhysicalIPVperpPY z1539, missing

tab PhysicalIPVperpPY z1540, missing

tab PhysicalIPVperpPY z1541, missing

tab PhysicalIPVperpPY z1542, missing

tab PhysicalIPVperpPY z1526, missing

tab PhysicalIPVperpPY z1527, missing

tab PhysicalIPVperpPY z1528, missing


/* Severe physical IPV perp PY*/

gen SevPhysIPVPerp = .

replace SevPhysIPVPerp = 1 if perp2==1 | perp4 == 1 | perp5 == 1 | perp7 == 1 | perp8 == 1 | perp9 == 1 |
perp10==1 | perp11 == 1 | perp12 == 1

replace SevPhysIPVPerp = 0 if perp2==0 & perp4 == 0 & perp5 == 0 & perp7 == 0 & perp8 == 0 & perp9 == 0 &
perp10==0 & perp11 == 0 & perp12 == 0

label variable SevPhysIPVPerp "Perpetration of severe physical IPV in the past year"

label define SevPhysIPVPerp 0 "No" 1 "Yes"

label values SevPhysIPVPerp SevPhysIPVPerp

tab SevPhysIPVPerp

tab SevPhysIPVPerp z1532

tab SevPhysIPVPerp z1534

tab SevPhysIPVPerp z1535

tab SevPhysIPVPerp z1537

tab SevPhysIPVPerp z1538

tab SevPhysIPVPerp z1539

tab SevPhysIPVPerp z1540
```

```
tab SevPhysIPVPerp z1541
tab SevPhysIPVPerp z1542

/*Mediator variables:*/

gen vict1=z1482
recode vict1 0=0 1/7=1 8/max=2
gen vict2=z1483
recode vict2 0=0 1/7=1 8/max=2
gen vict3=z1484
recode vict3 0=0 1/7=1 8/max=2
gen vict4=z1485
recode vict4 0=0 1/7=1 8/max=2
gen vict5=z1486
recode vict5 0=0 1/7=1 8/max=2
gen vict6=z1487
recode vict6 0=0 1/7=1 8/max=2
gen vict7=z1488
recode vict7 0=0 1/7=1 8/max=2
gen vict8=z1489
recode vict8 0=0 1/7=1 8/max=2
gen vict9=z1490
recode vict9 0=0 1/7=1 8/max=2
gen vict10=z1491
recode vict10 0=0 1/7=1 8/max=2
gen vict11=z1492
recode vict11 0=0 1/7=1 8/max=2
gen vict12=z1493
recode vict12 0=0 1/7=1 8/max=2
gen vict13=z1494
recode vict13 0=0 1/7=1 8/max=2
gen vict14=z1495
recode vict14 0=0 1/7=1 8/max=2
gen vict15=z1496
```

recode vict15 0=0 1/7=1 8/max=2

gen vict16=z1497

recode vict16 0=0 1/7=1 8/max=2

gen vict17=z1498

recode vict17 0=0 1/7=1 8/max=2

gen vict18=z1499

recode vict18 0=0 1/7=1 8/max=2

gen vict19=z1500

recode vict19 0=0 1/7=1 8/max=2

gen vict20=z1501

recode vict20 0=0 1/7=1 8/max=2

gen vict21=z1502

recode vict21 0=0 1/7=1 8/max=2

gen vict22=z1503

recode vict22 0=0 1/7=1 8/max=2

gen vict23=z1504

recode vict23 0=0 1/7=1 8/max=2

gen vict24=z1505

recode vict24 0=0 1/7=1 8/max=2

gen vict25=z1506

recode vict25 0=0 1/7=1 8/max=2

gen IPVvictPY = .

replace IPVvictPY = 1 if vict1==1 | vict2 ==1 | vict3==1 | vict4 ==1 | vict5==1 |vict6 ==1 | vict7==1 | vict8==1 | vict9 ==1 | vict10==1 | vict11==1 | vict12==1 | vict13==1 |vict14==1 | vict15==1 | vict16==1 | vict17==1 | vict18==1 | vict19==1 | vict20==1 | vict21==1 | vict22==1 | vict23==1 | vict24==1 | vict25==1

replace IPVvictPY = 0 if vict1==0 & vict2 ==0 & vict3==0 & vict4 ==0 & vict5==0 & vict6 ==0 & vict7==0 & vict8==0 & vict9 ==0 & vict10==0 & vict11==0 & vict12==0 & vict13==0 & vict14==0 & vict15==0 & vict16==0 & vict17==0 & vict18==0 & vict19==0 & vict20==0 & vict21==0 & vict22==0 & vict23==0 & vict24==0 & vict25==0

label variable IPVvictPY "Any past year IPV victimisation"

label define IPVvictPY 0 "No" 1 "Yes"

label values IPVvictPY IPVvictPY

tab IPVvictPY

tab IPVvictPY z1482, missing

tab IPVvictPY z1483, missing

tab IPVvictPY z1484, missing

```
tab IPVvictPY z1485, missing
tab IPVvictPY z1486, missing
tab IPVvictPY z1487, missing
tab IPVvictPY z1488, missing
tab IPVvictPY z1489, missing
tab IPVvictPY z1490, missing
tab IPVvictPY z1491, missing
tab IPVvictPY z1492, missing
tab IPVvictPY z1493, missing
tab IPVvictPY z1494, missing
tab IPVvictPY z1495, missing
tab IPVvictPY z1496, missing
tab IPVvictPY z1497, missing
tab IPVvictPY z1498, missing
tab IPVvictPY z1499, missing
tab IPVvictPY z1500, missing
tab IPVvictPY z1501, missing
tab IPVvictPY z1502, missing
tab IPVvictPY z1503, missing
tab IPVvictPY z1504, missing
tab IPVvictPY z1505, missing
tab IPVvictPY z1506, missing


gen SevPhysIPVvict= .
replace SevPhysIPVvict = 1 if  vict15==1 | vict17==1 | vict18==1 | vict20==1 | vict21==1 | vict22==1 | vict23==1 | vict24==1 | vict25==1
replace SevPhysIPVvict = 0 if vict15==0 & vict17==0 & vict18==0 & vict20==0 & vict21==0 & vict22==0 & vict23==0 & vict24==0 & vict25==0
label variable SevPhysIPVvict "Any severe IPV victimisation in past year"
label define SevPhysIPVvict 0 "No" 1 "Yes"
label values SevPhysIPVvict SevPhysIPVvict
tab SevPhysIPVvict
tab SevPhysIPVvict vict15
tab SevPhysIPVvict vict17
tab SevPhysIPVvict vict18
```

```
tab SevPhysIPVvict vict20

tab SevPhysIPVvict vict21

tab SevPhysIPVvict vict22

tab SevPhysIPVvict vict23

tab SevPhysIPVvict vict24

tab SevPhysIPVvict vict25

label define alcud30 0 "No" 1 "Yes"

label values alcud30 alcud30

 rename alcud30 AUDPY


/*Variables for adjustment + descriptive analyses:*/

gen income=ppptotfaminc30

label define gender 1 "Male" 2 "Female"

label values gender gender

rename gender sexgender

label define hieduc30 1 "None" 2 "High school qualifications" 3 "Trade/technical qualifications" 4 "Tertiary (University/Polytech Degree) qualifications"

label values hieduc30 hieduc30


gen education=hieduc30

recode education 1=0 2=1 3/4=2

label variable education "Highest education qualification"

label define education 0 "None" 1 "High school qualifications/equivalent" 2 "Degree/equiv"

label values education education

tab hieduc30 education

tab education, gen (educationdummy)


** descriptive

tab majordepPY

tab PhysicalIPVperpPY

tab SevPhysIPVPerp

tab PhysicalIPVperpPY sexgender

tab SevPhysIPVPerp sexgender

tab PhysicalIPVperpPY majordepPY if sexgender==1

tab PhysicalIPVperpPY majordepPY if sexgender==2
```

tab SevPhysIPVPerp majordepPY if sexgender==1

tab SevPhysIPVPerp majordepPY if sexgender==2

*** mediation - physical IPV perp

/* men physical IPV perp - alcohol use mediation analysis*/

gen int_dep_alcohol = majordepPY*AUDPY

medeff (logit AUDPY majordepPY income z10 educationdummy1 educationdummy2) (logit PhysicalIPVperpPY majordepPY AUDPY int_dep_alcohol income z10 educationdummy1 educationdummy2) if sexgender==1, mediate (AUDPY) treat(majordepPY) sims(1000) seed(1) level(95) interact(int_dep_alcohol)

/* women - physical IPV perp - alcohol use mediation analysis*/

medeff (logit AUDPY majordepPY income z10 educationdummy1 educationdummy2) (logit PhysicalIPVperpPY majordepPY AUDPY income z10 educationdummy1 educationdummy2) if sexgender==2, mediate (AUDPY) treat(majordepPY) sims(1000) seed(1) level(95)

***

/* men - physical IPV perp - IPV victimisation mediation analysis*/

gen int_dep_ipvvict = majordepPY*IPVvictPY

*men - physical IPV perp - IPV victimisation mediation analysis

medeff (logit IPVvictPY majordepPY income z10 educationdummy1 educationdummy2) (logit PhysicalIPVperpPY majordepPY IPVvictPY int_dep_ipvvict income childrenbinary educationdummy1 educationdummy2) if sexgender==1, mediate (IPVvictPY) treat(majordepPY) sims(1000) seed(1) level(95) interact(int_dep_ipvvict)

/* women - physical IPV perp - IPV victimisation mediation analysis*/

medeff (logit IPVvictPY majordepPY income z10 educationdummy1 educationdummy2) (logit PhysicalIPVperpPY majordepPY IPVvictPY int_dep_ipvvict income z10 educationdummy1 educationdummy2) if sexgender==2, mediate (IPVvictPY) treat(majordepPY) sims(1000) seed(1) level(95) interact(int_dep_ipvvict)

***

/* men - physical IPV perp - severe IPV victimisation mediation analysis*/

gen int_dep_sevipvvict = majordepPY*SevPhysIPVvict

medeff (logit SevPhysIPVvict majordepPY income z10 educationdummy1 educationdummy2) (logit PhysicalIPVperpPY majordepPY SevPhysIPVvict int_dep_sevipvvict income z10 educationdummy1 educationdummy2) if sexgender==1, mediate (SevPhysIPVvict) treat(majordepPY) sims(1000) seed(1) level(95) interact(int_dep_sevipvvict)

/* women - physical IPV perp - severe IPV victimisation mediation analysis*/

medeff (logit SevPhysIPVvict majordepPY income z10 educationdummy1 educationdummy2) (logit PhysicalIPVperpPY majordepPY SevPhysIPVvict int_dep_sevipvvict income z10 educationdummy1 educationdummy2) if sexgender==2, mediate (SevPhysIPVvict) treat(majordepPY) sims(1000) seed(1) level(95) interact(int_dep_sevipvvict)

**** severe IPV perpetration ***

/* men sev physical IPV perp - alcohol use mediation analysis*/

medeff (logit AUDPY majordepPY income z10 educationdummy1 educationdummy2) (logit SevPhysIPVPerp majordepPY AUDPY int_dep_alcohol income z10 educationdummy1 educationdummy2) if sexgender==1, mediate (AUDPY) treat(majordepPY) sims(1000) seed(1) level(95) interact(int_dep_alcohol)

/* women - sev physical IPV perp - alcohol use mediation analysis*/

medeff (logit AUDPY majordepPY income z10 educationdummy1 educationdummy2) (logit SevPhysIPVPerp majordepPY AUDPY int_dep_alcohol income z10 educationdummy1 educationdummy2) if sexgender==2, mediate (AUDPY) treat(majordepPY) sims(1000) seed(1) level(95) interact(int_dep_alcohol)

***

/* men - sev physical IPV perp - IPV victimisation - mediation analysis*/

medeff (logit IPVvictPY majordepPY income z10 educationdummy1 educationdummy2) (logit SevPhysIPVPerp majordepPY IPVvictPY int_dep_ipvvict income z10 educationdummy1 educationdummy2) if sexgender==1, mediate (IPVvictPY) treat(majordepPY) sims(1000) seed(1) level(95) interact(int_dep_ipvvict)

/* women - sev physical IPV perp - IPV victimisation - mediation analysis*/

medeff (logit IPVvictPY majordepPY income z10 educationdummy1 educationdummy2) (logit SevPhysIPVPerp majordepPY IPVvictPY int_dep_ipvvict income z10 educationdummy1 educationdummy2) if sexgender==2, mediate (IPVvictPY) treat(majordepPY) sims(1000) seed(1) level(95) interact(int_dep_ipvvict)

***

/* men - sev physical IPV perp - severe IPV victimisation - mediation analysis*/

medeff (logit SevPhysIPVvict majordepPY income z10 educationdummy1 educationdummy2) (logit SevPhysIPVPerp majordepPY SevPhysIPVvict int_dep_sevipvvict income z10 educationdummy1 educationdummy2) if sexgender==1, mediate (SevPhysIPVvict) treat(majordepPY) sims(1000) seed(1) level(95) interact(int_dep_sevipvvict)

/* women - sev physical IPV perp - severe IPV victimisation - mediation analysis*/

medeff (logit SevPhysIPVvict majordepPY income z10 educationdummy1 educationdummy2) (logit SevPhysIPVPerp majordepPY SevPhysIPVvict int_dep_sevipvvict income z10 educationdummy1 educationdummy2) if sexgender==2, mediate (SevPhysIPVvict) treat(majordepPY) sims(1000) seed(1) level(95) interact(int_dep_sevipvvict)

3. NSFH code

keep if ASAMPLE==1

gen relationship=MARCOHAB

recode relationship 1/6=1 7/max=.

drop if relationship==.

svyset [pweight=SAMWT]

rename M2DP01 sexgender

/*exposure*/

recode E202A 0=0 1/2=1 3/4=2 5/7=3 8/max=.

tab E202A

```
recode E202B 0=0 1/2=1 3/4=2 5/7=3 8/max=.
tab E202B
recode E202C 0=0 1/2=1 3/4=2 5/7=3 8/max=.
tab E202C
recode E202D 0=0 1/2=1 3/4=2 5/7=3 8/max=.
tab E202D
recode E202E 0=0 1/2=1 3/4=2 5/7=3 8/max=.
tab E202E
recode E202F 0=0 1/2=1 3/4=2 5/7=3 8/max=.
tab E202F
recode E202G 0=0 1/2=1 3/4=2 5/7=3 8/max=.
tab E202G
recode E202H 0=0 1/2=1 3/4=2 5/7=3 8/max=.
tab E202H
recode E202I 0=0 1/2=1 3/4=2 5/7=3 8/max=.
tab E202I
recode E202J 0=0 1/2=1 3/4=2 5/7=3 8/max=.
tab E202J
recode E202K 0=0 1/2=1 3/4=2 5/7=3 8/max=.
tab E202K
recode E202L 0=0 1/2=1 3/4=2 5/7=3 8/max=.
tab E202L

gen CESDadapt= E202A+ E202B+ E202C+ E202D+ E202E+ E202F+ E202G+ E202H+ E202I+ E202J+ E202K+ E202L
tab CESDadapt

gen CESDbinary=CESDadapt
recode CESDbinary min/11=0 12/max=1
label variable CESDbinary "Depression score of 12+ on adapted CESD"
label define CESDbinary 0 "No" 1 "Yes"
label values CESDbinary CESDbinary
tab CESDadapt CESDbinary
tab2 CESDbinary sexgender
```

```
/* outcome*/

gen physIPVperp1=E613

recode physIPVperp1 0=0 1/4=1 5/max=.

label variable physIPVperp1 "Had a physical fight in the past year which has resulted in the respondent hitting, shoving, or throwing things at their partner"

label define physIPVperp1 0 "No" 1 "Yes"

label values physIPVperp1 physIPVperp1

tab physIPVperp1 E613


gen physIPVperp2=E616

recode physIPVperp2 1=1 2=0 3/max=.

label variable physIPVperp2 "Partner been cut, brusied or seriously injured in a fight with the respondent"

label define physIPVperp2 0 "No" 1 "Yes"

label values physIPVperp2 physIPVperp2

tab physIPVperp2 E616


gen physIPVperp3=E709

recode physIPVperp3 0=0 1/4=1 6/max=.

label variable physIPVperp3 "Had a physical fight in the past year which has resulted in the respondent hitting, shoving, or throwing things at their partner"

label define physIPVperp3 0 "No" 1 "Yes"

label values physIPVperp3 physIPVperp3

tab physIPVperp3 E709


gen physIPVperp4=E712

recode physIPVperp4 1=1 2=0 3/max=.

label variable physIPVperp4 "Partner been cut, brusied or seriously injured in a fight with the respondent"

label define physIPVperp4 0 "No" 1 "Yes"

label values physIPVperp4 physIPVperp4

tab physIPVperp4 E712


gen PhysicalIPVperpPY =.

replace PhysicalIPVperpPY =0 if E708==2

replace PhysicalIPVperpPY =0 if E612==2
```

```
replace PhysicalIPVperpPY =1 if physIPVperp1==1 | physIPVperp2==1 | physIPVperp3==1 | physIPVperp4==1
label variable PhysicalIPVperpPY  "Any physical or injurious IPV perpetration in the past year"
label define PhysicalIPVperpPY  0 "No" 1 "Yes"
label values PhysicalIPVperpPY  PhysicalIPVperpPY
tab PhysicalIPVperpPY

 /*Severe IPV perpetration*/
gen SevPhysIPVPerp=PhysicalIPVperpPY
replace SevPhysIPVPerp=3 if physIPVperp2==1 | physIPVperp4==1
recode SevPhysIPVPerp 0/1=0 3=1
label variable SevPhysIPVPerp "Any severe IPV perpetration in the past year"
label define SevPhysIPVPerp 0 "No" 1 "Yes"
label values SevPhysIPVPerp SevPhysIPVPerp
tab SevPhysIPVPerp


/* mediators*/
gen physIPVvict1=E614
recode physIPVvict1 0=0 1/4=1 6/max=.
label variable physIPVvict1 "Had a physical fight in the past year which has resulted in their partner hitting, shoving, or throwing things at them"
label define physIPVvict1 0 "No" 1 "Yes"
label values physIPVvict1 physIPVvict1
tab physIPVvict1 E614

gen physIPVvict2=E615
recode physIPVvict2 1=1 2=0 3/max=.
label variable physIPVvict2 "Been cut, brusied or seriously injured in a fight with your partner"
label define physIPVvict2 0 "No" 1 "Yes"
label values physIPVvict2 physIPVvict2
tab physIPVvict2 E615

gen physIPVvict3=E710
recode physIPVvict3 0=0 1/4=1 6/max=.
```

```
label variable physIPVvict3 "Had a physical fight in the past year which has resulted in their partner hitting, shoving, or throwing things at them"

label define physIPVvict3 0 "No" 1"Yes"

label values physIPVvict3 physIPVvict3

tab physIPVvict3 E710


gen physIPVvict4=E711

recode physIPVvict4 1=1 2=0 3/max=.

label variable physIPVvict4 "Been cut, brusied or seriously injured in a fight with your partner"

label define physIPVvict4 0 "No" 1 "Yes"

label values physIPVvict4 physIPVvict4

tab physIPVvict4 E711


gen IPVvictPY1=.

replace IPVvictPY1=0 if E708==2

replace IPVvictPY1=0 if E612==2

replace IPVvictPY1=1 if physIPVvict1==1 | physIPVvict2==1 | physIPVvict3==1 | physIPVvict4==1

label variable IPVvictPY1 "Any physical or injurious IPV victimisation in the past year"

label define IPVvictPY1 0 "No" 1 "Yes"

label values IPVvictPY1 IPVvictPY1

tab IPVvictPY1


tab IPVvictPY1 physIPVvict1

tab IPVvictPY1 physIPVvict2

tab IPVvictPY1 physIPVvict3

tab IPVvictPY1 physIPVvict4


/* Severe IPV victimisation*/


gen SevPhysIPVvict=IPVvictPY

replace SevPhysIPVvict=3 if physIPVvict2==1 | physIPVvict4==1

recode SevPhysIPVvict 0/1=0 3=1

label variable SevPhysIPVvict"Any severe IPV victimisation in the past year"

label define SevPhysIPVvict 0 "No" 1 "Yes"

label values SevPhysIPVvict SevPhysIPVvict
```

```
tab SevPhysIPVvict

gen alcoholprob=E209A
recode alcoholprob 1=1 2/7=0 8/max=.
label variable alcoholprob "Respondent has a problem with drinking too much alcohol"
label define alcoholprob 0 "No" 1 "Yes"
label values alcoholprob alcoholprob
tab alcoholprob E209A
recode LSTA1NUM 6=.
gen Noofchildren= LSTA1NUM+LSTA2NUM
tab Noofchildren
gen age=M2BP01
gen income=IHTOT2
recode income 99999999=. 99999996=. 99999997=. 99999998=.
gen education=COMPLED
recode education 0/9=0 10/13=1 14/20=2 99=.
label variable education "Highest education qualification"
label define education 0 "None" 1 "High school qualifications" 2 "University degree or equivalent (and higher)"
label values education education
tab COMPLED education
tab education, gen (educationdummy)

** descriptives
summarize M2BP01, detail
tab CESDbinary
tab PhysicalIPVperpPY
tab SevPhysIPVPerp

svy: proportion PhysicalIPVperpPY
svy: proportion SevPhysIPVPerp
svy: proportion CESDbinary
svy: proportion PhysicalIPVperpPY if sexgender==1
svy: proportion PhysicalIPVperpPY if sexgender==2
svy: proportion SevPhysIPVPerp if sexgender==1
```

svy: proportion SevPhysIPVPerp if sexgender==2

svy: proportion PhysicalIPVperpPY if sexgender==1 & CESDbinary==0

svy: proportion PhysicalIPVperpPY if sexgender==1 & CESDbinary==1

svy: proportion PhysicalIPVperpPY if sexgender==2 & CESDbinary==0

svy: proportion PhysicalIPVperpPY if sexgender==2 & CESDbinary==1

svy: proportion SevPhysIPVPerp if sexgender==1 & CESDbinary==0

svy: proportion SevPhysIPVPerp if sexgender==1 & CESDbinary==1

svy: proportion SevPhysIPVPerp if sexgender==2 & CESDbinary==0

svy: proportion SevPhysIPVPerp if sexgender==2 & CESDbinary==1

*** mediation analysis - example

/*gen int_dep_subst = DepressionPY*SubMisuse

medeff (logit SubMisuse DepressionPY agedummy1 agedummy2) (logit PhysicalIPVperpPY DepressionPY SubMisuse int_dep_subst agedummy1 agedummy2), mediate (SubMisuse) treat(DepressionPY) sims(1000) seed(1) level(95) interact(int_dep_subst)*/

/* men physical IPV perp - alcohol use mediation analysis*/

gen int_dep_alc = CESDbinary*alcoholprob

medeff (logit alcoholprob CESDbinary income age educationdummy1 educationdummy2 Noofchildren) (logit PhysicalIPVperpPY CESDbinary alcoholprob int_dep_alc income age educationdummy1 educationdummy2 Noofchildren) if sexgender==1 [pweight=SAMWT], mediate (alcoholprob) treat(CESDbinary) sims(1000) seed(1) level(95) interact(int_dep_alc)

/* women - physical IPV perp - alcohol use mediation analysis*/

medeff (logit alcoholprob CESDbinary income age educationdummy1 educationdummy2 Noofchildren) (logit PhysicalIPVperpPY CESDbinary alcoholprob int_dep_alc income age educationdummy1 educationdummy2 Noofchildren) if sexgender==2 [pweight=SAMWT], mediate (alcoholprob) treat(CESDbinary) sims(1000) seed(1) level(95) interact(int_dep_alc)

***

/* men - physical IPV perp - IPV victimisation mediation analysis*/

gen int_dep_ipvvict = CESDbinary*IPVvictPY1

medeff (logit IPVvictPY1 CESDbinary income age educationdummy1 educationdummy2 Noofchildren) (logit PhysicalIPVperpPY CESDbinary IPVvictPY1 int_dep_ipvvict income age educationdummy1 educationdummy2 Noofchildren) if sexgender==1 [pweight=SAMWT], mediate (IPVvictPY1) treat(CESDbinary) sims(1000) seed(1) level(95) interact(int_dep_ipvvict)

/* women - physical IPV perp - IPV victimisation mediation analysis*/

medeff (logit IPVvictPY1 CESDbinary income age educationdummy1 educationdummy2 Noofchildren) (logit PhysicalIPVperpPY CESDbinary IPVvictPY1 int_dep_ipvvict income age educationdummy1 educationdummy2 Noofchildren) if sexgender==2 [pweight=SAMWT], mediate (IPVvictPY1) treat(CESDbinary) sims(1000) seed(1) level(95) interact(int_dep_ipvvict)

***

/* men - physical IPV perp - severe IPV victimisation mediation analysis*/

gen int_dep_sevipvvict = CESDbinary*SevPhysIPVvict

medeff (logit SevPhysIPVvict CESDbinary income age educationdummy1 educationdummy2 Noofchildren) (logit PhysicalIPVperpPY CESDbinary SevPhysIPVvict int_dep_sevipvvict income age educationdummy1 educationdummy2 Noofchildren) if sexgender==1 [pweight=SAMWT], mediate (SevPhysIPVvict) treat(CESDbinary) sims(1000) seed(1) level(95) interact(int_dep_sevipvvict)

/* women - physical IPV perp - severe IPV victimisation mediation analysis*/

medeff (logit SevPhysIPVvict CESDbinary income age educationdummy1 educationdummy2 Noofchildren) (logit PhysicalIPVperpPY CESDbinary SevPhysIPVvict int_dep_sevipvvict income age educationdummy1 educationdummy2 Noofchildren) if sexgender==2 [pweight=SAMWT], mediate (SevPhysIPVvict) treat(CESDbinary) sims(1000) seed(1) level(95) interact(int_dep_sevipvvict)

**** severe IPV perpetration

/* men sev physical IPV perp - alcohol use mediation analysis*/

*removed interaction term

medeff (logit alcoholprob CESDbinary income age educationdummy1 educationdummy2 Noofchildren) (logit SevPhysIPVPerp CESDbinary alcoholprob income age educationdummy1 educationdummy2 Noofchildren) if sexgender==1 [pweight=SAMWT], mediate (alcoholprob) treat(CESDbinary) sims(1000) seed(1) level(95)

/* women - sev physical IPV perp - alcohol use mediation analysis*/

medeff (logit alcoholprob CESDbinary income age educationdummy1 educationdummy2 Noofchildren) (logit SevPhysIPVPerp CESDbinary alcoholprob int_dep_alc income age educationdummy1 educationdummy2 Noofchildren) if sexgender==2 [pweight=SAMWT], mediate (alcoholprob) treat(CESDbinary) sims(1000) seed(1) level(95) interact(int_dep_alc)

***

/* men - sev physical IPV perp - IPV victimisation - mediation analysis*/

medeff (logit IPVvictPY1 CESDbinary income age educationdummy1 educationdummy2 Noofchildren) (logit SevPhysIPVPerp CESDbinary IPVvictPY1 int_dep_ipvvict income age educationdummy1 educationdummy2 Noofchildren) if sexgender==1 [pweight=SAMWT], mediate(IPVvictPY1) treat(CESDbinary) sims(1000) seed(1) level(95) interact (int_dep_ipvvict)

/* women - sev physical IPV perp - IPV victimisation - mediation analysis*/

medeff (logit IPVvictPY1 CESDbinary income age educationdummy1 educationdummy2 Noofchildren) (logit SevPhysIPVPerp CESDbinary IPVvictPY1 int_dep_ipvvict income age educationdummy1 educationdummy2 Noofchildren) if sexgender==2 [pweight=SAMWT], mediate(IPVvictPY1) treat(CESDbinary) sims(1000) seed(1) level(95) interact (int_dep_ipvvict)

***

/* men - sev physical IPV perp - severe IPV victimisation - mediation analysis*/

medeff (logit SevPhysIPVvict CESDbinary income age educationdummy1 educationdummy2 Noofchildren) (logit SevPhysIPVPerp CESDbinary SevPhysIPVvict int_dep_sevipvvict income age educationdummy1 educationdummy2 Noofchildren) if sexgender==1 [pweight=SAMWT], mediate (SevPhysIPVvict) treat(CESDbinary) sims(1000) seed(1) level(95) interact(int_dep_sevipvvict)

/* women - sev physical IPV perp - severe IPV victimisation - mediation analysis*/

medeff (logit SevPhysIPVvict CESDbinary income age educationdummy1 educationdummy2 Noofchildren) (logit SevPhysIPVPerp CESDbinary SevPhysIPVvict int_dep_sevipvvict income age educationdummy1

educationdummy2 Noofchildren) if sexgender==2 [pweight=SAMWT], mediate (SevPhysIPVvict) treat(CESDbinary) sims(1000) seed(1) level(95) interact(int_dep_sevipvvict)

4. Add Health code

```
/* Exposure variables*/

svyset [pweight=GSWGT4_2]


/* CESD*/

gen H4MH24reverse = H4MH24

recode H4MH24reverse 0=3 1=2 2=1 3=0 4/max=.

label define H4MH24reverse 3 "Never or rarely" 2 "Sometimes" 1 "A lot of the time" 0 "Most of the time or all of the time" 4 "Dont know, refused"

label values H4MH24reverse H4MH24reverse

tab H4MH24reverse H4MH24


gen H4MH25reverse = H4MH25

recode H4MH25reverse 0=3 1=2 2=1 3=0 4/max=.

label define H4MH25reverse 3 "Never or rarely" 2 "Sometimes" 1 "A lot of the time" 0 "Most of the time or all of the time" 4 "Dont know, refused"

label values H4MH25reverse H4MH25reverse

tab H4MH25reverse H4MH25


gen H4MH20reverse = H4MH20

recode H4MH20reverse 0=3 1=2 2=1 3=0 4/max=.

label define H4MH20reverse 3 "Never or rarely" 2 "Sometimes" 1 "A lot of the time" 0 "Most of the time or all of the time" 4 "Dont know, refused"

label values H4MH20reverse H4MH20reverse

tab H4MH20reverse H4MH20


tab H4MH20reverse

tab H4MH25reverse

tab H4MH24reverse

tab H4MH18

tab H4MH19

tab H4MH21
```

```
tab H4MH22

tab H4MH23

tab H4MH26

tab H4MH27


list AID if H4MH20reverse > 3

list AID if H4MH25reverse > 3

list AID if H4MH24reverse > 3

list AID if H4MH18 > 3

list AID if H4MH19 > 3

list AID if H4MH21 > 3

list AID if H4MH22 > 3

list AID if H4MH23 > 3

list AID if H4MH26 > 3

list AID if H4MH27 > 3


recode H4MH18 4/max=.

recode H4MH19 4/max=.

recode H4MH21 4/max=.

recode H4MH22 4/max=.

recode H4MH23 4/max=.

recode H4MH26 4/max=.

recode H4MH27 4/max=.


gen CESD = H4MH20reverse + H4MH25reverse + H4MH24reverse + H4MH18 + H4MH19 + H4MH21 + H4MH22
+ H4MH23 + H4MH26 + H4MH27


replace CESD = 4 in 474

replace CESD = 7 in 771

replace CESD = 8 in 1668

replace CESD = 5 in 2154

replace CESD = 12 in 2947

replace CESD = 3 in 2993

replace CESD = 6 in 3332

replace CESD = 11 in 3639
```

```
gen CESDbinary = CESD
recode CESDbinary min/7=0 8/max=1
label variable CESDbinary "Positive screen on CES-D 10 scale (binary)"
label define  CESDbinary 0 "No" 1 "Yes"
label values CESDbinary CESDbinary
tab CESDbinary CESD


/* Outcome variables */

/*Physical IPV perp*/
gen PhyIPVperpPY1 = H4RD22
recode PhyIPVperpPY1 0/1=0 2/7=1 8/max=.
label variable PhyIPVperpPY1 "Perpetration of pushing, shoving, throwing in PY"
label define PhyIPVperpPY1 0 "No" 1 "Yes" 2 "Dont know, refused"
label values PhyIPVperpPY1 PhyIPVperpPY1
tab PhyIPVperpPY1 H4RD22

gen PhyIPVperpPY2 = H4RD23
recode PhyIPVperpPY2 0/1=0 2/7=1 8/max=.
label variable PhyIPVperpPY2 "Perpetration of slapping, hitting, kicking in PY"
label define PhyIPVperpPY2 0 "No" 1 "Yes" 2 "Dont know, refused"
label values PhyIPVperpPY2 PhyIPVperpPY2
tab PhyIPVperpPY2 H4RD23

gen PhysicalIPVperpPY=.
replace PhysicalIPVperpPY = 1 if PhyIPVperpPY2 == 1
replace PhysicalIPVperpPY = 1 if PhyIPVperpPY1 == 1
replace PhysicalIPVperpPY = 0 if PhyIPVperpPY2 == 0 & PhyIPVperpPY1 == 0
label variable PhysicalIPVperpPY "Perpetration of physical IPV perpetration in PY"
label define PhysicalIPVperpPY 0 "No" 1 "Yes"
label values PhysicalIPVperpPY PhysicalIPVperpPY
tab PhysicalIPVperpPY
```

```
tab PhysicalIPVperpPY PhyIPVperpPY2
tab PhysicalIPVperpPY PhyIPVperpPY1

gen SevPhysIPVPerp = PhysicalIPVperpPY
replace SevPhysIPVPerp = 3 if PhyIPVperpPY2==1
tab SevPhysIPVPerp
recode SevPhysIPVPerp 0/1=0 3=1
label variable SevPhysIPVPerp "Perpetration of severe physical IPV in the past year"
label define SevPhysIPVPerp 0 "No" 1 "Yes" 2 "Dont know, refused"
label values SevPhysIPVPerp SevPhysIPVPerp
tab SevPhysIPVPerp

/* Mediator variables*/
/*IPV vict PY*/
gen IPVvict4 = H4RD18
recode IPVvict4 0/1=0 2/7=1 8/max=.
label variable IPVvict4 "IPV victimsation (pushing, shoving, throwing)"
label define IPVvict4 0 "No" 1 "Yes" 2 "Dont know, refused"
label values IPVvict4 IPVvict4
tab IPVvict4 H4RD18

gen IPVvict5 = H4RD19
recode IPVvict5 0/1=0 2/7=1 8/max=.
label variable IPVvict5 "IPV victimsation (slapping, kicking, hitting)"
label define IPVvict5 0 "No" 1 "Yes" 2 "Dont know, refused"
label values IPVvict5 IPVvict5
tab IPVvict5 H4RD19

gen IPVvict6 = H4RD21
recode IPVvict6 0/1=0 2/7=1 8/max=.
label variable IPVvict6 "IPV victimsation (sexual)"
label define IPVvict6 0 "No" 1 "Yes" 2 "Dont know, refused"
label values IPVvict6 IPVvict6
tab IPVvict6 H4RD21
```

```
gen IPVvictPY = .
replace IPVvictPY = 1 if IPVvict4  == 1 | IPVvict5 == 1 | IPVvict6 == 1
replace IPVvictPY = 0 if IPVvict4 == 0 & IPVvict5 == 0 & IPVvict6 == 0
label variable IPVvictPY "Past year IPV victimisation"
label define IPVvictPY 0 "No" 1 "Yes"
label values IPVvictPY IPVvictPY
tab IPVvictPY

/*Severe physical IPV victimisation*/
gen sevIPVvict1 = H4RD19
recode sevIPVvict1 0/1=0 2/7=1 96=. 98=.
label variable sevIPVvict1 "IPV victimisation (slapping, kicking, hitting)"
label define sevIPVvict1 0 "No" 1 "Yes" 2 "Dont know, refused"
label values sevIPVvict1 sevIPVvict1
tab sevIPVvict1 H4RD19

gen sevIPVvict2= H4RD20
recode sevIPVvict2 0/1=0 2/7=1 97=0 98=. 96=.
label variable sevIPVvict2 "IPv victimisation - had an injury e.g. a cut, sprain or bruise, in the past year"
label define sevIPVvict2 0 "No" 1 "Yes" 2 "Dont know, refused"
label values sevIPVvict2 sevIPVvict2
tab sevIPVvict2 H4RD20

gen SevPhysIPVvict = .
replace SevPhysIPVvict= 0 if sevIPVvict1  == 0 & sevIPVvict2 == 0
replace SevPhysIPVvict= 1 if sevIPVvict1  == 1 | sevIPVvict2 == 1
label variable SevPhysIPVvict "Severe IPV victimisation in the past year"
label define SevPhysIPVvict 0 "No" 1 "Yes"
label values SevPhysIPVvict SevPhysIPVvict
tab SevPhysIPVvict
tab SevPhysIPVvict H4RD19
tab SevPhysIPVvict H4RD20
```

```
/* alcohol*/

tab H4TO40 H4TO39

gen bingedrink1 = H4TO39

recode bingedrink1 0/3=0 97=0 4/6=1 96=2 98=2

label variable bingedrink1 "Number of days on which the participant has had a drink in the past month"

label define bingedrink1 0 "Fewer than 2 days a week" 1 "2 or more days a week" 2 "Don't know/refused'"

label values bingedrink1 bingedrink1

tab bingedrink1 H4TO39


gen bingedrink2 = H4TO40

recode bingedrink2 min/3=0 4/18=1 95=2 96=2 97=0 98=2

label variable bingedrink2 "Number of drinks usually consumed on a day of drinking in the last 30 days"

label define bingedrink2 0 "Fewer than 3 drinks" 1 "4 or more drinks" 2 "Don't know/refused/not asked"

label values bingedrink2 bingedrink2

tab bingedrink2 H4TO40


gen BingeDrink=.

replace BingeDrink = 1 if bingedrink1==1 & bingedrink2==1

replace BingeDrink = 0 if bingedrink1==0 & bingedrink2==0

replace BingeDrink = 0 if bingedrink1==1 & bingedrink2==0

replace BingeDrink = 0 if bingedrink1==0 & bingedrink2==1

label variable BingeDrink "Binge drinking, defined as 4 or more drinks on more than 2 days per week in the last
month"

label define BingeDrink 1 "Yes" 0 "No"

label values BingeDrink BingeDrink

tab BingeDrink

/* Variables for adjustment + descriptive analyses*/

gen age1=IYEAR4-H4OD1Y

tab age1

gen agegroup=age1

rename BIO_SEX4 sexgender

gen children=H4TR11

replace children= . if H4TR9 == 96

replace children= . if H4TR9 == 98

gen education=H4ED2
```

```
recode education 1/2=0 3/4=1 5=2 6=1 7/13=2 98=.

label variable education "Highest education qualification"

label define education 0 "No qualifications" 1 "High school / equivalent" 2 "Degree/equiv"

label values education education

tab H4ED2 education

tab education, gen(educationdummy)

gen income=H4EC1

recode income 1=2500 2=7499.5 3=12499.5 4=17499.5 5=22499.5 6=27499.5 7=34999.5 8=44999.5
9=624999.5 10=87499.5 11=124999.5 12=174999.5 96=. 98=.

label variable income "Approximate income based on mid point of bands"


** descriptives

summarize age1, detail

tab CESDbinary

tab PhysicalIPVperpPY

tab SevPhysIPVPerp

svy: proportion SevPhysIPVPerp

svy: proportion CESDbinary

svy: proportion PhysicalIPVperpPY

svy: proportion PhysicalIPVperpPY if sexgender==1

svy: proportion PhysicalIPVperpPY if sexgender==2

svy: proportion SevPhysIPVPerp if sexgender==1

svy: proportion SevPhysIPVPerp if sexgender==2

svy: proportion PhysicalIPVperpPY if sexgender==1 & CESDbinary==0

svy: proportion PhysicalIPVperpPY if sexgender==1 & CESDbinary==1

svy: proportion PhysicalIPVperpPY if sexgender==2 & CESDbinary==0

svy: proportion PhysicalIPVperpPY if sexgender==2 & CESDbinary==1

svy: proportion SevPhysIPVPerp if sexgender==1 & CESDbinary==0

svy: proportion SevPhysIPVPerp if sexgender==1 & CESDbinary==1

svy: proportion SevPhysIPVPerp if sexgender==2 & CESDbinary==0

svy: proportion SevPhysIPVPerp if sexgender==2 & CESDbinary==1

/* men physical IPV perp - alcohol use mediation analysis*/

gen int_dep_alc =  CESDbinary*BingeDrink

medeff (logit BingeDrink CESDbinary age1 income educationdummy1 educationdummy2 children) (logit
PhysicalIPVperpPY CESDbinary BingeDrink int_dep_alc age1 income educationdummy1 educationdummy2
```

children) if sexgender==1 [pweight=GSWGT4_2], mediate (BingeDrink) treat(CESDbinary) sims(1000) seed(1) level(95) interact(int_dep_alc)

/* women - physical IPV perp - alcohol use mediation analysis*/

medeff (logit BingeDrink CESDbinary age1 income educationdummy1 educationdummy2 children) (logit PhysicalIPVperpPY CESDbinary BingeDrink int_dep_alc age1 income educationdummy1 educationdummy2 children) if sexgender==2 [pweight=GSWGT4_2], mediate (BingeDrink) treat(CESDbinary) sims(1000) seed(1) level(95) interact(int_dep_alc)

***

/* men - physical IPV perp - IPV victimisation mediation analysis*/

gen int_dep_ipvvict = CESDbinary*IPVvictPY

medeff (logit IPVvictPY CESDbinary age1 income educationdummy1 educationdummy2 children) (logit PhysicalIPVperpPY CESDbinary IPVvictPY int_dep_ipvvict age1 income educationdummy1 educationdummy2 children) if sexgender==1 [pweight=GSWGT4_2], mediate (IPVvictPY) treat(CESDbinary) sims(1000) seed(1) level(95) interact(int_dep_ipvvict)

/* women - physical IPV perp - IPV victimisation mediation analysis*/

medeff (logit IPVvictPY CESDbinary age1 income educationdummy1 educationdummy2 children) (logit PhysicalIPVperpPY CESDbinary IPVvictPY int_dep_ipvvict age1 income educationdummy1 educationdummy2 children) if sexgender==2 [pweight=GSWGT4_2], mediate (IPVvictPY) treat(CESDbinary) sims(1000) seed(1) level(95) interact(int_dep_ipvvict)

***

/* men - physical IPV perp - severe IPV victimisation mediation analysis*/

gen int_dep_sevipvvict = CESDbinary*SevPhysIPVvict

medeff (logit SevPhysIPVvict CESDbinary age1 income educationdummy1 educationdummy2 children) (logit PhysicalIPVperpPY CESDbinary SevPhysIPVvict int_dep_sevipvvict age1 income educationdummy1 educationdummy2 children) if sexgender==1 [pweight=GSWGT4_2], mediate (SevPhysIPVvict) treat(CESDbinary) sims(1000) seed(1) level(95) interact(int_dep_sevipvvict)

/* women - physical IPV perp - severe IPV victimisation mediation analysis*/

medeff (logit SevPhysIPVvict CESDbinary age1 income educationdummy1 educationdummy2 children) (logit PhysicalIPVperpPY CESDbinary SevPhysIPVvict int_dep_sevipvvict age1 income educationdummy1 educationdummy2 children) if sexgender==2 [pweight=GSWGT4_2], mediate (SevPhysIPVvict) treat(CESDbinary) sims(1000) seed(1) level(95) interact(int_dep_sevipvvict)

**** severe IPV perpetration

/* men sev physical IPV perp - alcohol use mediation analysis*/

medeff (logit BingeDrink CESDbinary age1 income educationdummy1 educationdummy2 children) (logit SevPhysIPVPerp CESDbinary BingeDrink int_dep_alc age1 income educationdummy1 educationdummy2 children) if sexgender==1 [pweight=GSWGT4_2], mediate (BingeDrink) treat(CESDbinary) sims(1000) seed(1) level(95) interact(int_dep_alc)

/* women - sev physical IPV perp - alcohol use mediation analysis*/

medeff (logit BingeDrink CESDbinary age1 income educationdummy1 educationdummy2 children) (logit SevPhysIPVPerp CESDbinary BingeDrink int_dep_alc age1 income educationdummy1 educationdummy2 children) if sexgender==2 [pweight=GSWGT4_2], mediate (BingeDrink) treat(CESDbinary) sims(1000) seed(1) level(95) interact(int_dep_alc)

***

/* men - sev physical IPV perp - IPV victimisation - mediation analysis*/

*removed interactionterm

medeff (logit IPVvictPY CESDbinary age1 income educationdummy1 educationdummy2 children) (logit SevPhysIPVPerp CESDbinary IPVvictPY age1 income educationdummy1 educationdummy2 children) if sexgender==1 [pweight=GSWGT4_2], mediate (IPVvictPY) treat(CESDbinary) sims(1000) seed(1) level(95)

/* women - sev physical IPV perp - IPV victimisation - mediation analysis*/

medeff (logit IPVvictPY CESDbinary age1 income educationdummy1 educationdummy2 children) (logit SevPhysIPVPerp CESDbinary IPVvictPY int_dep_ipvvict age1 income educationdummy1 educationdummy2 children) if sexgender==2 [pweight=GSWGT4_2], mediate (IPVvictPY) treat(CESDbinary) sims(1000) seed(1) level(95) interact(int_dep_ipvvict)

***

/* men - sev physical IPV perp - severe IPV victimisation - mediation analysis*/

medeff (logit SevPhysIPVvict CESDbinary age1 income educationdummy1 educationdummy2 children) (logit SevPhysIPVPerp CESDbinary SevPhysIPVvict int_dep_sevipvvict age1 income educationdummy1 educationdummy2 children) if sexgender==1 [pweight=GSWGT4_2], mediate (SevPhysIPVvict) treat(CESDbinary) sims(1000) seed(1) level(95) interact(int_dep_sevipvvict)

/* women - sev physical IPV perp - severe IPV victimisation - mediation analysis*/

medeff (logit SevPhysIPVvict CESDbinary age1 income educationdummy1 educationdummy2 children) (logit SevPhysIPVPerp CESDbinary SevPhysIPVvict int_dep_sevipvvict age1 income educationdummy1 educationdummy2 children) if sexgender==2 [pweight=GSWGT4_2], mediate (SevPhysIPVvict) treat(CESDbinary) sims(1000) seed(1) level(95) interact(int_dep_sevipvvict)

5. 2014 APMS code

*VARIABLE GENERATION

*ResSex 1=male 2=female

svyset ipsu [pweight=weight_core], strata(istrata)

*drop participants who report never being in a relationship

keep if NevRel==1

*generating any depression variable

gen anydep=.

replace anydep =1 if milddep==1 | moddep==1 | sevdep==1

replace anydep=0 if milddep==0 & moddep==0 & sevdep==0

label variable anydep "any depression diagnosis"

label define anydep 0 "no" 1 "yes"

label values anydep anydep

tab anydep milddep

tab anydep moddep

tab anydep sevdep

```
*generating past year physical IPV perpetration variable
gen PhysicalIPVperpPY=.
replace PhysicalIPVperpPY = 1 if Act2Whn== 1 | Act3Whn==1
replace PhysicalIPVperpPY = 0 if Act2Whn== 2 & Act3Whn==2
replace PhysicalIPVperpPY = 0 if Act2Whn==-1 & Act3Whn==-1
label variable PhysicalIPVperpPY "Physical IPV perpetration in the past year"
label define PhysicalIPVperpPY 0 "No" 1 "Yes"
label values PhysicalIPVperpPY
tab PhysicalIPVperpPY
tab PhysicalIPVperpPY Act2Whn
tab PhysicalIPVperpPY Act3Whn

*generating severe past year physical IPV perpetration variable
gen SevPhysIPVPerp=.
replace SevPhysIPVPerp = 1 if Act3Whn==1
replace SevPhysIPVPerp = 0 if Act3Whn==2
replace SevPhysIPVPerp = 0 if Act3Whn==-1
label variable SevPhysIPVPerp "Severe physical IPV perpetration in the past year"
label define SevPhysIPVPerp 0 "No" 1 "Yes"
label values SevPhysIPVPerp SevPhysIPVPerp
tab SevPhysIPVPerp
tab SevPhysIPVPerp Act3Whn

*generating past year psychological IPV perpetration
gen PsychIPVperp=Act1Whn
recode PsychIPVperp 1=1 2=0 -1=0 8=0
label variable PsychIPVperp "Psychological perpetration in the past year"
label define PsychIPVperp 0 "No" 1 "Yes"
label values PsychIPVperp PsychIPVperp
tab PsychIPVperp Act1Whn

*generating past year sexual IPV perpetration
gen SexualIPVperpPY=.
replace SexualIPVperpPY = 1 if Act4Whn==1
```

```
replace SexualIPVperpPY = 0 if Act4Whn==2

replace SexualIPVperpPY = 0 if Act4Whn==-1

label variable SexualIPVperpPY "Sexual IPV perpetration in the past year"

label define SexualIPVperpPY 0 "No" 1 "Yes"

label values SexualIPVperpPY SexualIPVperpPY

tab SexualIPVperpPY Act4Whn


*ANALYSIS


*number and proportion of participants with depression

tab anydep

svy: proportion anydep


* number and proportion of participants reporting past year physical IPV perpetration

tab PhysicalIPVperpPY

svy: proportion PhysicalIPVperpPY


*sex disaggregated

tab PhysicalIPVperpPY if ResSex==1

tab PhysicalIPVperpPY if ResSex==2

svy: proportion PhysicalIPVperpPY if ResSex==1

svy: proportion PhysicalIPVperpPY if ResSex==2


*with/without depression

tab PhysicalIPVperpPY if ResSex==1 & anydep==1

tab PhysicalIPVperpPY if ResSex==1 & anydep==0

tab PhysicalIPVperpPY if ResSex==2 & anydep==1

tab PhysicalIPVperpPY if ResSex==2 & anydep==0


svy: proportion PhysicalIPVperpPY if ResSex==1 & anydep==1

svy: proportion PhysicalIPVperpPY if ResSex==1 & anydep==0

svy: proportion PhysicalIPVperpPY if ResSex==2 & anydep==1

svy: proportion PhysicalIPVperpPY if ResSex==2 & anydep==0
```

```
*number and proportion of participants reporting severe past year physical IPV perpetration
tab SevPhysIPVPerp
svy: proportion SevPhysIPVPerp

*sex disaggregated
tab SevPhysIPVPerp if ResSex==1
tab SevPhysIPVPerp if ResSex==2
svy: proportion SevPhysIPVPerp if ResSex==1
svy: proportion SevPhysIPVPerp if ResSex==2

*with/without depression
tab SevPhysIPVPerp if ResSex==1 & anydep==1
tab SevPhysIPVPerp if ResSex==1 & anydep==0
tab SevPhysIPVPerp if ResSex==2 & anydep==1
tab SevPhysIPVPerp if ResSex==2 & anydep==0

svy: proportion SevPhysIPVPerp if ResSex==1 & anydep==1
svy: proportion SevPhysIPVPerp if ResSex==1 & anydep==0
svy: proportion SevPhysIPVPerp if ResSex==2 & anydep==1
svy: proportion SevPhysIPVPerp if ResSex==2 & anydep==0
```

META-ANALYSIS CODE

Total effect:

```
metan Totaleffect LowerCI UpperCI, fixed label(namevar=Dataset) texts (150)
```

Direct effect:

```
metan Averagedirecteffect LowerCI UpperCI, fixed label(namevar=Dataset) texts (150)
```

Indirect effect:

```
metan Averagemediation LowerCI UpperCI, fixed label(namevar=Dataset) texts (150)
```
